# Searching for bioactive conformations of drug-like ligands with current force fields: how good are we?

**DOI:** 10.1186/s13321-017-0216-0

**Published:** 2017-05-15

**Authors:** Oya Gürsoy, Martin Smieško

**Affiliations:** Department of Pharmaceutical Sciences, Molecular Modeling, University of Basel, Klingelbergstrasse 50, 4056 Basel, Switzerland

**Keywords:** Force field, Conformer, Conformational search, Solvation, Solvent, Bioactive, Crystal pose-like

## Abstract

Drug-like ligands obtained from protein–ligand complexes deposited in the Protein Databank were subjected to conformational searching using various force fields and solvation settings. For each ligand, the resulting conformer pool was examined for the presence of the bioactive (crystal pose-like) conformation. Similarity of conformers toward the crystal-pose was quantified as the best achievable root mean squared deviation (RMSD, heavy atoms only). Analyzing the conformer pools generated by various force fields revealed only small differences in the likelihood of finding a crystal pose-like conformation. However, employing different solvents in the conformational search was found to be very important for achieving RMSDs below 1.0 Å. The best statistical values of likelihood were observed with a recently released force field covering a large portion of dihedral angles occurring in drug-like compounds in combination with the water as solvent. In order to enable computational chemists and modelers to efficiently use available software tools, we have additionally performed several focused analyses on ligands, grouped according to descriptors most relevant for the rational drug design.

**Electronic supplementary material:**

The online version of this article (doi:10.1186/s13321-017-0216-0) contains supplementary material, which is available to authorized users.

## Background

In the last two decades, computational methods have found an established role in the rational drug design process due to their invaluable help in interpreting experimental results (e.g. NMR, X-ray), generating novel ideas based on theoretical models and navigating research steps with predictive tools. A large portion of the “rational” within computational methods stem from analyzing of structural information from X-ray crystal and NMR structures of ligand–protein complexes. To date, the most important repository of such structures—the Protein Data Bank (PDB) [1]—houses around 120,000 biological macromolecular structures [2] (Accessed July, 2016) and with each new entry improves our understanding of the biologically and medicinally relevant phenomena at the atomic level. As approximately one half of the entries have been deposited in the last 5 years, there is a need for periodic re-evaluation of our knowledge on both macromolecular targets as well as ligands interacting with them.

In the rational drug design, computational chemists focus on ligand properties including conformational ones and fine tune ligand structure and pre-organization with the aim to minimize energetic penalties associated with undesired flexibility, sub-optimal arrangement of functional groups interacting with the protein binding site or unwanted internal stabilization. When the 3D structure of the target protein is known (structure-based design), a modern lead optimization process often involves identifying reasonable binding poses and—most desirably—also the bioactive one using molecular docking and scoring. As accounting for a full molecular flexibility of both ligand and protein simultaneously is extremely complex and currently far beyond our computational capacity, some docking approaches pre-generate reasonable ligand conformers in a conformational search and then try inserting them into the (either rigid or flexible) binding site of the target protein looking for the best possible complementarity of steric and electronic properties [3–8]. As an alternative to this approach, some protocols employ on-the-fly conformer generation in the receptor’s binding cavity [9, 10] or rely on the fragment based docking with a full rotational flexibility of dihedral angles [11, 12].

Favorable ligand conformations, resulting from a conformational search, are of key importance also in the ligand based design, when the structural information about the target is scarce or non-existent and therefore the binding hypothesis assuming the key-lock complementarity has to be derived from known ligands sharing a common 3D arrangement of functional groups (pharmacophore) [13, 14].

The need for low-energy conformers, especially within the context of finding the bioactive conformation, has been long recognized. Therefore a number of different search algorithms and sampling methods have been developed and implemented in protocols for generating conformers of small (drug-like) molecules [15–17]. Some tools rely on a systematic approach (i.e. CORINA [18, 19], ConfGen [3], OMEGA [20, 21])and some use a stochastic approach (i.e. BALLOON [22], RDKit [23]). Commonly used protocols such as Catalyst [24], MOE [25] and MacroModel [26] implement both approaches [15, 27]. Significance of the conformational sampling and challenges in finding the bioactive pose among an ensemble of generated conformers were previously discussed in several studies [14, 28–32].

During a conformational search, the geometry optimization (energy minimization) of conformers is driven by a force field. A well-parameterized force field is thus a key prerequisite for ensuring that the resulting conformer pool includes the bioactive conformation (or one very similar to it). As the conformer energy is often the most important criterion for selecting, all force field terms for calculating energies (bonds, angles and dihedral angles, electrostatic and van der Waals (vdW), solvation contributions) need to be accurate and mutually balanced. The performance of some force fields has already been assessed for specific sets of ligands or small peptides [33–35]. In this study, we investigate several frequently used force fields (implemented in a popular commercial software bundle [26]) for their ability to generate and rank bioactive conformations of a chemically very diverse set of drug-like ligands.

## Methods

In order to perform focused analyses using in-house scripts and compiled software in a CPU-efficient manner, a local mirror of the PDB was created (October 1, 2014). Crystal structures of macromolecular targets, except of those containing nucleic acids, with at least one co-crystallized small ligand and the resolution better than 1.6 Å (arbitrary threshold introduced to ensure solid quality of the structural data) were selected for further analyses. As this study focuses on issues associated with the application of rational computer methods in drug design, drug-likeness of ligands found in such crystal structures has been evaluated from several aspects.

### Ligand selection

Our software analyzed composition of ligands including only those comprising of biogenic elements (C, H, N, O, S, P, F, Cl, Br, and I). Ligands covalently bonded to protein or those having unreasonably close contacts with water molecules were discarded (distance of the water oxygen atom toward any ligand heavy atom shorter than the sum of their vdW radii multiplied by a factor f; for carbon atoms f = 0.891, for all others f = 0.8). In case of alternate orientations for a single ligand, only the conformation with the highest occupancy was considered. If occupancy values for multiple orientations were equal, coordinates of the first encountered orientation (as appeared in the PDB file) were taken into account. Crystal symmetry mates were generated (using a script from the Schrodinger library) for the asymmetric protein units and ligands in the vicinity of a neighboring crystal mate (within 5.0 Å from any ligand heavy atom), which might affect their pose and conformation by “artificial” packing effects, i.e. effects which would not occur in a free isolated protein molecule, were discarded (1743 structures in total).

### Ligand preparation

Hydrogen atoms were added to ligands based on the ligand templates from the curated library of ligands [36, 37] (Accessed: November 28, 2015). The most probable protonation state for each ligand was predicted by Epik [38], at the pH used for crystallization (information on pH was read from the PDB file header), using water as solvent. Ligands containing multiple charged groups, with the total charge smaller than −3 or larger than +3 were excluded from any further analyses.

Drug-likeness of ligands within this study was evaluated based on descriptors proposed by Lipinski [39] and Veber [40], allowing for some tolerances for upper and lower limits. Ligand efficiency or potency descriptors could not be used for defining drug-likeness, as there are no standardized data available. All ligands stem from crystal structures so their sufficient water solubility is assumed. Number of rotatable bonds (#RotB), molecular weight (MW), and molecular surface areas (3D: MSA, PSA) were calculated by our program and crosschecked with the values provided by FaF-Drugs2 [41] (for #RotB) and QikProp [42]. Octanol–water partition coefficient (LogP o/w) was estimated and the number of heavy atoms (#HA) was calculated by QikProp. Polar surface area (2D-PSA) was computed by OpenBabel [43, 44], where the total PSA equals to the sum of atomic contributions. Descriptors, which were used for elimination and their threshold values, can be found in Additional file 1.

### Further inclusion criteria

Several small molecules, such as crystallizing agents (e.g. citrate, PEGs, carboxylic acids) and prosthetic groups, i.e. vitamins, saccharides, or heme units, are present in the PDB with increased frequency. In a PDB file, these molecules are defined within the HETATM record along with small molecule ligands, but for the purpose of our study they were not classified as drug-like.

To avoid the bias caused by repeated occurrence of such structures in the data set, an occurrence analysis (i.e. how many times a particular ligand is found in a protein–ligand complex) was performed for each ligand. Mean value and standard deviation of the ligand occurrence in the dataset were calculated (μ = 1.80, σ^2^ = 1.33). The threshold value was obtained by adding one standard deviation to the mean (3.13) and rounding it to the next higher integer value (4). Therefore, ligands that appeared more than four times in the raw data set were excluded to ensure diversity of data samples for unbiased statistical analyses. The PDB IDs of these excluded structures can be found in Additional file 1.

After the elimination due to molecular descriptors (510 ligands) and frequency analyses (169 ligands; 14 unique structures), the final set of 809 ligands have maximum 15 rotatable bonds, stay within the molecular weight range of 150–650 and consist of 10–150 heavy atoms. The PDB IDs of the 809 structures included in the final set can be found in Additional file 1 (the structures are also available in additional/supporting files).

### Conformational search

A two-step conformational search was carried out for the final set of ligands by MacroModel [26]. The first step was incorporated into the search protocol with the aim to remove possible positive structural bias associated with the fact that the crystal conformation was used as input. In the first step, choosing OPLS_2005 [45] as the force field and GB/SA water model for the implicit solvation, a pool of conformers was generated for each ligand with mixed MCMM/Low-Mode conformational algorithm, by accepting all conformers with energies up to 50.0 kcal/mol from the global minimum. The maximum number of Monte Carlo steps was set to 5000. At maximum, 1000 conformers were saved for every individual ligand.

Next, heavy-atom RMSDs were calculated between each conformer and the original crystal conformation (n.b. all RMSD calculations in this study are symmetry corrected, taking into account the structural automorphism; e.g. in case of a molecule containing a para-substituted benzene ring, the RMSD between two conformations is zero if these two conformations differ only by a 180° flip of the ring around the linker bond). The conformer with the highest RMSD from the crystal conformation, i.e. the most dissimilar one, was saved to a separate file and used as the input for the second step of conformational search.

The second—production—step of conformational search was performed using four different force fields—OPLS3 [46], OPLS_2005 [45], MMFF94s [47] and AMBER* [48–50], with the same operating parameter settings as in the first step, except that the energy window was set to 5.0 kcal/mol, as in this step the low-energy conformers were of interest.

To evaluate the effect of different solvents, conformational searches were also done using octanol and chloroform. This solvent effect analysis follows the assumption that, e.g. a polar compound in a relatively hydrophobic protein binding site may adopt a collapsed bioactive conformer (self-folded, stabilized by intramolecular interactions), which would be probably more similar to conformers optimized using the octanol or chloroform as solvent. If optimized in water, such a polar compound would benefit from favorable interactions with the solvent environment and its conformers would extend to maximize the polar interactions, resulting in a conformer pool with members likely dissimilar from the bioactive conformer.

Using only heavy atoms, RMSD values were calculated between all conformers found in the production step and the original crystal conformation. The conformer with the lowest RMSD from the crystal conformation, i.e. the most similar one, was used in the following analyses and statistical comparisons.

### Employing MD simulations with explicit solvent for conformer minimization

Random “raw” (not minimized) conformers generated by the MacroModel conformational search engine were used as input for a complex minimization protocol aiming at mimicking “real world” conditions, i.e. those, which a free, unbound ligand experiences in an aqueous environment represented by explicit water molecules.

For all ligands, every single input conformer was placed in a TIP3P water-filled periodic boundary system (10.0 Angstrom cut-off in each Cartesian direction) and minimized (convergence criteria 0.5 kcal/mol). Next, a short 24 ps MD simulation using the NPT ensemble was performed. In contrast with typical approaches where MD is employed to examine (sample) ligand’s dynamical freedom, our short MD run was included in the protocol only to allow for the conformer to adjust its shape and for water molecules to find their optimal position and orientation with respect to the solute and neighboring waters.

After the MD simulation, the resulting periodic boundary system was minimized again to eliminate small geometry distortions using the Desmond [51–53] minimizer and finally also using the MacroModel minimizer (because of tighter convergence criteria). The resulting conformers were ranked based on their energies.

Due to explicit solvent conditions, the convergence of minimization was much worse than in case of implicit solvent, therefore a larger energy window was used (however, the final conformer counts were comparable with those obtained in conformational searches employing implicit solvent conditions).

### Chemometric analysis

For all ligands included in this study, the most important physico-chemical descriptors related to rational drug design have been calculated using QikProp [42] computer program and used for classification.

## Results and discussion

This study aims at providing answers to several important questions relevant for the modern drug design: Is it possible to computationally generate crystal-pose-like (aka bioactive) conformations of drug-like molecules to be used for rational design purposes, e.g. for ligand docking or pharmacophore hypothesis based on ligands’ distance matrix? Are increased computational costs associated with “on the fly” ligand flexibility in docking justified or is it enough to prepare a pool of low energy conformers and reduce a rather complex docking procedure to “simple” accommodating of prepared conformers (without further modifying their geometry) into the protein binding site (with side-chains being either rigid or flexible)?

In the search for answers to these questions, statistical analyses were performed on conformer pools generated in conformational searches using various force fields (OPLS3, OPLS_2005, MMFF94s, AMBER*) and solvent conditions (implicit solvation models: water, octanol, and chloroform; explicit model: water; gas phase) for a set of drug-like ligands. In order to provide modelers and medicinal chemists with as much rational information as possible, detailed analyses were performed on ligands clustered according to various constitutional and physico-chemical descriptors.

The final dataset consisted of 809 drug-like molecules. Averaged properties over the whole dataset were as follows: number of rotatable bonds #RotB was 5.4 (min = 0, max = 15), molecular weight MW was 313.1 (min = 150, max = 648), number of heavy atoms was 21.6 (min = 10, max = 47), calculated LogP was 1.3 (min = −5.0, max = 7.7), PSA was 103.0 Å^2^ (min = 0 Å^2^, max = 287 Å^2^).

### Interpretation of conformational similarity based on key molecular descriptors

#### Number of rotatable bonds

The number of rotatable bonds (#RotB) was chosen as the main descriptor for the ligand classification, since variation of dihedral angles along rotatable bonds allows a compound to adopt distinct molecular shapes and spatial distribution of its functional groups, which play the key role for molecular recognition (compound selectivity) and interaction pattern with macromolecular targets. #RotB is also the descriptor showing the highest correlation to the RMSD of the most-crystal-like conformation (best RMSD OPLS 3.0: *r* ^2^ = 0.546, n = 809).

Compounds having more than 15 rotatable bonds were excluded from further analyses, as with such an extreme conformational freedom (flexibility) the probability of finding the global minimum conformation and reasonable low energy conformers decreases drastically even with the most advanced search algorithms. Moreover, too flexible compounds are not attractive from the point of rational drug design as they lose on their drug-likeness, due to a low expected biological availability after oral intake and large entropic penalties upon binding with the target macromolecule.

**Fig. 1:**
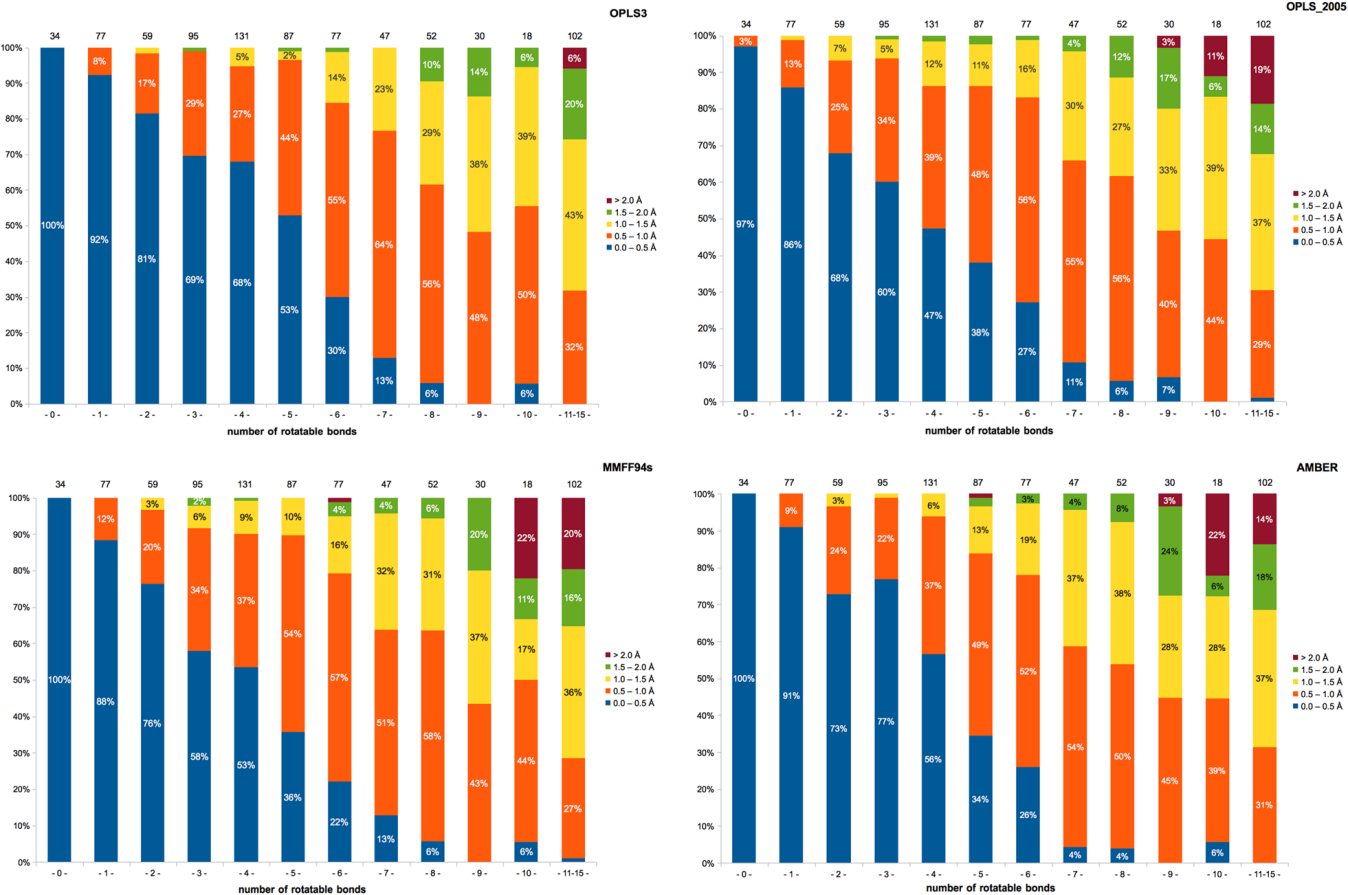
Comparison of results obtained for different force fields depending on ligand flexibility (#RotB)

Based on #RotB, the data set was divided into four groups denoting their flexibility as: low (0 ≤ #RotB ≤ 3), fair (4 ≤ #RotB ≤ 6), high (7 ≤ #RotB ≤ 10) and extreme (11 ≤ #RotB ≤ 15). Table 1 and Fig. 1 contain results for each of the four force fields with implicit solvent (water) and energy window of 5.0 kcal/mol.

**Table 1:** Fraction (%) of ligands similar to the reference crystal pose based on RMSD

#RotB	#Compounds	0–0.5 Å (%)				0–1 Å (%)			
		OPLS3	OPLS 2005	MMFFs	AMBER*	OPLS3	OPLS 2005	MMFFs	AMBER*
0	34	100	97	100	100	100	100	100	100
1	77	92	86	88	91	100	99	100	100
2	59	81	68	76	73	98	93	97	97
3	95	69	60	58	77	99	94	92	99
4	131	68	47	53	56	95	86	90	94
5	87	53	38	36	34	97	86	90	84
6	77	30	27	22	26	84	83	79	78
7	47	13	11	13	4	77	66	64	59
8	52	6	6	6	4	62	62	63	54
9	30	0	7	0	0	48	47	43	45
10	18	6	0	6	6	56	44	50	44
11–15	102	0	1	1	0	32	30	28	31

For more than 90% of ligands in the cluster of rigid and low flexible compounds (#RotB ≤ 3; n = 265), all force fields were able to generate a pool of conformers in which at least one conformer had a low RMSD (≤1.0 Å) from the reference crystal conformation (throughout the text, the term crystal conformation refers to the conformation of a particular ligand found in the protein binding site, not to be confused with a conformation of the ligand alone, e.g. in a small molecule crystal from the Cambridge Structural Database [54]).

Regardless of the force field used, at least 78% of the ligands (n = 295) with 4 to 6 rotatable bonds had a conformer within the RMSD range of 0–1.0 Å (in this #RotB cluster, the average score over all force fields is 87%). This is an encouraging observation, as most of the drug-like molecules are expected to fall into this range of rotatable bond counts, meaning that all force fields are well-suited for modeling of leads or drug candidates, provided they meet also other drug-likeness criteria specified earlier.

Not surprisingly, finding a conformation with a low RMSD from the reference crystal conformation was less probable for highly flexible ligands (n = 147; 7 ≤ #RotB ≤ 10). Still, with all force fields, at least one conformer with an RMSD lower than 1.0 Å within the 5.0 kcal/mol energy window from the global minimum could be generated for about one half of the ligands (average score over all force fields is 55%) even with as much as 10 rotatable bonds. In the group of extremely flexible compounds (n = 102; RotB > 10), the probability of finding a bioactive-like conformation in the conformer pool was only around 30%.

Overall, within ligands featuring up to 10 rotatable bonds, the conformers, which showed highest similarity to their crystal reference conformations (RMSD ≤ 1.0 Å), were generated most frequently by the OPLS3 force field.

Quite surprisingly, a rather narrow parameter set of the AMBER* force field focusing primarily on proteins and nucleic acids instead of on small molecules performed well on our PDB ligand selection (especially with less flexible ligands), closely followed by the MMFF94s and OPLS_2005 force fields (ranking by the total sum of the best RMSDs; Additional file 1: Table S6).

As can be seen, the number of ligands in each cluster according to rotatable bond counts was not equal. For example, while there were 131 ligands with four rotatable bonds in the data set, only 18 ligands had ten rotatable bonds. This fact has to be kept in mind when interpreting and implementing obtained statistical data for clusters with a relatively low number of ligands.

To have a better idea about the profile of the ligands which have a conformer with an RMSD smaller than 1.0 Å with respect to the crystal conformation (OPLS_2005, solvent: water), co-variation of some other descriptors were also assessed within different ranges of rotatable bond counts (Additional file 1: Table S1).

Rigid and the least flexible ligands (#RotB: 0–3) were likely to have a low molecular weight and less heavy atoms and quite as expected the opposite situation was likely for the most flexible ligands (#RotB: 10–15). Calculated values showed that a high fraction (67%) of the ligands in the low-flexibility cluster had MW lower than 250 Da and less than 15 heavy atoms (57%), whilst for the most flexible cluster, 20% of the ligands had MW lower than 250 Da and 11% had less than 20 heavy atoms. Effect of changes in MW and #HA was less pronounced for ligands which had a fair and a high flexibility (#RotB: 4–10).

Molecular weight, number of heavy atoms and octanol–water partition coefficient were additional molecular properties associated with drug-likeness of molecules. Therefore, generated conformers (with best possible RMSD from the crystal reference) were classified also on these properties. These analyses offer a more comprehensive description of the chemical profile of the data set. Conformers obtained with the OPLS3 force field (solvent: water, energy window: 5.0 kcal/mol) were used for these analyses.

#### Molecular weight

Depending on the molecular weight (Additional file 1: Fig. S1) ligands were divided into four groups: the highest number of ligands (311 ligands) fell into the MW range of 150–250 and the least populated was the range between 450 and 650 Da (117 ligands). As much as 97% of the conformers from the lowest MW cluster could be generated with an RMSD below 1.0 Å. For the second (MW: 250–350) and third (MW: 350–450) cluster, a smaller fraction of their ligands had at least one conformer below 1.0 Å compared to the first cluster (87 and 72%, respectively). This suggests that the probability of obtaining a low RMSD conformer (<1.0 Å) with respect to the crystal conformation for ligands which stay within the broad MW range between 150 and 450 is expected to be higher than ~70%, whereas for the last cluster (MW: 450–650) this probability was significantly lower (46%).

It was previously reported that MW is also expected to increase as the #RotB increases [29]. To analyze this trend within our data set, the change of #RotB was tabulated for the MW ranges mentioned above (Additional file 1: Table S2). The gradual change with respect to rotatable bond counts was analogous for compounds in all MW clusters. Thus, the statistical data obtained for ligands in our data set also proves that the MW increases gradually as ligands become more flexible (#RotB increases).

#### Number of heavy atoms

The relationship between heavy atom counts and best achievable RMSDs was also examined on the very same conformer set as mentioned above (OPLS3, water, 5 kcal/mol energy window). The ranges for clustering based on the number of heavy atoms were arbitrarily defined as: 10–14, 15–20, 21–25, 26–30 and greater than 30 (Additional file 1: Fig. S2). At least 91% of the ligands with up to 20 heavy atoms have at least one conformation with smaller RMSD than 1.0 Å in their low energy conformer pool. For the same RMSD range, a slight decrease was identified for the probability of finding a crystal-like conformer was identified for ligands with 21–30 heavy atoms (75%). However, a substantial increase of RMSD values was observed for ligands featuring more than 30 heavy atoms (47%).

### Solvent effects based on octanol–water partition coefficient (implicit solvation model)

A special attention was paid to examining effects of various solvents on the conformer generation. The data set was split into seven groups based on the octanol–water partition coefficient (LogP) (Additional file 1: Figs. S3–S4) and conformers were generated using OPLS3 force field with varying solvents (water, octanol, or chloroform), but keeping all other settings unchanged.

Theoretically, in a search for the bioactive conformation, ligands with a hydrophilic character (expressed as a low LogP value) and which—assuming the key-lock matching theory—likely bind to polar binding sites shall score better (in terms of a low RMSD to the reference), when the conformational search and especially minimization there within is performed using a polar solvent model (water) than when it is done in a low dielectric solvent like octanol.

In analogy for lipophilic ligands (high LogP values) presumably binding to lipophilic binding sites, a higher similarity of conformers and crystal poses would be expected, when searched using octanol as solvent. In such case, lipophilic groups preferring the interaction with the lipophilic solvent environment would drive the geometry optimization (minimization) toward extended conformations in order to maximize their contact (solvent-accessible) surface. On the other hand, using the water solvation model with a ligand having lipophilic groups might result in (hydrophobically) collapsed conformations due to their tendency to minimize their exposure to the polar solvent.

Finally, simulating a hydrophilic ligand, featuring multiple H-bond donors and acceptors in a lipophilic solvent, would promote increased formation of intramolecular hydrogen bonds, as no (or too little) competing H-bonding counterparts are provided from the solvent. In any case, internally over-stabilized conformations generated in the conformational search would be less suitable as pose candidates, since their ability to form directional intermolecular H-bonding or hydrophobic interactions within the binding site would be compromised.

The analysis of results did not show any clear trend indicating improved probabilities for a particular combination of ligand’s calculated LogP and used solvent. In most cases, conformers generated using octanol were less crystal-pose like with the exception of one LogP group (4.0–4.99, RMSD range <1.0 Å). However, the plot of the best RMSDs for all ligands with the same force field but different solvent (Additional file 1: Fig. S5) showed that solvent effects are mixed, i.e. conformers of some ligands benefit (points below the diagonal indicate ligands whose best RMSD toward reference is better in octanol than in water), but a slight majority actually worsen (points above the diagonal) their crystal-pose likeness. This observation suggests that each ligand has its own sensitivity towards varying solvents, which cannot be reduced to a simple LogP parameter. Whether such mixed effects are related to the nature of the binding site will be the subject of our further studies.

### Impact of explicit solvation model

To complement this type of ligand-solvent analysis, we generated conformers of each ligand (starting from same random conformations as in all other searches) also using explicit solvent conditions. Initial minimization, followed by an MD simulation and a final re-minimization should ensure that both ligand and water molecules surrounding it adopt the most favorable mutual orientation allowing for a more realistic treatment of particular solvent effects (e.g. bridging waters, hydrophobic effects). Despite large computational costs (~602 k MD simulations!), no significant improvement of similarity to crystal pose conformations was observed in general. Just like with the implicit octanol solvation, conformations of some ligands became more crystal-like, but a slight majority of ligands suffered from an increase in the best achievable RMSD value.

### General observations on solvent effects

Employing some kind of (either implicit or explicit) solvation model in a conformational search seems to have a large beneficial impact on the ligand geometries, if similarity to co-crystalized conformations is the primary objective.

In our study, both conformational searches featuring ligand minimization in the gas phase (once with the OPLS3 force field in MacroModel, once with the MMFF94s force field as implemented in OpenBabel) led to largely worsened results, i.e. the best RMSDs were much higher (i.e. conformers were not crystal pose-like) when compared to those obtained from “solvated” minimizations (Additional file 1: Table S6).

Interestingly, the descriptor polar surface area (PSA) of a ligand related to solvation showed an apparent negative correlation with the crystal-pose likeness (Additional file 1: Fig. S6). While for as much as 64% of ligands with a low PSA (below 70 Å^2^) at least one very crystal-pose like conformer (RMSD < 0.5 Å) can be found in the conformer pool, this fraction drops to 10% with ligands featuring a large PSA above 210 Å^2^. However this observation is very likely biased by the fact that with an increasing PSA of ligands, their overall conformational complexity (more rotatable bonds, more heavy atoms) might rise as well.

But, if PSA is corrected in respect to the total SASA (Additional file 1: Figs. S7–S8), the trend gets weaker (if percentages cover RMSDs below 1.0 Å) or even reversed (if only those below 0.5 Å are counted), meaning that for compounds with a higher fraction of polar surface area (>0.4), crystal-like conformers are generated more easily (57% of ligands have a conformer with the best RMSD below 0.5 Å) than for those being less polar or completely apolar (41% with ligands having PSA/SASA below 0.1).

### Effect of increased energy window on conformational search results

Statistically, enlarging the energy window (EW) for accepting conformers in a conformational search may be directly associated with an increased probability of finding a matching conformer in the resulting conformer pool.

If the crystal pose-like conformation, on the potential energy surface lies high above the global minimum energy conformer taken as a basis for positioning of the energy window, which if too small, would not include that particular conformer in the final pool. However at the same time, the number of conformers accepted for each molecule in the data set would increase requiring more computational time for their further processing (typically docking and scoring).

To examine these effects on our dataset of drug-like compounds, EW was lifted to 10.0 kcal/mol for conformational searches with OPLS3, OPLS_2005 and MMFF94s force fields. While with the 5.0 kcal/mol EW 85% of molecules in the data set had conformations with RMSD smaller than 1.0 Å, with 10 kcal/mol EW an increase to 89.7% was observed. However the total number of all conformations was 3.4 times higher and the overall RMSD improvement was not significant (average RMSD improvement per compound: 0.05 Å).

Increased number of conformers was obviously most prominent for compounds having larger #RotB. Therefore, we conclude that a relatively small increase in the success rate does not appropriately justify for a large increase of processing costs if EW of 10.0 kcal/mol is used (e.g. in high-throughput virtual screening), especially with very flexible compounds.

Still, if the maximum accuracy within a narrow set of ligands is desired (lead optimization), enlarging EW might help to increase likelihood of finding crystal-like conformers. In our dataset, 35.1% of ligands (OPLS3, solvent: water) had the bioactive pose within 1.0 Å from the global minimum structure, which supports the idea of setting a reasonable energy cut-off for selecting conformers to the final pool (For other force field-solvent combinations: Additional file 1: Table S7).

### Using NMR models as input for conformational analyses

Throughout this study, the RMSD on heavy atoms is used as a measure of similarity of a computationally generated conformer and the crystal pose of the corresponding ligand. Calculating the RMSD has been previously criticized, however, it still remains the most frequently used parameter for describing similarity of conformers [16, 33, 55–57].

In order to answer the question, how appropriate and sensitive such RMSD evaluation is in our study and how its magnitude compares with the experimentally observed conformational fluctuation, we analyzed also ligand models determined by the nuclear magnetic resonance (NMR) deposited within the PDB.

**Fig. 2:**
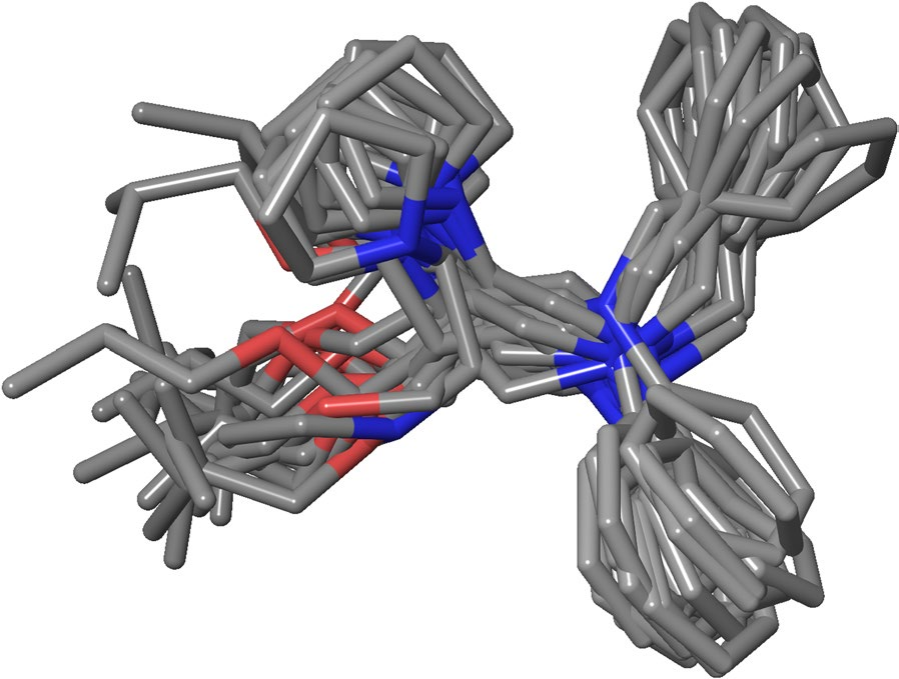
Fifteen NMR models of the same ligand (extracted from the PDB entry 1LXF)

Of identified 120 structures determined by the solution NMR, 75 had an ensemble of multiple models (5–60) and fulfilled the same criteria used for selecting ligands from crystal structures (150 < MW < 650, #RotB < 15, 10 < #HA < 150). For each ligand, inter-model RMSDs were calculated (each model vs. all other models), followed by a determination of the arithmetic mean. Average inter-model RMSD values varied between 0.004 and 1.265 Å and show how much natural fluctuation occurs within ligands bound to protein (Fig. 2).

This analysis seems to justify setting 1.0 Å as the upper limit for describing a good conformational similarity of conformers and crystal poses (acceptable for further processing—scoring or MD simulation), as conformational fluctuation of this magnitude is indeed normal.

As expected, plotting the distribution of inter-model RMSD values against the #RotB descriptor (Additional file 1: Figs. S9–S10) confirms that with an increased conformational freedom of the ligand (higher #RotB) the chance of more pronounced geometry fluctuations rises—a fact that should be reflected upon when evaluating RMSD values for larger and more flexible compounds.

This can be further supported by the statistical analysis of RMSD increase per rotatable-bond (crystal structures data set; conformer to crystal pose conformation comparison), showing that each rotatable bond causes “worsening” of the best achievable RMSD by 0.13(± 0.02) Å with only tiny differences among various force fields, if water is used as solvent (Additional file 1: Table S6).

It is also worth mentioning that no correlation could be detected between the number of models an NMR structure has and the average inter-model RMSD value (Additional file 1: Fig. S11), thus the results are not biased this way.

### Impact of charged groups

Presence of the charged groups in ligands (n.b. in our study the protonation state is assigned according to the pH of the crystallizing solution) and their effect on conformers were investigated, too. Most of the ligands (77%) in our data set had either none or one charged group in their structure. Only a few ligands (6 in total) had up to 5 charged groups, which—besides being extremely polar—were also considered less drug-like due to their high molecular weight or high flexibility.

As a general trend for all force field-solvent combinations, as the number of charged groups on ligands increased, the similarity of their conformers to their crystal reference structures decreased (higher RMSD per compound, (Additional file 1: Table S3). This observation is related to the fact that charged groups can interact (attractively/repulsively) with each other over large distances (Coulomb’s law) and the associated electrostatic interaction energy is relatively high compared to other terms (e.g. torsional energy, hydrophobic interaction).

Comparison of per compound RMSDs between different solvents revealed that the best performance was achieved when water was the solvent, independent of the employed force field (Additional file 1: Table S3). Since the protonation states of ligands were assigned using water as solvent, this finding led us toward the idea of using the neutralized form of ligands for performing the conformational search with non-aqueous solvents. The results showed that per compound RMSD values were considerably better for all force field-solvent combinations; more significantly when chloroform and octanol were used as solvent (Additional file 1: Table S4).

For molecules that are large and flexible, the presence of charged groups can result in distorted conformations, as usually there is no counter-balancing charge present in the environment during conformational searching. Therefore, when employing neutral species, the improvement was bigger for the large and flexible molecules with several charged groups. Zwitterionic and flexible compounds, in which the oppositely charged groups were located distally, showed dramatic changes in the similarity of the output conformations (of the conformational search) to their crystal pose conformations.

**Fig. 3:**
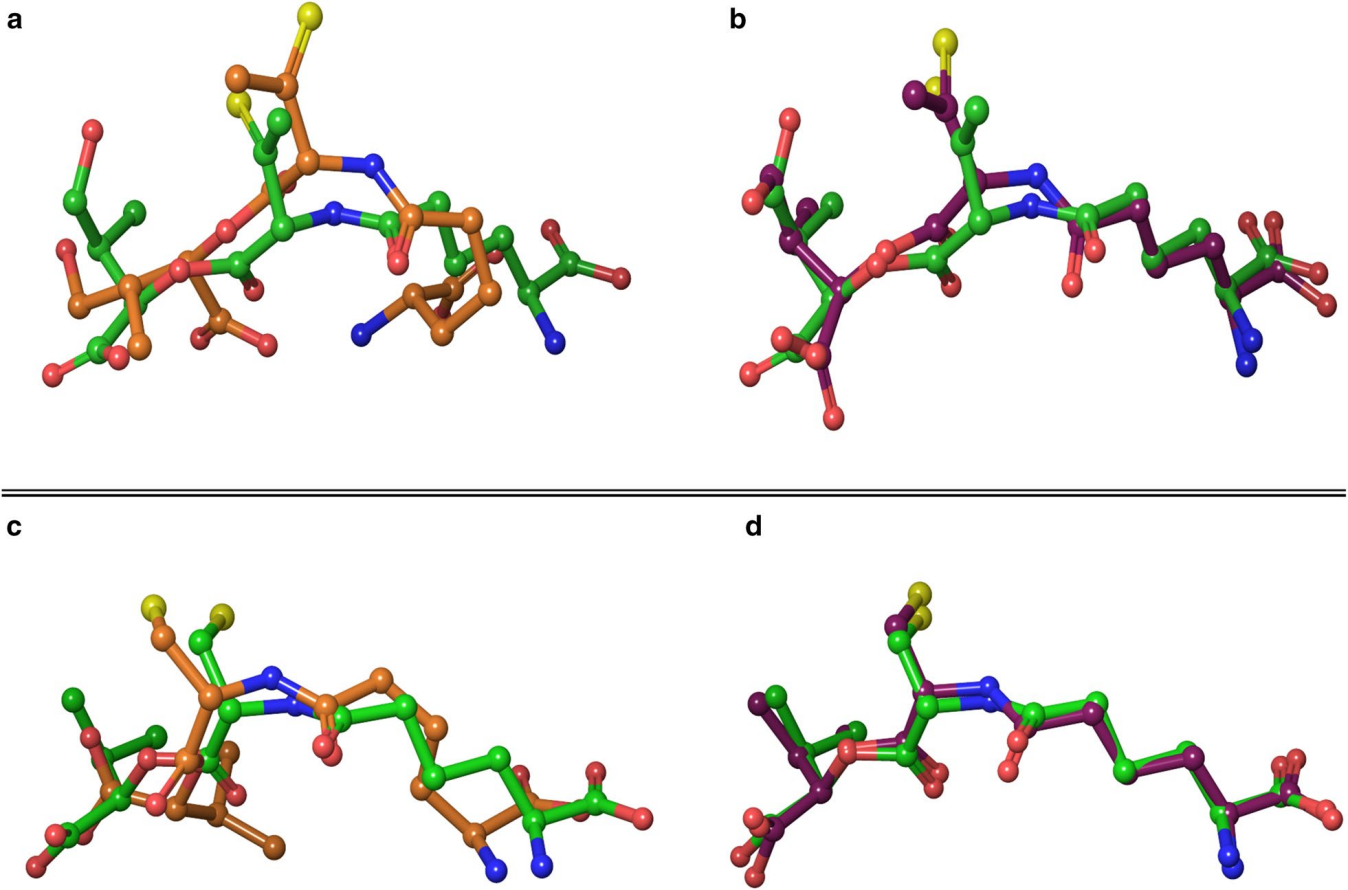
Most similar charged and neutralized conformers superimposed onto the crystal conformation. **a** (PDB ID: W2X): superposition of the most similar charged conformer onto the crystal conformation, **b** (PDB ID: W2X): superposition of the most similar neutralized conformer onto the crystal conformation, **c** (PDB ID: OCV): superposition of the most similar charged conformer onto the crystal conformation, **d** (PDB ID: OCV): superposition of the most similar neutralized conformer onto the crystal conformation. *Green* crystal conformation, *orange* charged conformation, *plum* neutralized conformation

A representative example is depicted in Fig. 3a (PDB ID: W2X), where the presence of a salt bridge between the amino group and the carboxyl group prevented a non-bridged extended conformation from being accepted during the conformational search (crystal conformation: green, most similarly generated conformation: orange, RMSD: 2.63 Å). Neutralization of ligand’s charged groups (to be used as input structure for the conformational search) rendered the electrostatic interaction between the oppositely charged groups less important, allowing the output conformers to adopt a more extended conformation, similar to the crystal pose (Fig. 3b, RMSD: 0.85).

Another example can be seen in Fig. 3c (PDB ID: OCV), where the intramolecular interaction between the thiol group and carboxyl group hinders the molecule from adopting an extended conformation and neutralization of the molecule before conformational search allowed such an extension, which resulted in finding in a highly similar conformation to the crystal pose (Fig. 3d) in the final conformer pool.

A, B (PDB ID): W2X, lower pane; C, D (PDB ID): OCV. Green: crystal pose, orange: charged conformation, plum: neutralized conformation.

Taking a closer look on the ligands highlighted that the improvement was distributed over a large fraction of the ligands in the data set, and these ligands did not share any specific chemical characteristic apart from possessing charged groups in their molecule. This led us to the idea of generating a pool of conformers for each ligand by combining the conformers resulting from both the conformational search with the charged input and the conformational search with the neutralized input. The most similar conformer to the crystal pose was then selected from a combined pool.

A striking improvement was achieved in the averaged best achievable RMSD (sum of minimum RMSDs of all ligands divided by total number of ligands; 809) for all force field-solvent pairs, if the lowest RMSD conformer was selected from the ligand’s combined conformer pool (Additional file 1: Table S5). RMSD values (per compound) decreased 18.0–20.8% when the solvent used was octanol or chloroform. Although the initial objective of this analysis was to examine the outcome with two non-aqueous solvents, a substantial improvement was observed also within the results with water solvation. The fraction of the ligands, which have a conformer below RMSDs of 0.5 and 1.0 Å were determined (Table 2) for both the neutral and the combined dataset.

**Table 2:** Fraction (%) of ligands similar to the reference crystal pose depending on charge and solvent

Force field	Charged (I)		Neutralized (II)		Combined (III)	
	<0.5 Å	<1.0 Å	<0.5 Å	<1.0 Å	<0.5 Å	<1.0 Å
OPLS2005-chloroform	38.6	68.4	43.5	77.1	47.3	79.9
OPLS2005-octanol	40.8	71.1	45.0	77.5	48.9	80.1
OPLS2005-water	39.9	76.9	47.1	82.2	49.6	84.7
MMFFs-chloroform	35.8	67.6	43.4	74.4	46.1	77.9
MMFFs-octanol	40.4	71.1	45.1	79.1	48.2	82.3
MMFFs-water	40.9	77.4	47.3	81.7	50.2	84.3
AMBER-chloroform	36.7	67.5	43.0	75.0	46.2	77.8
AMBER-octanol	38.3	69.2	44.7	76.0	46.6	78.2
AMBER-water	43.1	77.4	45.5	79.0	48.6	81.3
OPLS3-chloroform	39.6	69.3	46.8	74.2	49.9	79.0
OPLS3-octanol	43.8	74.4	50.2	79.0	53.4	82.1
OPLS3-water	47.8	81.6	53.5	81.7	57.4	86.8

Conformer RMSDs were calculated taking crystal pose as reference for; (I) conformational search performed with charged input structures, (II) conformational search performed with neutralized input structures, (III) combined pool of (I) and (II)

**Fig. 4:**
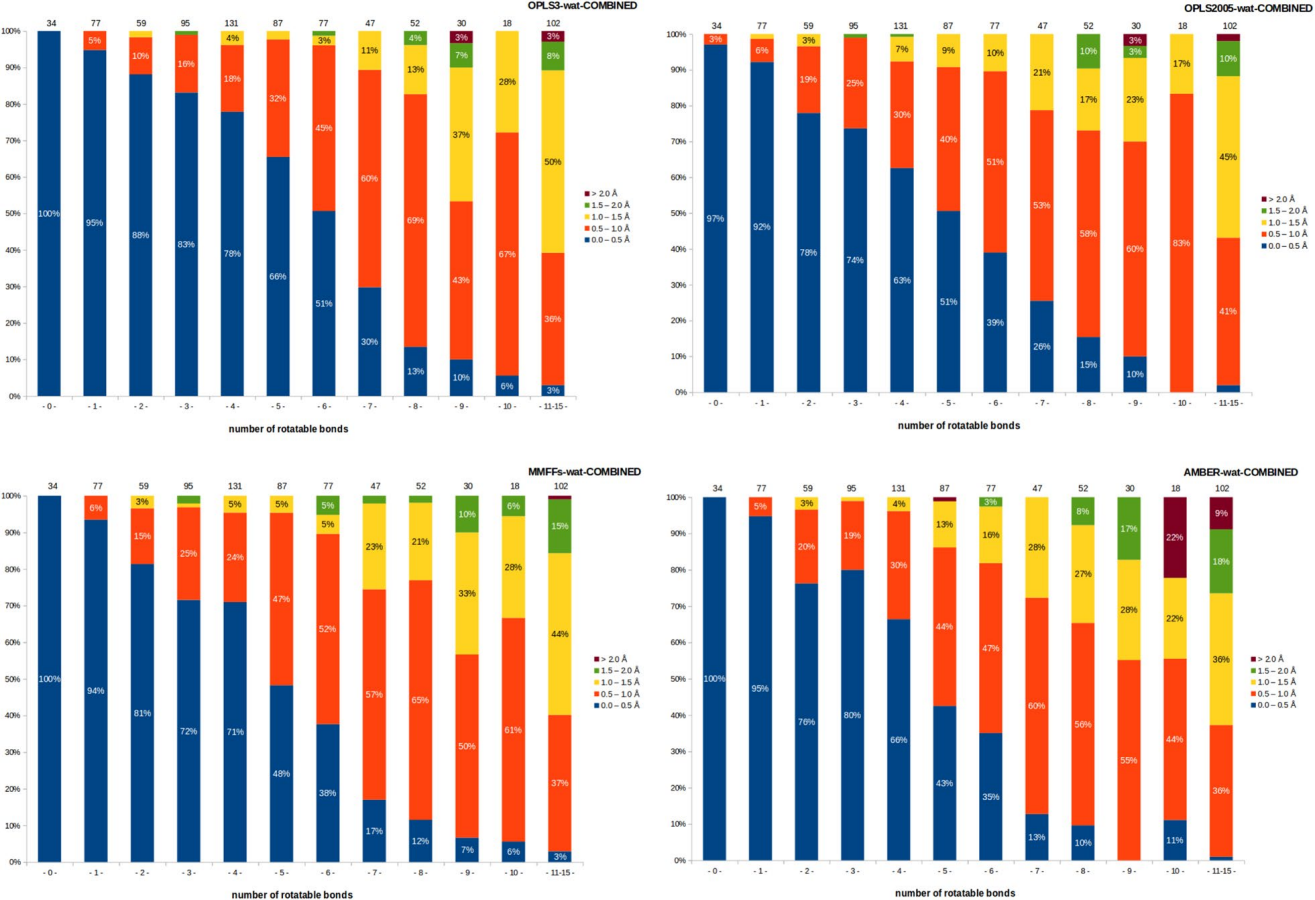
Comparison of results obtained using combined pool of charged and neutralized conformers. For other force field-solvent combinations, the corresponding plots for neutralized conformers and combined pool of conformers (neutralized and charged) can be found in Additional file 1

The combined pool of conformers showed an average improvement of 8.5% for the ligands that rank below 1.0 Å (4.0% minimum for AMBER-water, 11.5% maximum for OPLS2005-chloroform). Figure 4 shows results for the four force fields with implicit solvent (water) and energy window of 5.0 kcal/mol, when the most similar conformer to the crystal pose was selected from the combined pool of charged and neutralized conformers. It can clearly be seen that in all, from the most rigid to most flexible compounds, the fraction of ligands with best achieved RMSDs below 1.0 Å increased considerably.

## Conclusion

Performing conformational searches (and geometry minimizations within) for drug-like ligands in order to prepare a pool of conformers with a high likelihood of containing the bioactive (crystal pose-like) conformation is most feasible using implicit water solvation. In some cases augmenting the final conformer pool with conformers obtained using other solvents or explicit water model can increase the probability of finding the crystal-like conformation. For a vast majority of ligands with up to six rotatable bonds the crystal-like conformers can be accurately generated, however with larger rotatable bond counts the conformers gradually lose their accuracy even with the most advanced force field.

For more flexible compounds or in cases where there is some evidence of increased conformational strain required for binding, enlarging the energy window might be of help, of course at a price of increased computational cost.

The differences among various force fields in terms of the best achievable RMDS are small, nevertheless the most recently released OPLS3 parameter set covering a large variety of dihedral angles proves its expected superiority.

Focused analyses on ligands grouped according to the most relevant physico-chemical descriptors (Lipinski’s rule of 5 and Veber rules) did not show any significant inter-group differences or trends that could be prospectively used when preparing conformer pools for further processing (docking and scoring, or pharmacophore concept).

A higher number of charged groups in the molecule (at pH of the crystallizing solution), capable of forming strong distorting (attractive of repulsive) interactions, is the best indicative of a poor RMSD from the reference crystal conformation. Neutralizing charged groups before conformational searching increases the likelihood of finding extended crystal-like conformations. For achieving the best results, conformers generated based on the neutral species (after re-assigning the assumed protonation state) can be combined into one pool with the charged ones.

The analysis of ligands in NMR models helped to quantify normal conformational fluctuations and showed a trend of increasing inter-model RMSDs with increasing conformation degrees of freedom. This observation is in analogy with the stepwise worsening (0.13 ± 0.02 Å per rotatable bond) of the best achievable RMSD upon increasing #RotB seen with conformers and their crystal pose conformation.

## Additional file

**Additional file 1.** Elimination thresholds for descriptors, PDB IDs of the excluded structures after frequency analysis, PDB IDs of the crystal structures used in this study (complex + ligand), tables and figures that support the article.

## Abbreviations

#HA: number of heavy atoms
#RotB: number of rotatable bonds
3D: three-dimensional
Å: Ångström
CPU: central processing unit
EW: energy window
LogP (o/w): octanol–water partition coefficient
MD: molecular dynamics
MW: molecular weight
NMR: nuclear magnetic resonance
NPT: isothermal–isobaric
PDB: Protein Data Bank
PSA: polar surface area
RMSD: root mean square deviation
SASA: solvent accessible surface area
vdW: van der Waals
X-ray: X-radiation
