# Supplementary material for: Searching for bioactive conformations of drug-like ligands with current force fields: how good are we?
Source: J Cheminform. 2017 May 15;9:29. doi: 10.1186/s13321-017-0216-0 (PMC5432473; doi:10.1186/s13321-017-0216-0)
Supplement: Supplementary file 1 — Additional file 1. Elimination thresholds for descriptors, PDB IDs of the excluded structures after frequency analysis, PDB IDs of the crystal structures used in this study (complex + ligand), tables and figures that support the article. [file 13321_2017_216_MOESM1_ESM.pdf]

| Descriptor | Elimination threshold |
|---|---|
| Total charge | < -3, > +3 |
| Number of rotatable bonds | > 15 |
| Number of heavy atoms | < 10 |
| Molecular weight | < 150, > 650 |

**The PDB IDs of the excluded structures after frequency analysis**

**4 times:** 1PE, ASC, H35, MPO, NZ9, PEP, PHE

**5 times:** 017, BTB

**6 times:** PLM, SKM, TAR, ZEA

**8 times:** FLC

**13 times:** EPE, GSH, TLA

**18 times:** PG4

**20 times:** PGE

**23 times:** MES

**26 times:** CIT

**PDB_ID(Complex)-PDB_ID(Ligand)**

1d2s-DHT, 1adl-ACD, 1aoe-GW3, 1bk0-ACV, 1c1d-PHE, 1c1x-HFA, 1c5n-ESI, 1c5q-ESI, 1cil-ETS, 1df8-BTN, 1dry-AAG, 1dz4-CAM, 1dzk-PRZ, 1e6q-NTZ, 1e6s-GOX, 1e8m-P0H, 1ee2-CHD, 1elu-PDA, 1f8d-9AM, 1f8e-49A, 1fsg-9DG, 1ftk-KAI, 1g67-TZP, 1g6c-IFP, 1g6s-S3P, 1g6s-GPJ, 1gkl-FER, 1gm7-PNN, 1gtz-DHK, 1gvf-PGH, 1gvg-PCX, 1gz8-MBP, 1gzt-FUC, 1h00-FCP, 1h00-FAP, 1h46-RNP, 1hb1-OCV, 1hxk-DMJ, 1hy7-MBS, 1hyo-HBU, 1icm-MYR, 1ie8-KH1, 1ie9-VDX, 1j96-TES, 1ja9-PYQ, 1jd0-AZM, 1jtv-TES, 1jvp-LIG, 1k3l-GTX, 1kb0-PQQ, 1kei-LYS, 1kfc-IPL, 1kjo-THR, 1kjp-GLU, 1kkk-ASP, 1kqw-RTL, 1kvl-THN, 1kvl-CLS, 1kw6-BPY, 1kzk-JE2, 1ltz-HBI, 1m5e-AM1, 1mgo-PFB, 1mqd-SHI, 1mqi-FWD, 1n8v-BDD, 1n8v-BDD, 1n8v-BDD, 1nbu-PH2, 1nlu-TYB, 1nq7-ARL, 1o6f-SIN, 1o98-2PG, 1oaf-ASC, 1obn-ASV, 1odn-APV, 1oew-THR, 1of8-PEP, 1of8-G3P, 1oh0-EQU, 1ohp-ESR, 1oit-HDT, 1ow4-2AN, 1owe-675, 1phb-PFZ, 1phg-MYT, 1q11-TYE, 1q6o-LG6, 1qgu-HCA, 1qiq-ACC, 1qje-IP1, 1qxy-M2C, 1qzy-TDE, 1rb0-HH2, 1re9-DSO, 1sdt-MK1, 1snn-5RP, 1sqn-NDR, 1sv3-ANN, 1t46-STI, 1t7f-DHT, 1tf9-PHI, 1tjp-HPF, 1tjy-PAV, 1tkh-DPN, 1tzc-PA5, 1u0f-G6P, 1u0f-G6Q, 1u3w-FXY, 1uf5-CDT, 1uof-PNN, 1urw-I1P, 1uzw-CDH, 1w04-HCG, 1w3v-MDZ, 1w3x-W2X, 1w6s-PQQ, 1wb4-SXX, 1wb6-VXX, 1wbi-BTN, 1wbj-G3P, 1x9h-F6R, 1x9i-G6Q, 1xbb-STI, 1xg4-ICT, 1xic-XLS, 1xom-CIO, 1xoz-CIA, 1xpc-AIT, 1y2b-DEE, 1y2k-7DE, 1y3v-UIR, 1y5v-NE8, 1y5w-NEZ, 1y9l-UND, 1yvm-TMG, 1zdy-T3A, 1zhx-HC3, 1zhy-CLR, 1zz1-SHH, 2ad6-PQQ, 2afw-AHN, 2aib-ERG, 2al1-PEP, 2al1-2PG, 2ax6-HFT, 2bju-IH4, 2bju-IH4, 2bkx-F6R, 2brc-CT5, 2bu9-HFV, 2bvr-4CP, 2bz6-346, 2c2n-AE4, 2c4j-GSO, 2c92-TP6, 2cle-F6F, 2clk-G3H, 2cll-PLS, 2cll-F9F, 2clo-F19, 2ctc-HFA, 2cxq-S6P, 2d5x-L35, 2d5z-L35, 2d5z-L35, 2de3-OBP, 2dqm-BES, 2ecu-1PG, 2ei0-BP7, 2ei0-BP7, 2ei1-D1N, 2ej0-PMP, 2eu3-FF3, 2f01-BTQ, 2f18-GB1, 2f1a-GB2, 2f1b-GB3, 2f7o-MSN, 2f7p-2SK, 2f7r-SK3, 2fhl-BNI, 2fmg-PHE, 2fmz-DPN, 2gu5-NLP, 2gyi-HYA, 2h7j-H7J, 2hai-PFI, 2hhn-GNQ, 2hzy-DHJ, 2i4u-DJR, 2ica-2IC, 2ikg-BTO, 2ikh-LIT, 2imd-2C2, 2imd-TOH, 2iog-IOG, 2ivi-ACW, 2ivj-BCV, 2izr-BRK, 2j5s-KTA, 2jb4-A14, 2jkb-SKD, 2nmx-M25, 2nnd-PRZ, 2nq6-HM4, 2nq7-HM5, 2nqz-PRF, 2nvd-ITB, 2nwr-PEP, 2o2c-G6Q, 2os1-BB2, 2pdg-47D, 2piy-528, 2piz-606, 2pou-I7A, 2pov-I7B, 2pqz-G0G, 2pr3-237, 2puj-HPZ, 2pw0-TRC, 2pwr-G4G, 2qci-65, 2qp6-MB1, 2qp8-SC7, 2qwc-DAN, 2qyn-NPV, 2qzz-EMF, 2r3f-SC8, 2r3g-SC9, 2r3h-SCE, 2r3r-6SC, 2r43-G3G, 2r8q-IBM, 2rbs-269, 2rct-RTL, 2rdn-1PL, 2rhw-C0E, 2rkv-MPO, 2sim-DAN, 2uy5-H35, 2v00-V15, 2v3i-XX6, 2v3r-XX7, 2v6k-TGG, 2vb8-TLM, 2vbp-VB1, 2vij-C44, 2viz-VG4, 2vj7-VG6, 2vj9-VG7, 2vk2-GZL, 2vro-ETE, 2vuk-P83, 2w09-CM9, 2w0a-CII, 2w0b-CMW, 2w3b-VG9, 2w9h-TOP, 2wbo-ARG, 2wcj-M21, 2wcm-B9M, 2wd4-TBV, 2wec-PP5, 2weg-FBV, 2wej-FB2, 2weo-FBW, 2wer-RDC, 2wf0-ZY0, 2wf1-ZY1, 2wf8-BG6, 2whf-II4, 2whf-II4, 2wsa-646, 2wxd-E18, 2x5w-K2B, 2xf3-J01, 2xf3-J01, 2xir-00J, 2xts-MTE, 2xx8-1NE, 2xxh-1NG, 2xxi-JAC, 2y60-M8F, 2y6d-TQJ, 2yc3-MW5, 2yc5-6BC, 2ydi-YDI, 2yld-ASC, 2z7k-BGU, 2zgb-21U, 2znt-DYH, 2zq2-13U, 2zxg-S23, 2zya-6PG, 2zyd-GLO, 3a40-23R, 3acx-673, 3akn-11D, 3arp-DEQ, 3arx-POY, 3arx-POY, 3az1-DS2, 3az3-DS6, 3b0t-MCZ, 3b34-PHE, 3b7e-ZMR, 3bc9-GLC, 3bex-PAU, 3bhy-7CP, 3blb-SWA, 3bll-BPQ, 3blo-QEI, 3bmx-P4G, 3bmy-CXZ, 3bp1-GUN, 3bqc-EMO, 3bxs-DRS, 3bxs-DRS, 3c2u-B3P, 3cen-FXA, 3cfb-SPB, 3cmj-SRT, 3cse-N22, 3czy-AD8, 3d4y-MVL, 3d51-GOX, 3d52-GHR, 3d78-NBB, 3d7f-YBY, 3d7h-YC2, 3d80-Q22,

3d97-B3P, 3ddf-GB6, 3ddu-552, 3dgq-EAA, 3dha-C6L, 3dhc-CYK, 3dj8-EXY, 3djk-G55, 3dn5-53N, 3dsi-T24, 3dsj-243, 3dsk-T25, 3dux-64U, 3dx2-MZB, 3dx4-GOO, 3eck-XXG, 3ei8-PL5, 3ejp-HN2, 3ejq-HN3, 3ejr-HN4, 3ejs-HN5, 3ejt-HN6, 3eju-HN7, 3eko-PYU, 3eq0-2TS, 3f0u-53R, 3f80-6HN, 3f9m-MRK, 3fed-BIX, 3ffg-FFG, 3fgd-BYA, 3fuc-9DG, 3fun-798, 3fv2-NDZ, 3fvg-MS8, 3fvk-8DX, 3fvn-9DX, 3fvo-8EP, 3g5h-YTT, 3gba-DYH, 3gc5-2MQ, 3ge7-AFQ, 3gen-B43, 3ghc-GHC, 3ghw-GHW, 3gkn-BIH, 3goc-YES, 3gp3-SEP, 3gp6-SDS, 3gp6-SDS, 3gt3-BRV, 3gus-N11, 3gzx-BNL, 3h1x-IMN, 3h61-ENL, 3h8g-BES, 3hcn-CHD, 3hcn-CHD, 3hf5-3ML, 3hp9-CF1, 3huk-J0Z, 3i6c-GIA, 3i6o-GR6, 3ib0-DIF, 3iji-DGL, 3ijl-DGL, 3ip8-B85, 3itu-IBM, 3itv-PSJ, 3iu7-FCD, 3ix3-OHN, 3jum-AOD, 3jyp-QIC, 3k1w-BFX, 3k5x-P8D, 3kbn-GLO, 3kdm-TES, 3kfa-B91, 3kgc-ZK1, 3kgc-LY7, 3ki0-G9D, 3ki1-G9F, 3ki2-G9G, 3ki3-G9H, 3ki5-G9M, 3ki6-G9L, 3ki7-G9I, 3kig-DA4, 3kv2-NNH, 3kx1-KX1, 3l3x-DHT, 3l5e-BDW, 3ljj-10U, 3ljo-11U, 3ly0-LY0, 3m0h-RNS, 3m0j-OAF, 3m0m-AOS, 3m2y-BE0, 3m40-J45, 3m8t-4NZ, 3m98-E02, 3mdu-NGQ, 3mfw-B3U, 3mhw-ABV, 3myq-E27, 3mzc-S6I, 3n0n-P9B, 3n4b-WWZ, 3nhi-EAH, 3nht-U46, 3nnt-DQA, 3ntz-3TZ, 3nu0-3TU, 3nvs-S3P, 3nvs-GPJ, 3nxo-D2B, 3nxx-D2D, 3nz1-3NY, 3nzb-D2N, 3o5l-1PE, 3o9b-K2A, 3o9e-A60, 3o9i-A61, 3ogn-3OG, 3oig-IMJ, 3ok9-G52, 3oq6-PFB, 3ot3-22K, 3ovv-1SB, 3oy0-OY0, 3oyq-OYQ, 3oys-OYS, 3ozm-LY9, 3ozm-DXL, 3p17-99P, 3p6d-ZGB, 3p6e-ZGC, 3p6f-FBZ, 3p6g-IZP, 3p6h-IBP, 3pa3-C70, 3pa4-C72, 3pc3-P1T, 3pcw-FBF, 3pe1-3NG, 3pfb-ZYC, 3pkb-Y16, 3pkc-Y08, 3pkd-Y10, 3pld-F90, 3pmu-F7L, 3pmy-41L, 3poo-S69, 3poz-03P, 3prs-1PE, 3psy-RB9, 3psy-1PE, 3pyx-12T, 3q0w-LL5, 3q6y-S47, 3qaa-G04, 3qih-FG7, 3qih-NI7, 3qke-GCO, 3ql0-FOL, 3qp0-NI8, 3qp1-HL6, 3qp4-HL0, 3qrs-NK8, 3qyk-IE2, 3r16-5UN, 3r92-06J, 3rbu-G88, 3rly-S29, 3rm0-S54, 3rm6-18Z, 3rml-M31, 3rmm-M32, 3ryj-RYJ, 3ryv-RYV, 3ryx-RYX, 3ryy-RYY, 3s0e-EOL, 3s2j-L3A, 3s2l-LDE, 3s2m-P5D, 3s2n-P4D, 3s72-EVE, 3s74-03T, 3s8x-BCN, 3sa7-55A, 3sa8-K83, 3sab-F78, 3sac-AF8, 3san-ZMR, 3sbf-D8T, 3sbi-E90, 3sha-P97, 3sm0-AEK, 3sqy-Q11, 3szl-H6P, 3t44-IGP, 3t4s-H35, 3t5f-M34, 3t6i-Z82, 3tao-PGH, 3tcy-PHE, 3th9-9Y9, 3ti8-LNV, 3tkd-CYZ, 3tki-S25, 3tog-79, 3tpp-5HA, 3u98-BJA, 3u9q-DKA, 3u9w-28P, 3ucc-2PG, 3ucd-PEP, 3up0-D7S, 3up3-XCA, 3upe-0CA, 3uqi-MPO, 3urk-0CG, 3uv3-0CM, 3uv7-0CN, 3v7x-D7A, 3v9v-21L, 3veu-0GO, 3vf3-0GS, 3vgn-FNN, 3vhd-VHE, 3vhe-42Q, 3vhv-LD1, 3vif-LGC, 3vig-NOJ, 3vk5-FPQ, 3vmk-IPM, 3vo3-0KF, 3vq8-BCU, 3vri-1KX, 3vtc-TK3, 3vxi-ASC, 3vxj-3DM, 3wfg-WFG, 3wvs-RRM, 3wx9-3EE, 3zcl-5TF, 3zkq-B3P, 3zku-HCV, 3zky-WT4, 3zou-6H6, 3zou-GPP, 3zxh-E41, 3zzs-TRP, 4a39-GEM, 4a7g-12I, 4a7u-ALE, 4a7v-LDP, 4a83-DXC, 4a85-H35, 4a86-H35, 4agm-P86, 4avs-N7P, 4awq-592, 4ayq-MVL, 4ayr-IFL, 4ayr-IFL, 4b11-7I1, 4b13-X25, 4b14-4XB, 4b70-WM9, 4b72-2FB, 4b78-KGG, 4bb3-KKA, 4bcd-TDV, 4bcw-TU0, 4bgu-1PE, 4bmn-TAM, 4bue-JQF, 4bw1-S5B, 4c1c-MCO, 4c1e-MCO, 4c38-VUP, 4c72-TLG, 4cae-3F3, 4cgl-A6K, 4cgo-6KV, 4cgo-6KV, 4cpf-LH3, 4cyo-UEK, 4dgr-3LV, 4di9-0GY, 4djr-0KJ, 4djx-0KQ, 4do4-DJN, 4do5-DGJ, 4don-3PF, 4drm-0MC, 4dtz-LDP, 4dz7-D02, 4dz9-ID4, 4e3d-GTQ, 4e3f-GRE, 4e4a-JKE, 4egh-0OY, 4ehv-0SJ, 4ehv-0SJ, 4ek5-03K, 4ek6-10K, 4ew2-DXY, 4ey2-0RM, 4eyb-0WO, 4fkg-4CK, 4fkl-CK2, 4fko-20K, 4flg-ILE, 4fli-Y16, 4flk-Y10, 4fll-YZ6, 4fm7-0UP, 4fmx-CNL, 4fps-0UX, 4g1q-T27, 4g1x-TQU, 4g1x-TQU, 4g44-TZM, 4g45-MQN, 4g46-TTY, 4g47-TZF, 4g48-PZB, 4g8b-HL6, 4g9e-C4L, 4gcj-X64, 4gd9-BTN, 4ger-LYS, 4gg9-0WW, 4ghe-4NC, 4ghg-DHY, 4gkt-0O1, 4gv1-0XZ, 4h3j-10W, 4h4d-10E, 4h6b-10X, 4h6b-10Y, 4hct-18R, 4hdb-G52, 4hg9-G3P, 4hjl-15O, 4hlc-T05, 4hry-TAM, 4ht2-V50, 4ht2-V50,

4huo-RS8, 4hxn-1A7, 4hy4-1BG, 4hyi-1AO, 4hzm-1BW, 4hzz-G39, 4i00-ZMR, 4i3b-BLA, 4i3g-MPO, 4i7t-1DW, 4ibg-1D6, 4ibj-1D9, 4igt-3ZA, 4ikt-TFV, 4iku-SHX, 4ilx-1EZ, 4inw-1EY, 4ips-1G4, 4ipw-1G7, 4itu-1HS, 4ivt-VTI, 4ixe-IXE, 4j5h-1K4, 4j7n-9MG, 4ja8-1K9, 4jej-1GP, 4jew-P00, 4jey-PLP, 4jfj-1KU, 4jfk-JFK, 4jfl-1KY, 4jfm-1KZ, 4jh7-1KM, 4jht-8XQ, 4jmb-1LW, 4jpt-Q24, 4jq1-NPS, 4jq4-IMN, 4jqa-ID8, 4jtq-FLP, 4jtr-IZP, 4jtr-IBP, 4jv8-1M1, 4jze-1NK, 4k60-1P8, 4k9g-1Q2, 4kae-TGG, 4kap-1QV, 4keb-1QZ, 4kfg-DOO, 4kfn-1QR, 4kfo-1QS, 4klv-KLV, 4kmv-T6C, 4knn-E1F, 4kor-4KR, 4kot-CE3, 4kp6-1S1, 4ks7-X4Z, 4ktj-KTJ, 4ktk-KTK, 4kz4-4A1, 4kz5-1U3, 4kz7-1U5, 4kza-NZ9, 4kza-NZ9, 4kza-NZ9, 4kza-NZ9, 4kzb-NZ2, 4kzb-NZ3, 4kzb-NZ2, 4kzb-NZ2, 4l6b-1VS, 4l7g-1W0, 4l9p-FII, 4lge-1Y0, 4lhi-WWL, 4lls-IPE, 4llt-IPE, 4lrq-MPO, 4lva-20M, 4lvf-20P, 4lwe-FJ2, 4lwg-FJ4, 4lz5-1YV, 4m14-QWS, 4m14-QWS, 4m5r-MSR, 4mc1-526, 4mc2-525, 4mc3-28U, 4mc6-23K, 4mc9-23L, 4mmm-BP7, 4mnc-173, 4mrh-2CQ, 4mry-CEI, 4mrz-2ZV, 4myd-164, 4n6k-X3X, 4nfn-2KC, 4nn3-ORO, 4nog-PLP, 4ntk-ZSP, 4o10-2QF, 4o71-CPB, 4oba-2TW, 4og6-2S9, 4ohu-2TK, 4otq-2V2, 4p4c-25Q, 4p5q-Q0B, 4p7x-CXS, 4pdy-HIS, 4psb-GA3, 4pyq-2X1, 4q4o-2YM, 4q4r-SQO, 4qf7-C0R, 4qte-390, 4tvt-ASC, 4u21-FWF, 4ua4-3C7, 7atj-FER

**Table S1.** Variation of molecular weight (MW) and number of heavy atoms (#HA) for different ranges of rotatable bond counts.

| MW | % in #ROTB 0-3 | MW | % in #ROTB 4-6 | MW | % in #ROTB 7-10 | MW | % in #ROTB 10-15 |
|---|---|---|---|---|---|---|---|
| **150 – 250** | 67 | 150 – 250 | 36 | 150 – 250 | 16 | 150 – 250 | 20 |
| **250 – 350** | 23 | 250 – 350 | 33 | 250 – 350 | 27 | 250 – 350 | 29 |
| **350 – 450** | 10 | 350 – 450 | 25 | 350 – 450 | 27 | 350 – 450 | 14 |
| **450 – 650** | 0 | 450 – 650 | 6 | 450 – 650 | 30 | 450 – 650 | 37 |
| | | | | | | | |
| **#HA** | **% in #ROTB 0-3** | **#HA** | **% in #ROTB 4-6** | **#HA** | **% in #ROTB 7-10** | **#HA** | **% in #ROTB 10-15** |
| **10 – 15** | 57 | 10 – 15 | 28 | 10 – 15 | 11 | 10 – 15 | 11 |
| **15 – 20** | 21 | 15 – 20 | 23 | 15 – 20 | 15 | 15 – 20 | 31 |
| **20 – 25** | 16 | 20 – 25 | 23 | 20 – 25 | 22 | 20 – 25 | 11 |
| **25 – 30** | 5 | 25 – 30 | 18 | 25 – 30 | 18 | 25 – 30 | 6 |
| **>30** | 1 | >30 | 8 | >30 | 34 | >30 | 40 |

**Table S2.** Variation of number of rotatable bonds (#ROTB) and number of heavy atoms (#HA) for different ranges of molecular weight (MW).

| #HA | % in MW 150-250 | #HA | % in MW 250-350 | #HA | % in MW 350-450 | #HA | % in MW 450-650 |
|---|---|---|---|---|---|---|---|
| **10 – 15** | 78 | 10 – 15 | 4 | 10 – 15 | 0 | 10 – 15 | 0 |
| **15 – 20** | 22 | 15 – 20 | 40 | 15 – 20 | 1 | 15 – 20 | 0 |
| **20 – 25** | 0 | 20 – 25 | 56 | 20 – 25 | 26 | 20 – 25 | 1 |
| **25 – 30** | 0 | 25 – 30 | 0 | 25 – 30 | 62 | 25 – 30 | 3 |
| **>30** | 0 | >30 | 0 | >30 | 11 | >30 | 96 |

| #ROTB | % in MW 150-250 | #ROTB | % in MW 250-350 | #ROTB | % in MW 350-450 | #ROTB | % in MW 450-650 |
|---|---|---|---|---|---|---|---|
| **≤ 4** | 76 | ≤ 4 | 47 | ≤ 4 | 34 | ≤ 4 | 2 |
| **≤ 7** | 17 | ≤ 7 | 33 | ≤ 7 | 37 | ≤ 7 | 23 |
| **≤ 10** | 5 | ≤ 10 | 11 | ≤ 10 | 15 | ≤ 10 | 33 |
| **≤ 15** | 3 | ≤ 15 | 10 | ≤ 15 | 14 | ≤ 15 | 43 |

**Table S3.** Increase of RMSD according to the number of charged groups on ligands (CHARGED INPUT).

| #charged groups | total#comps | RMSD per comp. OPLS2005-chloroform | RMSD per comp. MMFFs-chloroform | RMSD per comp. OPLS3-chloroform | RMSD per comp. AMBER-chloroform |
|---|---|---|---|---|---|
| **1** | 328 | 0.79 | 0.78 | 0.77 | 0.78 |
| **2** | 112 | 1.03 | 1.08 | 0.97 | 1.03 |
| **3** | 62 | 1.64 | 1.64 | 1.49 | 1.63 |
| **4-5** | 15 | 1.51 | 1.64 | 1.42 | 1.56 |
| | | | | | |
| **#charged groups** | **total#comps** | **RMSD per comp. OPLS2005-octanol** | **RMSD per comp. MMFFs-octanol** | **RMSD per comp. OPLS3-octanol** | **RMSD per comp. AMBER-octanol** |
| **1** | 328 | 0.75 | 0.73 | 0.72 | 0.79 |
| **2** | 112 | 1.00 | 0.97 | 0.88 | 1.05 |
| **3** | 62 | 1.52 | 1.55 | 1.43 | 1.57 |
| **4-5** | 15 | 1.50 | 1.57 | 1.38 | 1.51 |
| | | | | | |
| **#charged groups** | **total#comps** | **RMSD per comp. OPLS2005-water** | **RMSD per comp. MMFFs-water** | **RMSD per comp. OPLS3-water** | **RMSD per comp. AMBER-water** |
| **1** | 328 | 0.64 | 0.62 | 0.59 | 0.65 |
| **2** | 112 | 0.79 | 0.79 | 0.69 | 0.72 |
| **3** | 62 | 1.29 | 1.28 | 0.97 | 0.87 |
| **4-5** | 15 | 1.40 | 1.20 | 1.11 | 1.15 |

**Table S4.** Increase of RMSD according to the number of charged groups on ligands (NEUTRALIZED INPUT).

| #charged groups | total#comps | RMSD per comp. OPLS2005-chloroform | RMSD per comp. MMFFs-chloroform | RMSD per comp. OPLS3-chloroform | RMSD per comp. AMBER-chloroform |
|---|---|---|---|---|---|
| **1** | 328 | 0.66 | 0.66 | 0.68 | 0.69 |
| **2** | 112 | 0.82 | 0.83 | 0.83 | 0.83 |
| **3** | 62 | 1.15 | 1.12 | 1.01 | 1.01 |
| **4-5** | 15 | 1.25 | 1.09 | 1.25 | 1.12 |
| | | | | | |
| **#charged groups** | **total#comps** | **RMSD per comp. OPLS2005-octanol** | **RMSD per comp. MMFFs-octanol** | **RMSD per comp. OPLS3-octanol** | **RMSD per comp. AMBER-octanol** |
| **1** | 328 | 0.64 | 0.63 | 0.65 | 0.67 |
| **2** | 112 | 0.78 | 0.78 | 0.76 | 0.89 |
| **3** | 62 | 0.94 | 1.00 | 1.01 | 0.87 |
| **4-5** | 15 | 1.13 | 1.04 | 1.13 | 0.98 |
| | | | | | |
| **#charged groups** | **total#comps** | **RMSD per comp. OPLS2005-water** | **RMSD per comp. MMFFs-water** | **RMSD per comp. OPLS3-water** | **RMSD per comp. AMBER-water** |
| **1** | 328 | 0.59 | 0.56 | 0.58 | 0.63 |
| **2** | 112 | 0.73 | 0.70 | 0.73 | 0.79 |
| **3** | 62 | 0.71 | 0.78 | 0.74 | 0.78 |
| **4-5** | 15 | 0.91 | 0.77 | 0.89 | 0.85 |

**Table S5.** Sum of minimum RMSDs (Å) of all ligands divided by total number of ligands (809) for all force field-solvent pairs (2nd, 3rd, 4th columns), improvement of RMSD in Ångström (5th column), improvement of RMSD in percentage (6th column).

| Force field | Charged (Å) | Neutralized (Å) | Combined (Å) | Difference (Å) (Charged−Combined) | % improvement (Charged−Combined) |
|---|---|---|---|---|---|
| **OPLS2005-chloroform** | 0.841 | 0.719 | 0.672 | 0.168 | 20.0 |
| **OPLS2005-octanol** | 0.803 | 0.683 | 0.640 | 0.163 | 20.3 |
| **OPLS2005-water** | 0.717 | 0.630 | 0.585 | 0.132 | 18.4 |
| **MMFFs-chloroform** | 0.858 | 0.727 | 0.681 | 0.177 | 20.7 |
| **MMFFs-octanol** | 0.807 | 0.686 | 0.639 | 0.168 | 20.8 |
| **MMFFs-water** | 0.709 | 0.623 | 0.583 | 0.127 | 17.9 |
| **AMBER-chloroform** | 0.859 | 0.737 | 0.687 | 0.172 | 20.0 |
| **AMBER-octanol** | 0.862 | 0.730 | 0.690 | 0.173 | 20.0 |
| **AMBER-water** | 0.698 | 0.688 | 0.629 | 0.069 | 9.8 |
| **OPLS3-chloroform** | 0.818 | 0.721 | 0.662 | 0.156 | 19.1 |
| **OPLS3-octanol** | 0.767 | 0.680 | 0.629 | 0.138 | 18.0 |
| **OPLS3-water** | 0.636 | 0.608 | 0.543 | 0.093 | 14.6 |

**Table S6.** Results of the statistical analysis of RMSD increase per rotatable-bond for all force field-solvent combinations.

| **#ROTB –>** | **0** | | **1** | | **2** | | **3** | | **4** | | **5** | | **6** | |
|---|---|---|---|---|---|---|---|---|---|---|---|---|---|---|
| **OPLS2005-chloroform** | 4.86 | 0.14 | 21.66 | 0.14 | 23.94 | 0.14 | 50.91 | 0.13 | 83.92 | 0.13 | 68.04 | 0.13 | 72.09 | 0.13 |
| **OPLS2005-octanol** | 4.98 | 0.15 | 21.58 | 0.14 | 24.39 | 0.14 | 48.46 | 0.13 | 77.68 | 0.12 | 62.21 | 0.12 | 70.78 | 0.13 |
| **OPLS2005-water** | 4.80 | 0.14 | 21.94 | 0.14 | 27.20 | 0.15 | 46.79 | 0.12 | 79.37 | 0.12 | 58.34 | 0.11 | 56.40 | 0.10 |
| **MMFFs-chloroform** | 4.00 | 0.12 | 21.11 | 0.14 | 24.68 | 0.14 | 50.69 | 0.13 | 82.31 | 0.13 | 68.43 | 0.13 | 75.79 | 0.14 |
| **MMFFs-octanol** | 3.94 | 0.12 | 20.20 | 0.13 | 23.10 | 0.13 | 47.62 | 0.13 | 74.27 | 0.11 | 62.60 | 0.12 | 72.55 | 0.13 |
| **MMFFs-water** | 4.39 | 0.13 | 20.71 | 0.13 | 23.50 | 0.13 | 47.75 | 0.13 | 71.58 | 0.11 | 56.17 | 0.11 | 60.13 | 0.11 |
| **OPLS3-chloroform** | 4.45 | 0.13 | 21.06 | 0.14 | 22.12 | 0.12 | 50.12 | 0.13 | 78.69 | 0.12 | 62.77 | 0.12 | 69.53 | 0.13 |
| **OPLS3-octanol** | 4.44 | 0.13 | 20.31 | 0.13 | 19.07 | 0.11 | 46.65 | 0.12 | 71.14 | 0.11 | 55.39 | 0.11 | 62.76 | 0.12 |
| **OPLS3-water** | 4.67 | 0.14 | 20.15 | 0.13 | 20.93 | 0.12 | 39.60 | 0.10 | 61.60 | 0.09 | 49.49 | 0.09 | 51.68 | 0.10 |
| **OPLS3-gas** | 4.54 | 0.13 | 20.86 | 0.14 | 20.79 | 0.12 | 54.53 | 0.14 | 86.43 | 0.13 | 75.85 | 0.15 | 82.60 | 0.15 |
| **AMBER-chloroform** | 4.15 | 0.12 | 21.09 | 0.14 | 23.19 | 0.13 | 50.02 | 0.13 | 85.88 | 0.13 | 65.22 | 0.12 | 74.12 | 0.14 |
| **AMBER-octanol** | 4.12 | 0.12 | 20.76 | 0.13 | 23.45 | 0.13 | 46.86 | 0.12 | 78.12 | 0.12 | 65.89 | 0.13 | 76.20 | 0.14 |
| **AMBER-water** | 3.97 | 0.12 | 20.45 | 0.13 | 23.84 | 0.13 | 39.76 | 0.10 | 68.43 | 0.10 | 59.70 | 0.11 | 56.03 | 0.10 |
| **Explicit-water** | 6.93 | 0.20 | 27.04 | 0.18 | 22.90 | 0.13 | 47.85 | 0.13 | 85.36 | 0.13 | 64.27 | 0.12 | 68.97 | 0.13 |
| **BABEL-MMFFs** | 25.05 | 0.74 | 80.34 | 0.52 | 46.73 | 0.26 | 71.43 | 0.19 | 130.47 | 0.20 | 100.39 | 0.19 | 97.45 | 0.18 |
| | | | | | | | | | | | | | | |
| **# Compounds** | 34 | | 77 | | 59 | | 95 | | 131 | | 87 | | 77 | |

## Table S6. (Cont'd)

| #ROTB –> | 7 | | 8 | | 9 | | 10 | | >11 | | RMSD-sum | |
|---|---|---|---|---|---|---|---|---|---|---|---|---|
| OPLS2005-chloroform | 54.53 | 0.15 | 66.58 | 0.14 | 39.47 | 0.13 | 28.65 | 0.14 | 165.56 | 0.14 | 680.21 | 0.84 |
| OPLS2005-octanol | 50.53 | 0.13 | 59.90 | 0.13 | 40.72 | 0.14 | 23.60 | 0.12 | 164.50 | 0.13 | 649.33 | 0.80 |
| OPLS2005-water | 41.29 | 0.11 | 47.97 | 0.10 | 33.28 | 0.11 | 20.98 | 0.11 | 141.32 | 0.12 | 579.68 | 0.72 |
| MMFFs-chloroform | 55.28 | 0.15 | 69.46 | 0.15 | 40.61 | 0.14 | 27.19 | 0.14 | 174.67 | 0.14 | 694.22 | 0.86 |
| MMFFs-octanol | 51.06 | 0.14 | 61.89 | 0.13 | 39.73 | 0.13 | 25.38 | 0.13 | 170.23 | 0.14 | 652.57 | 0.81 |
| MMFFs-water | 41.13 | 0.11 | 48.27 | 0.10 | 32.32 | 0.11 | 22.31 | 0.11 | 145.60 | 0.12 | 573.86 | 0.71 |
| OPLS3-chloroform | 52.25 | 0.14 | 63.11 | 0.13 | 41.72 | 0.14 | 28.23 | 0.14 | 168.00 | 0.14 | 662.05 | 0.82 |
| OPLS3-octanol | 49.70 | 0.13 | 59.40 | 0.13 | 40.34 | 0.13 | 24.62 | 0.12 | 166.42 | 0.14 | 620.24 | 0.77 |
| OPLS3-water | 37.43 | 0.10 | 47.21 | 0.10 | 33.09 | 0.11 | 17.46 | 0.09 | 131.16 | 0.11 | 514.47 | 0.64 |
| OPLS3-gas | 61.57 | 0.16 | 73.37 | 0.16 | 49.27 | 0.16 | 29.52 | 0.15 | 195.27 | 0.16 | 754.60 | 0.93 |
| AMBER-chloroform | 55.37 | 0.15 | 63.83 | 0.14 | 43.32 | 0.14 | 27.32 | 0.14 | 181.32 | 0.15 | 694.83 | 0.86 |
| AMBER-octanol | 54.53 | 0.15 | 63.69 | 0.14 | 45.65 | 0.15 | 27.29 | 0.14 | 190.90 | 0.16 | 697.46 | 0.86 |
| AMBER-water | 45.39 | 0.12 | 50.99 | 0.11 | 36.11 | 0.12 | 22.79 | 0.12 | 137.37 | 0.11 | 564.83 | 0.70 |
| Explicit-water | 46.83 | 0.12 | 61.38 | 0.13 | 37.03 | 0.12 | 22.98 | 0.12 | 154.75 | 0.13 | 646.29 | 0.80 |
| BABEL-MMFFs | 66.88 | 0.18 | 72.76 | 0.16 | 48.57 | 0.16 | 34.15 | 0.17 | 180.05 | 0.15 | 954.27 | 1.18 |
| # Compounds | 47 | | 52 | | 30 | | 18 | | 102 | | 809 | |

**Table S7.** Fraction of the ligands (%) that have the bioactive pose within the stated RMSD ranges (first column) from the global minimum structure.

| RMSD | AMBER Chloroform | AMBER Octanol | AMBER Water | MMFFs Chloroform | MMFFs Octanol | MMFFs Water | OPLS3 Chloroform | OPLS3 Octanol | OPLS3 Water | OPLS2005 Chloroform | OPLS2005 Octanol | OPLS2005 Water |
|---|---|---|---|---|---|---|---|---|---|---|---|---|
| 0.0 – 0.5 | 15.8 | 15.7 | 17.4 | 16.4 | 15.3 | 16.2 | 13.7 | 14.7 | 17.6 | 15.1 | 15.1 | 16.8 |
| 0.5 – 1.0 | 14.7 | 14.7 | 15.3 | 14.7 | 15.3 | 19.4 | 17.3 | 16.9 | 17.6 | 16.3 | 15.1 | 17.9 |
| 1.0 – 1.5 | 20.6 | 21.1 | 22.0 | 22.2 | 21.0 | 21.1 | 21.4 | 21.1 | 21.9 | 23.0 | 22.4 | 23.2 |
| 1.5 – 2.0 | 17.6 | 17.7 | 15.8 | 18.0 | 18.8 | 16.7 | 19.7 | 17.8 | 16.4 | 17.6 | 19.3 | 15.5 |
| 2.0 – 3.0 | 19.8 | 18.9 | 17.1 | 20.5 | 20.1 | 18.5 | 18.7 | 17.2 | 16.4 | 17.7 | 18.0 | 17.4 |
| > 3.0 | 11.5 | 11.9 | 12.4 | 8.0 | 9.4 | 8.0 | 9.3 | 12.2 | 10.1 | 10.4 | 10.1 | 9.1 |

Best achieved RMSDs (most similar conformer to the crystal-pose) for ligands grouped by various descriptors (OPLS3 force field).

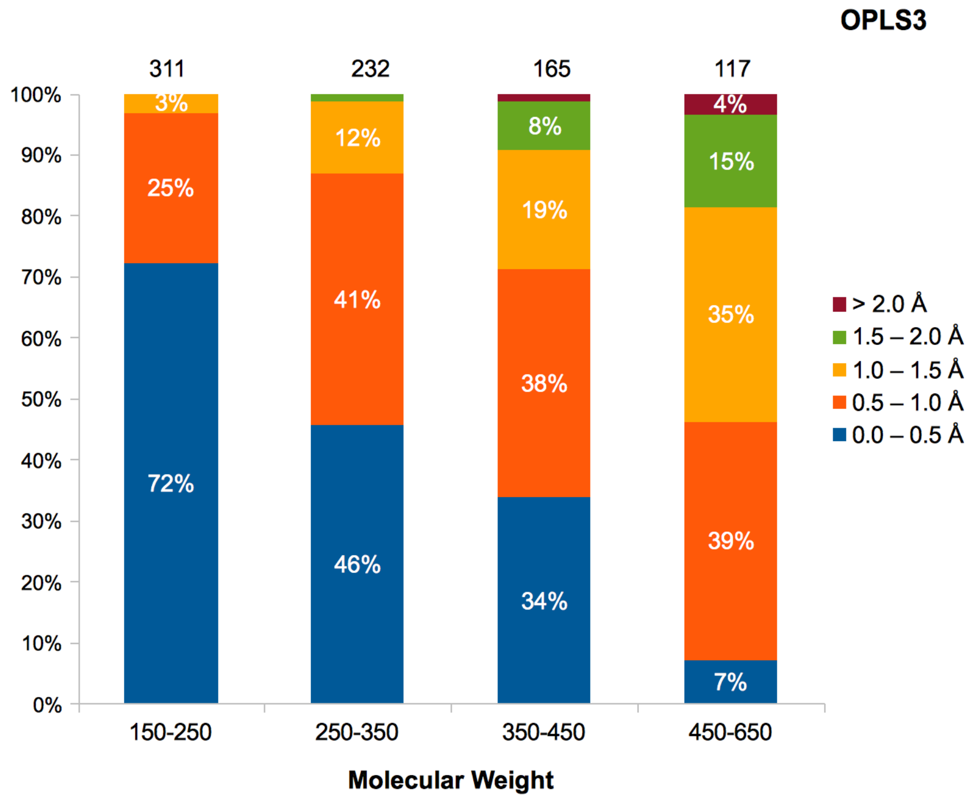


**Figure S1**

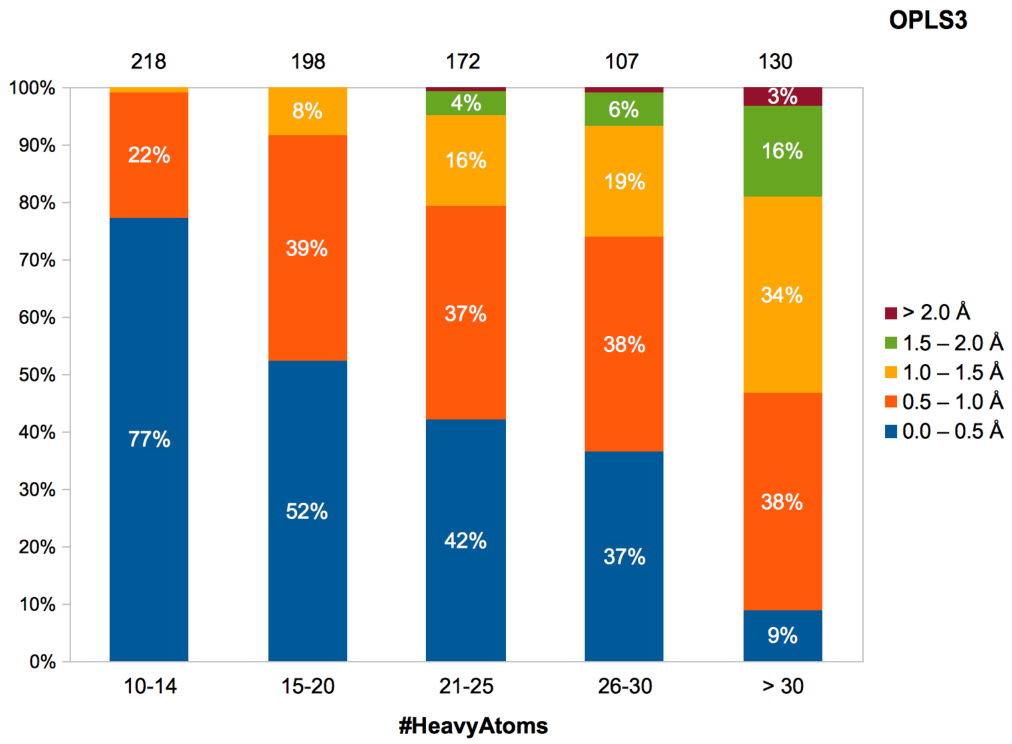


**Figure S2**

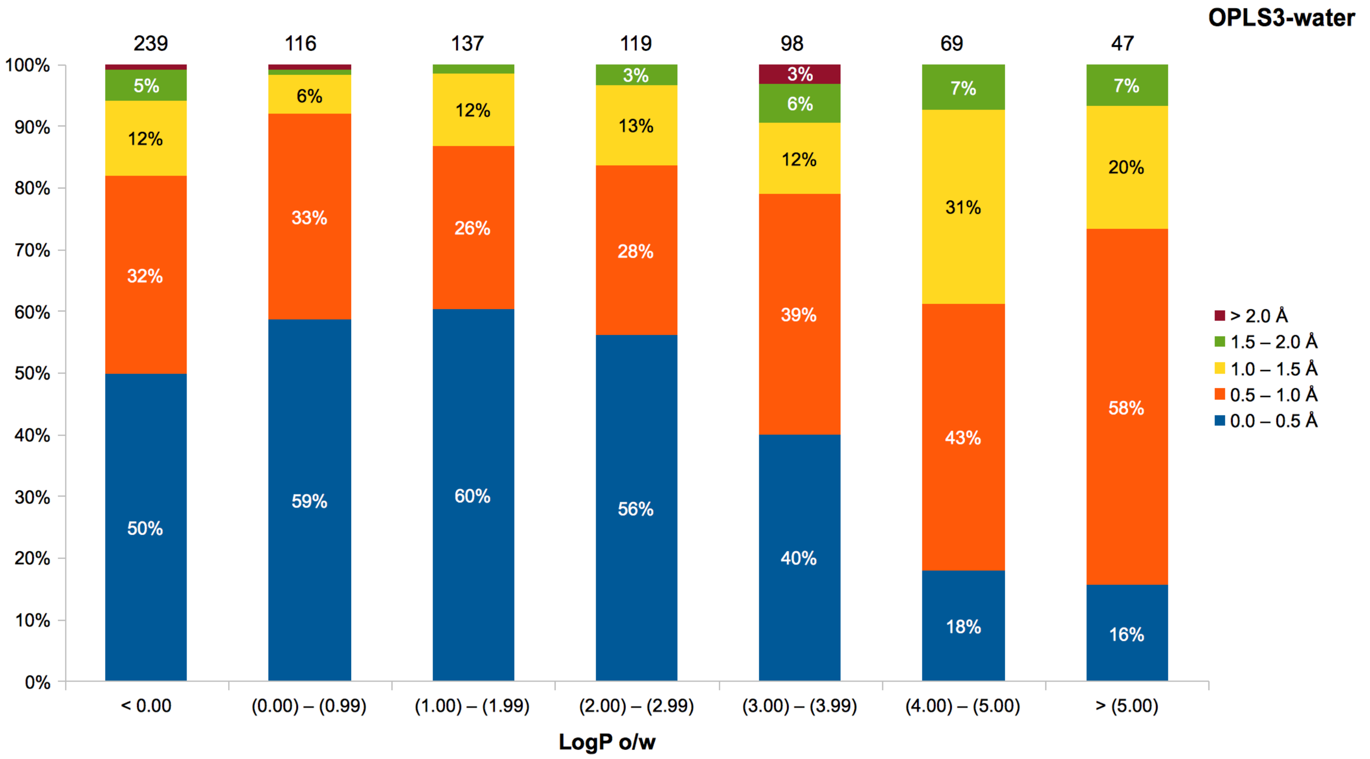


**Figure S3**

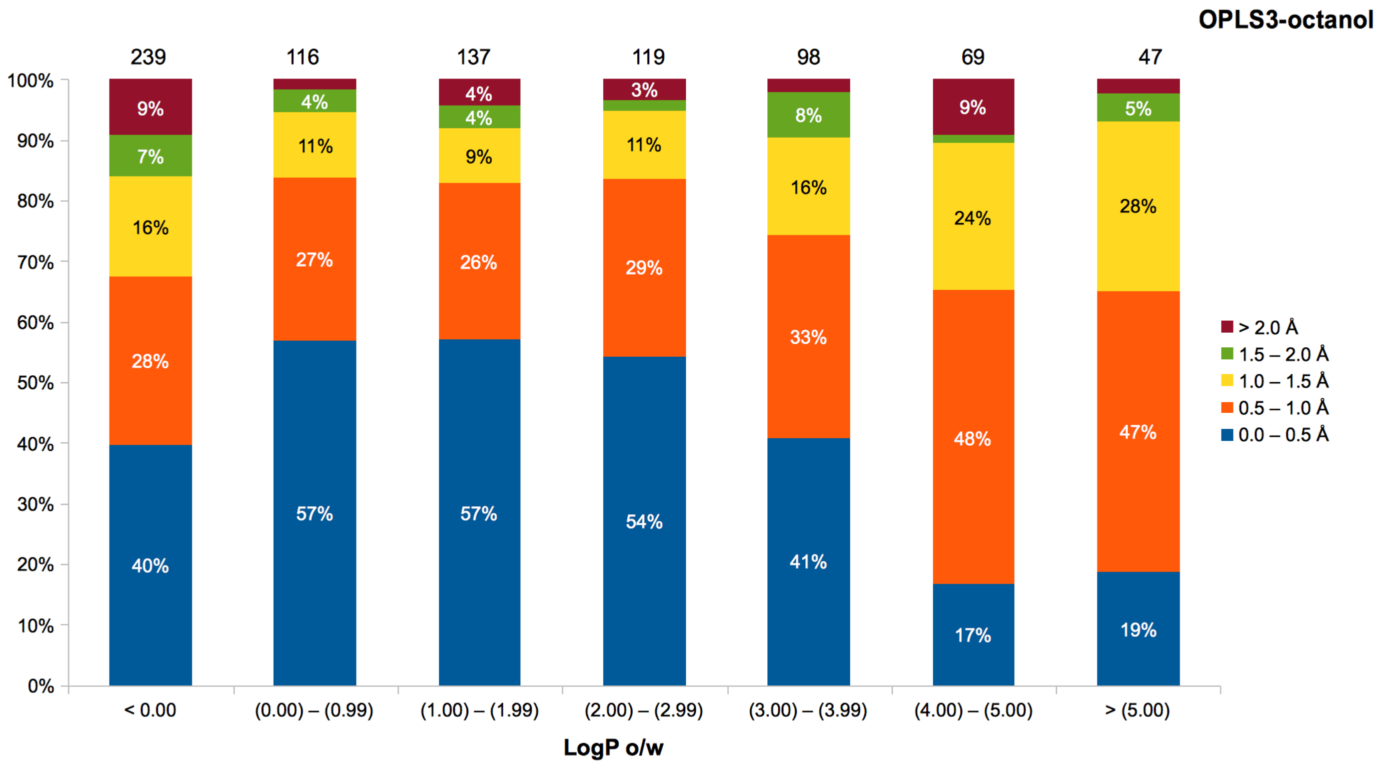


**Figure S4**

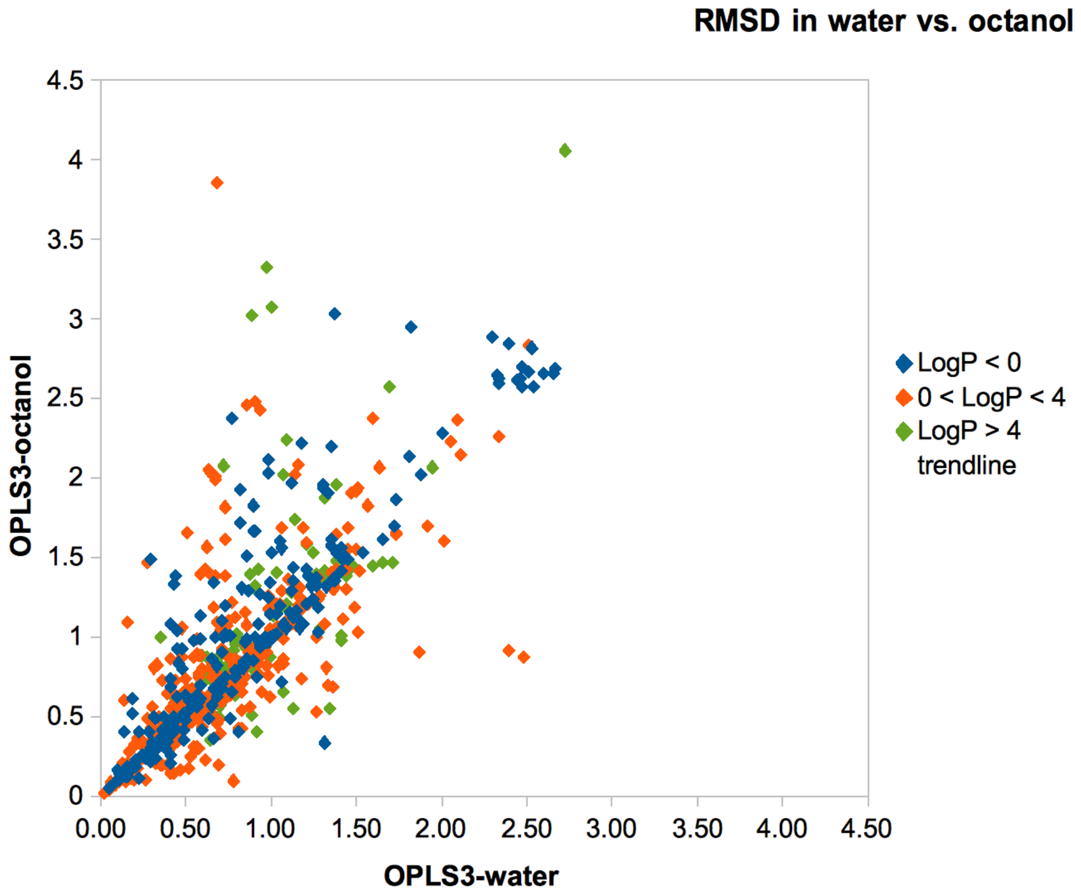


Figure S5

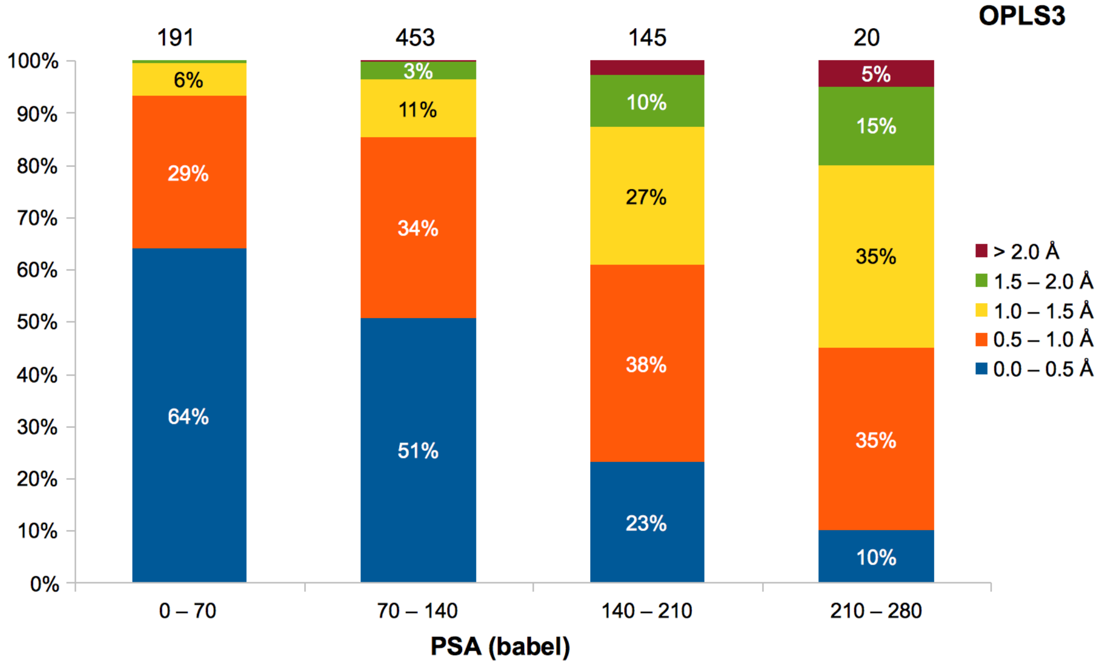


Figure S6

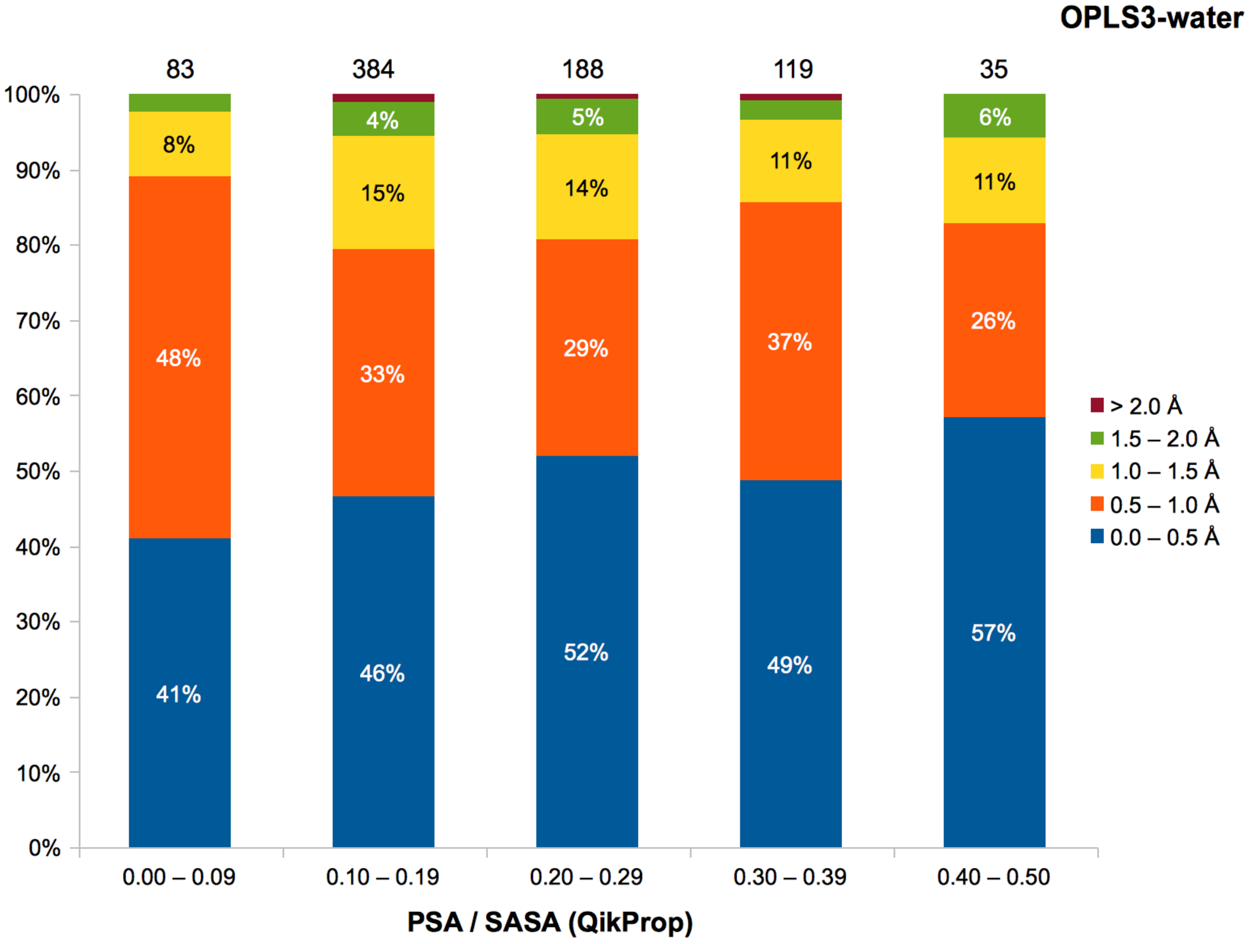


**Figure S7**

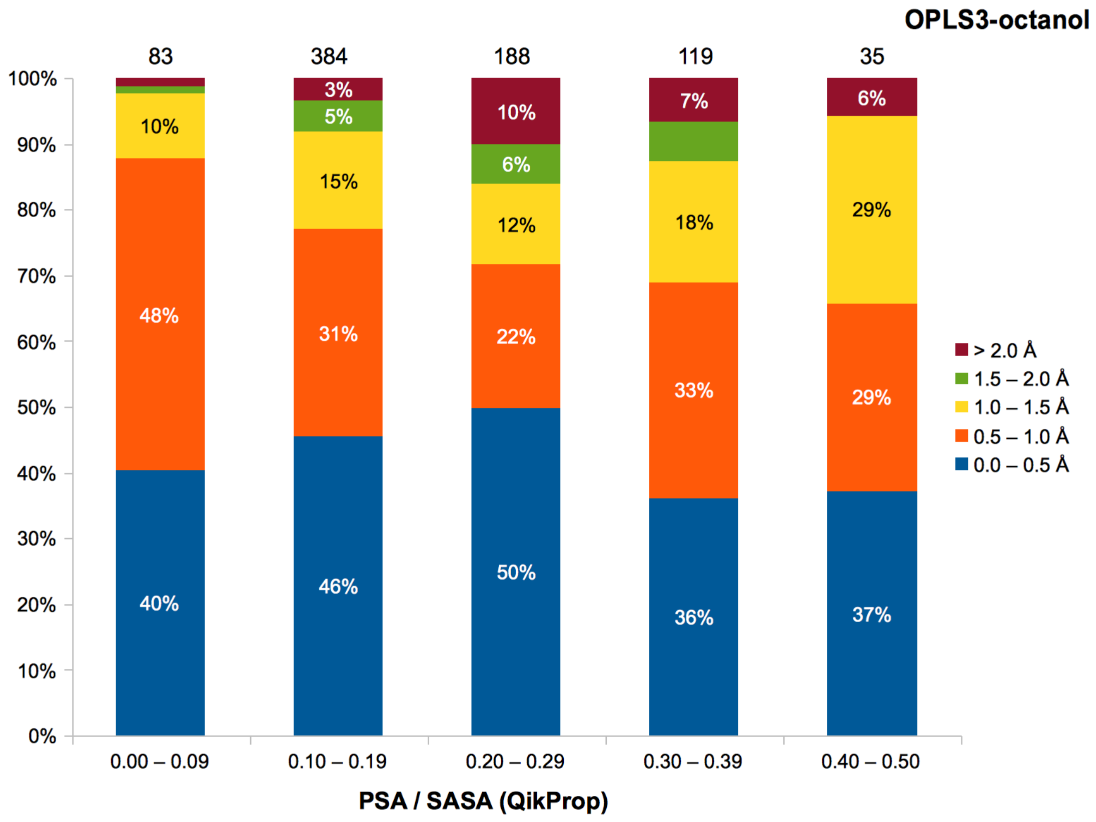


**Figure S8**

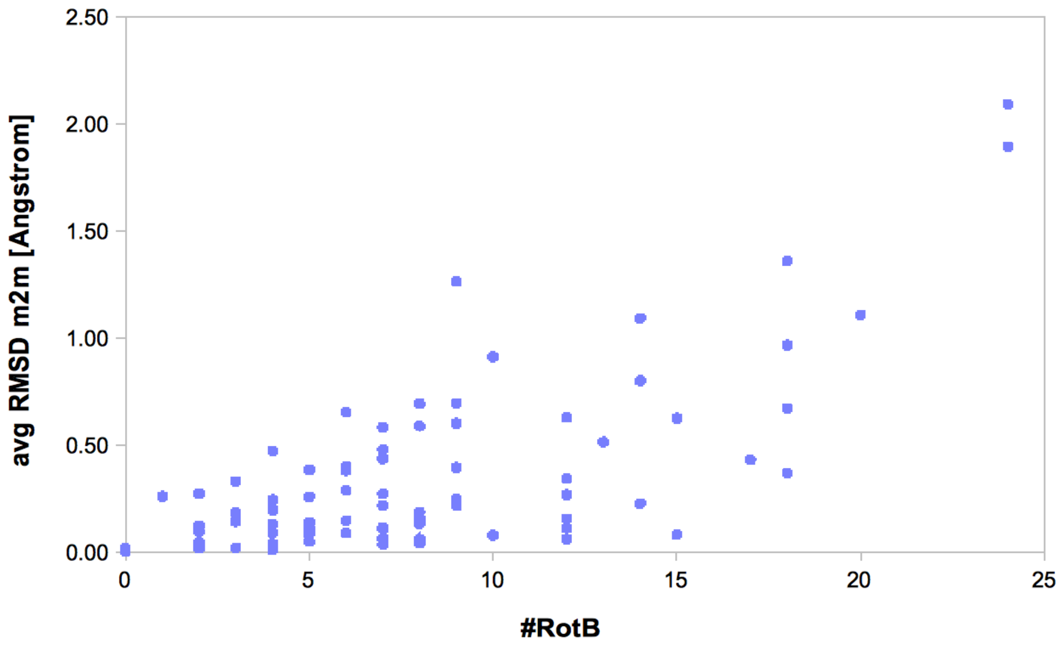


**Figure S9**

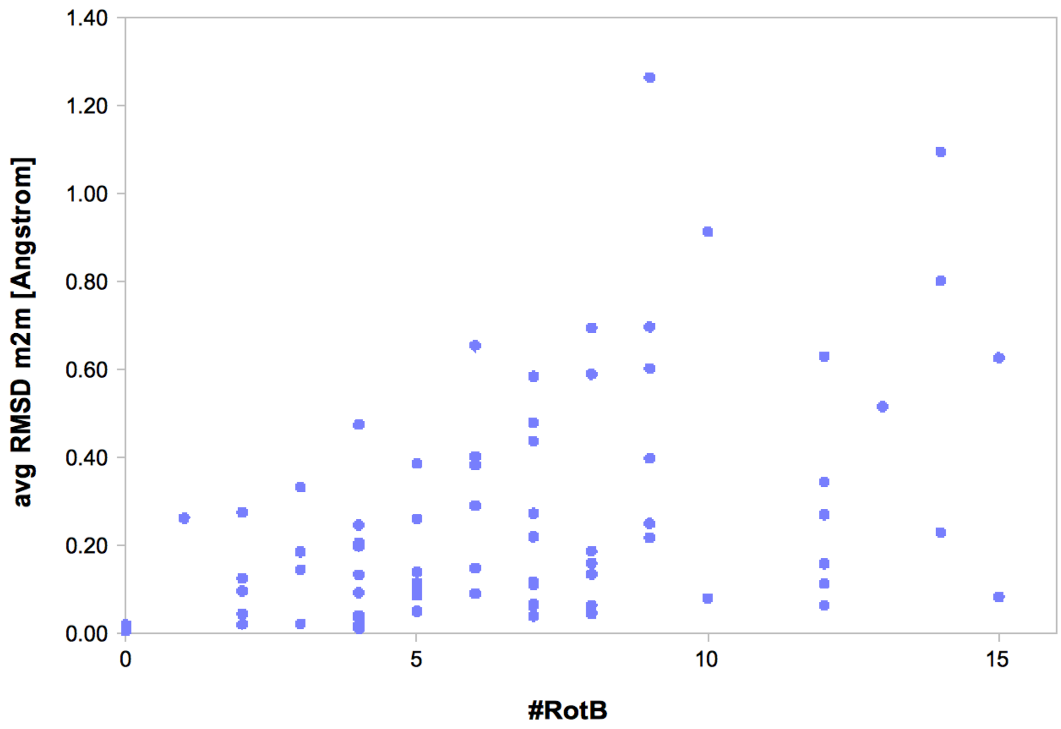


**Figure S10**

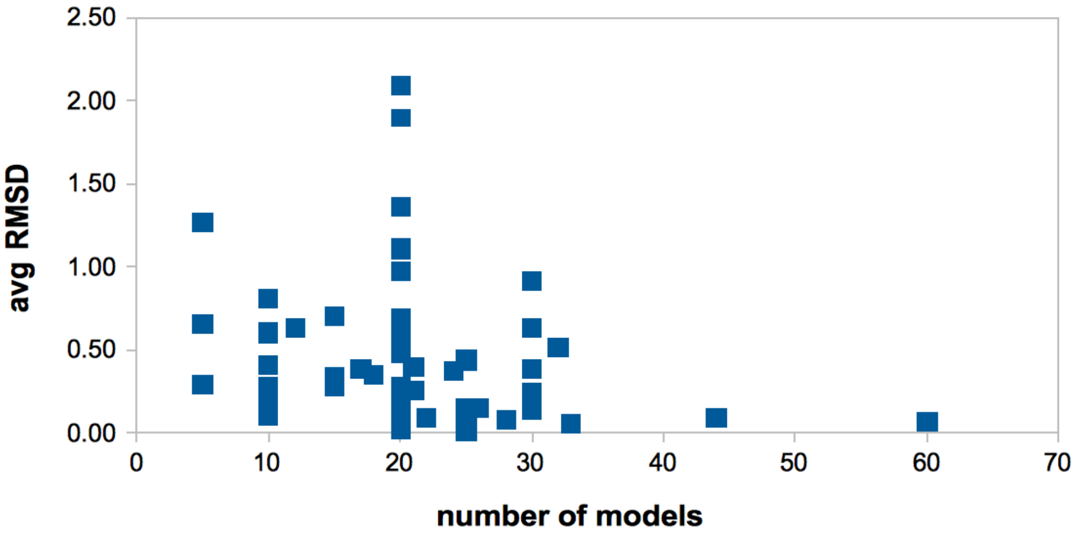


**Figure S11**

Figures S12 – S48 correspond to "Charged group analyses" section in the main text and show best-achieved RMSDs (most similar conformer to the crystal-pose) for ligands grouped by number of rotatable bonds (force field – solvent combination indicated at the top right corner of each figure).

Figures 12 – 24: The input structures for the conformational search were CHARGED.

Figures 25 – 36: The input structures for the conformational search were NEUTRALIZED.

Figures 37 – 48: Outcome of the conformational searches with CHARGED and NEUTRALIZED inputs was combined and best RMSD was selected for each input structure.

Abbreviations used for solvents in the figures: clf – chloroform, oct – Octanol, wat – Water

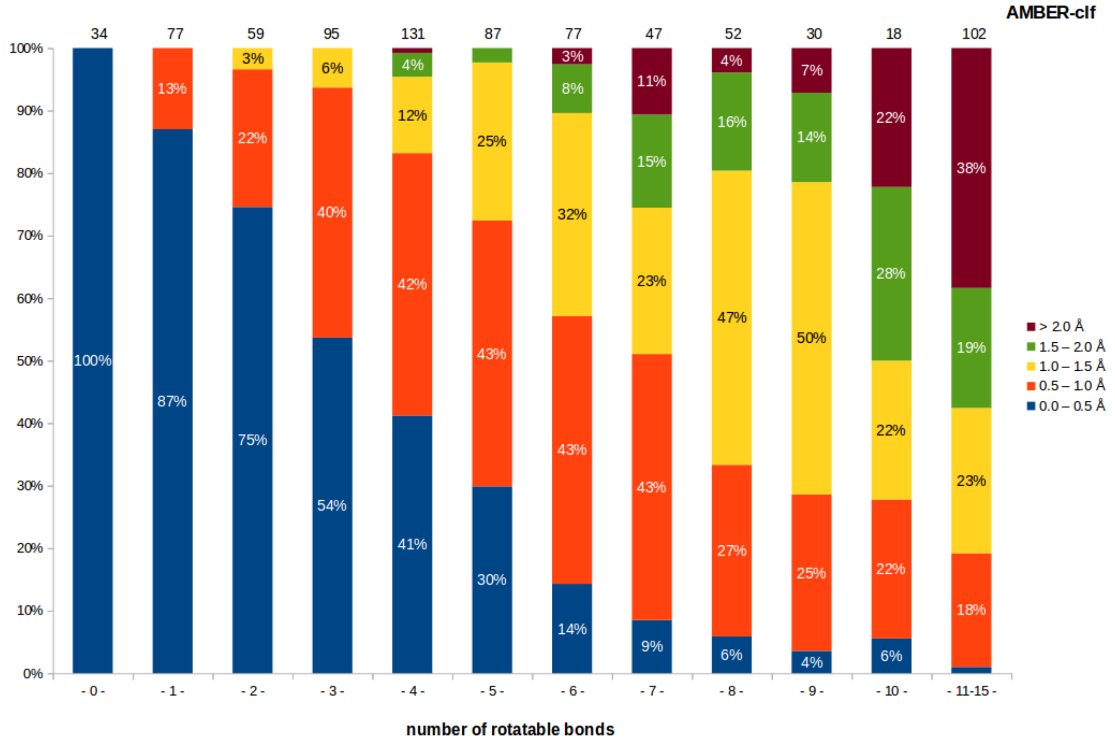


# Figure S12

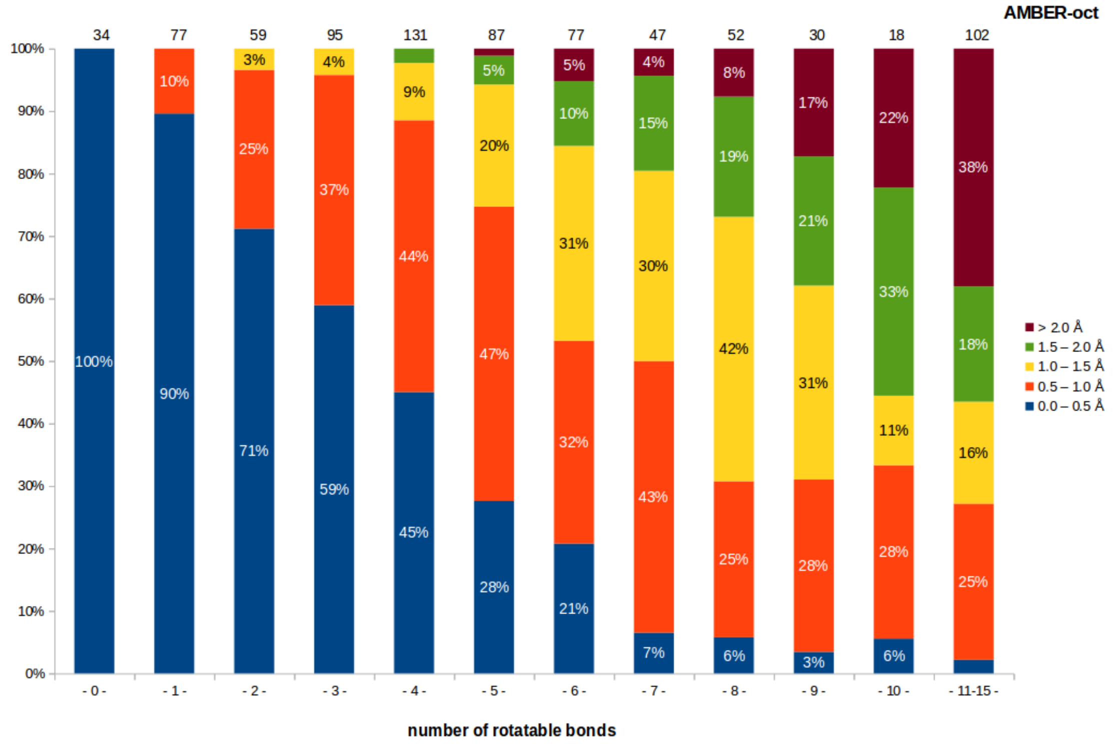


# Figure S13

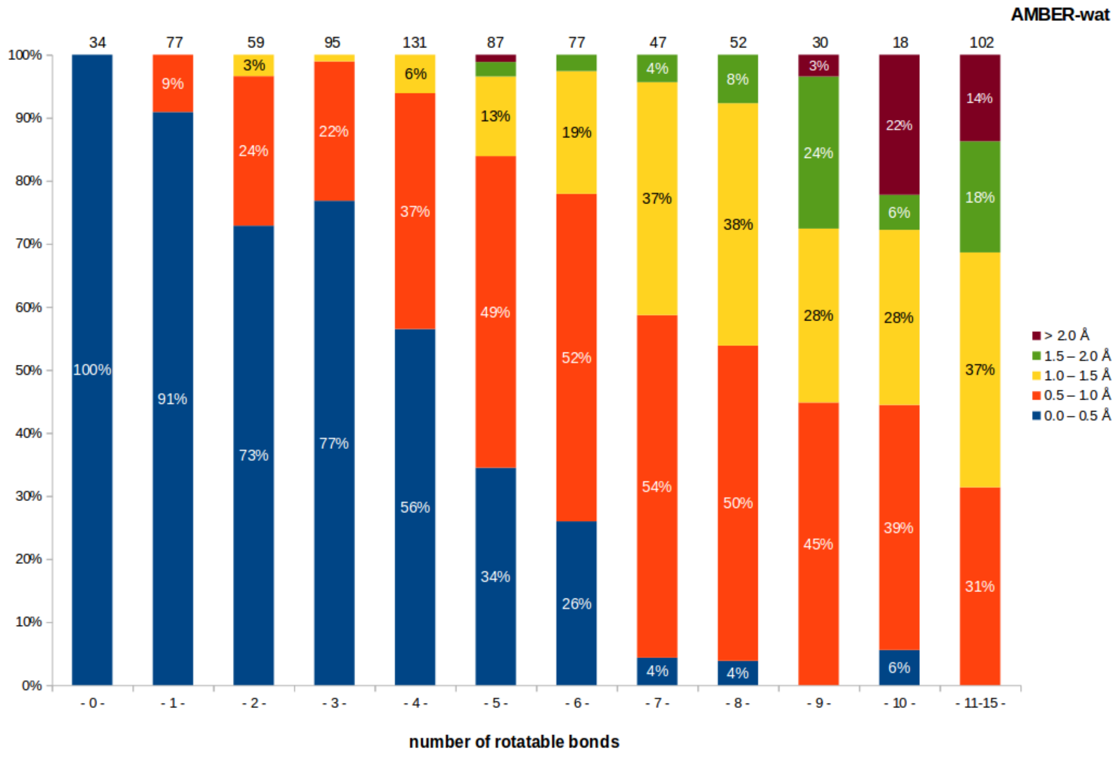


**Figure S14**

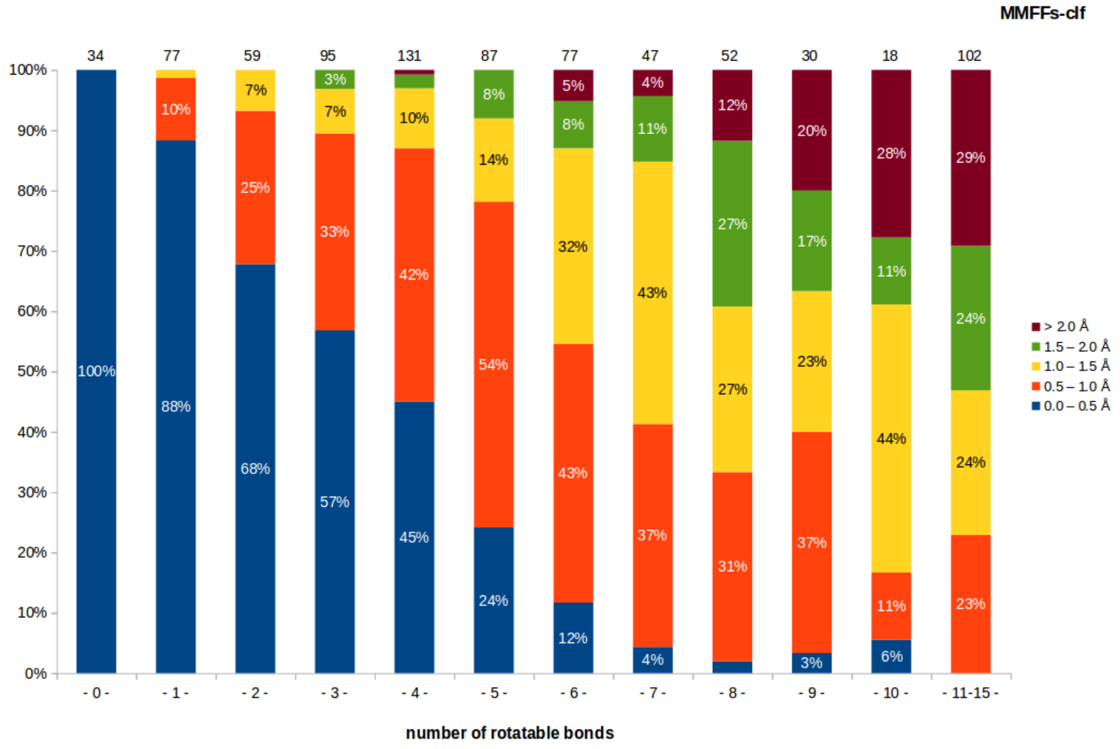


**Figure S15**

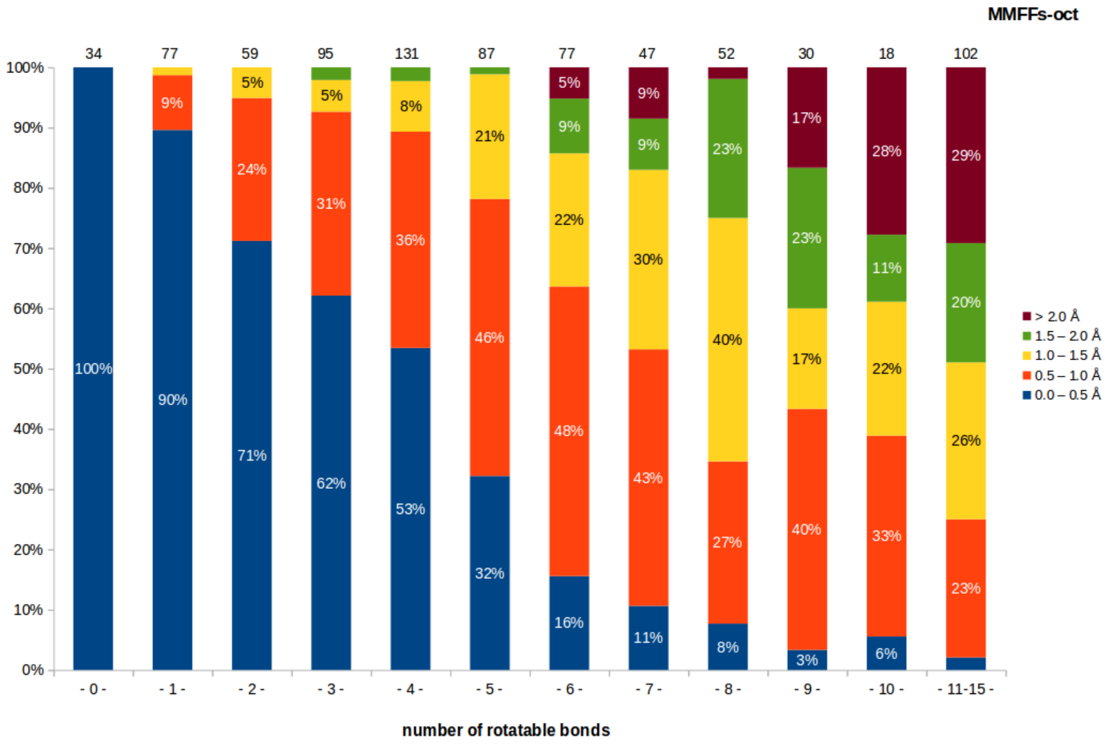


# Figure S16

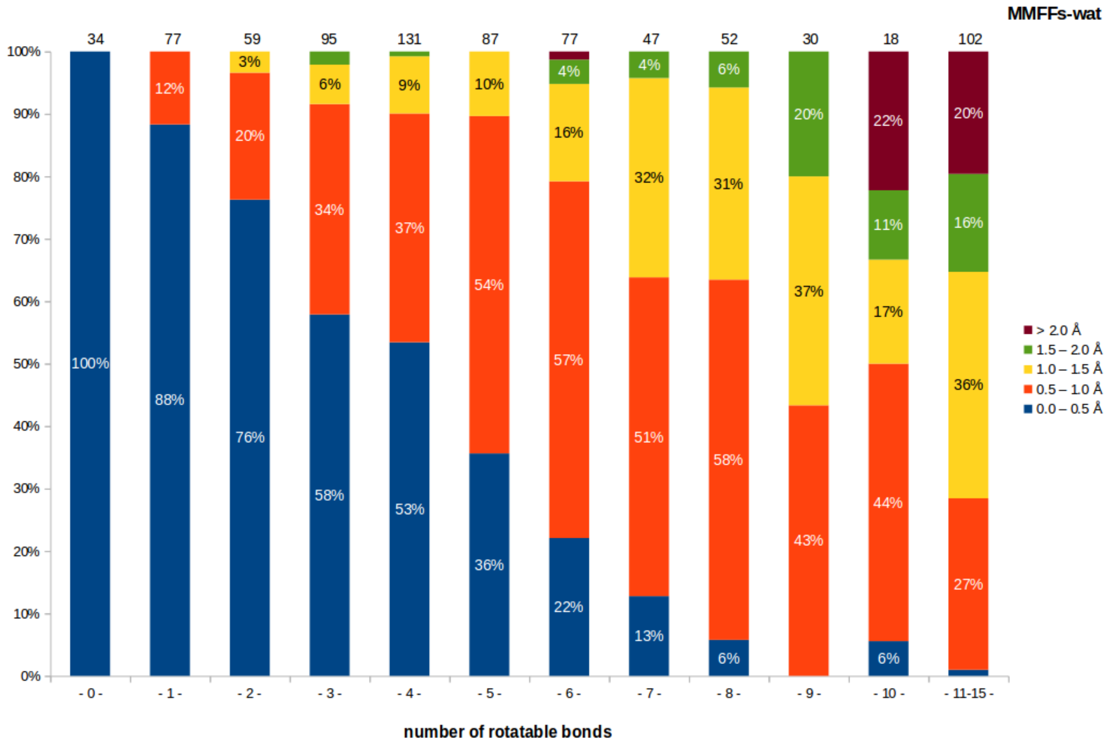


# Figure S17

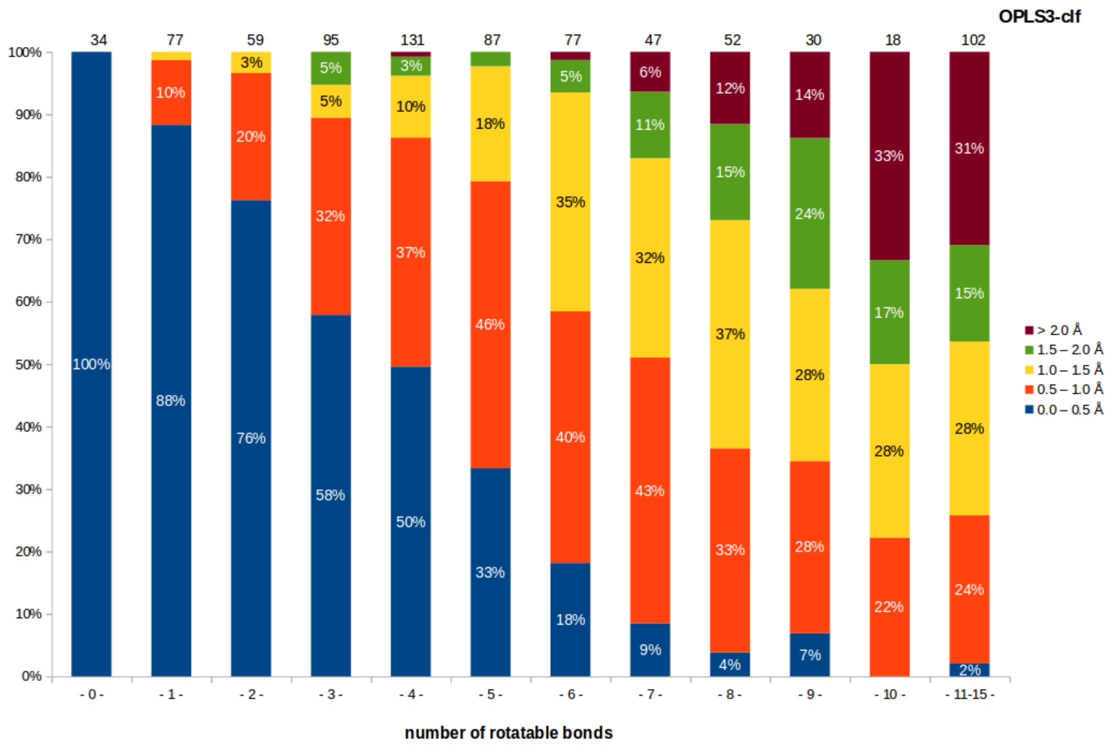


**Figure S18**

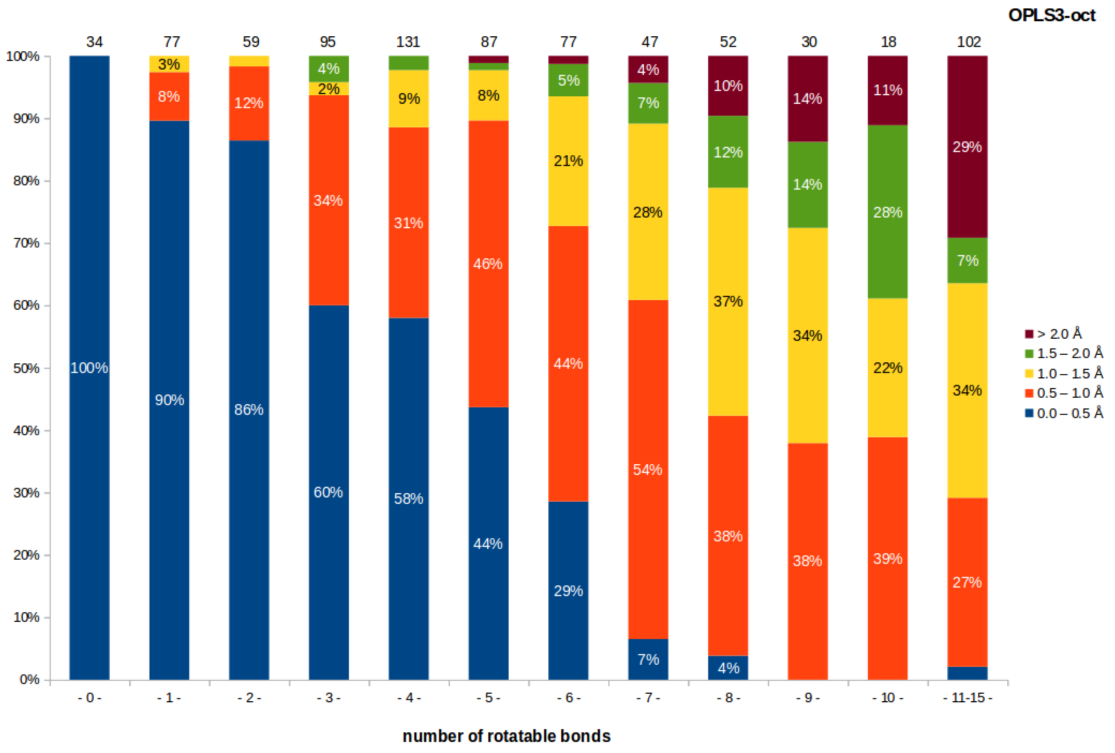


**Figure S19**

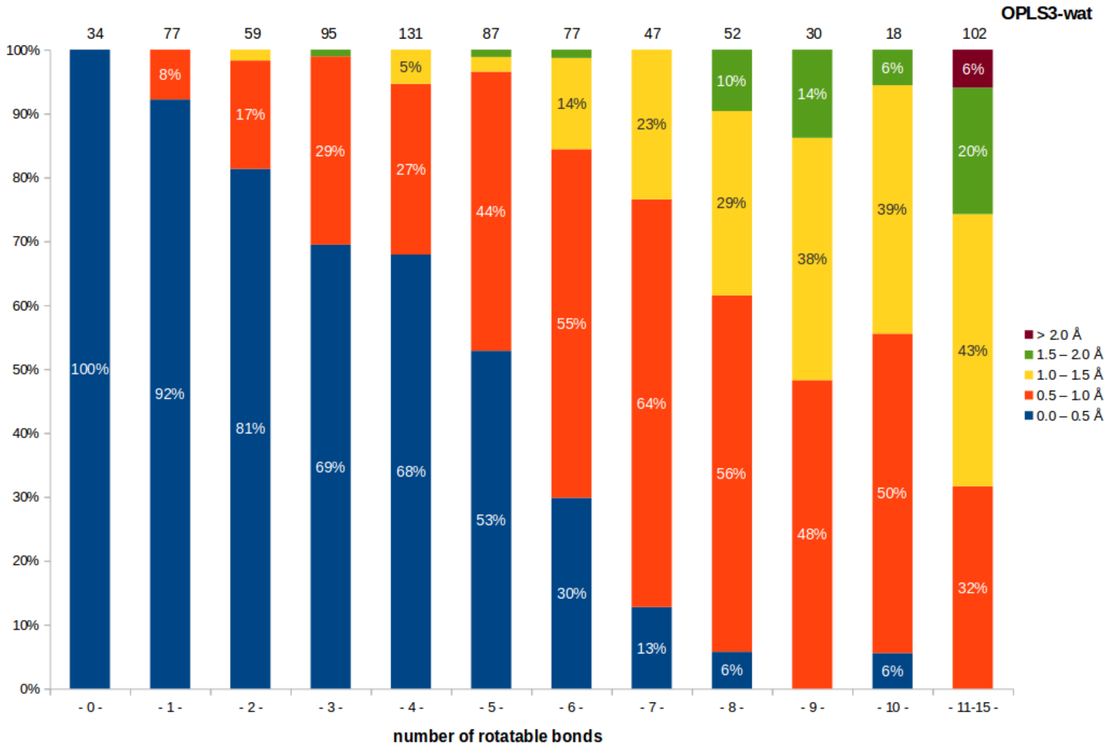


**Figure S20**

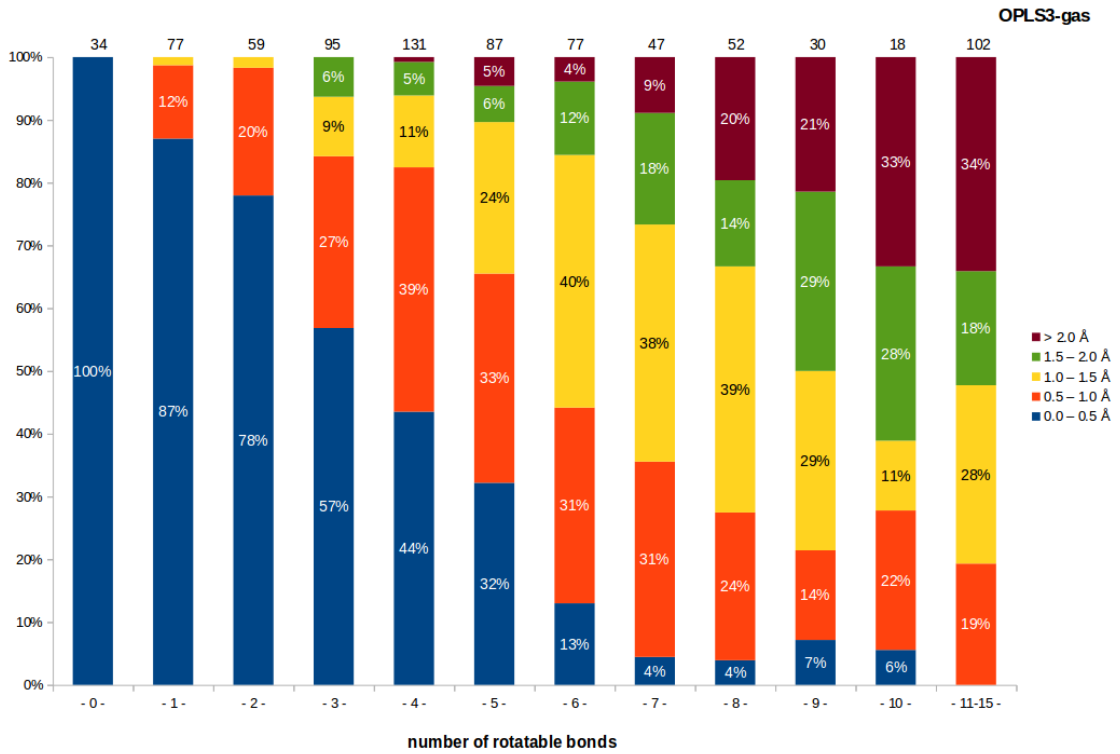


**Figure S21**

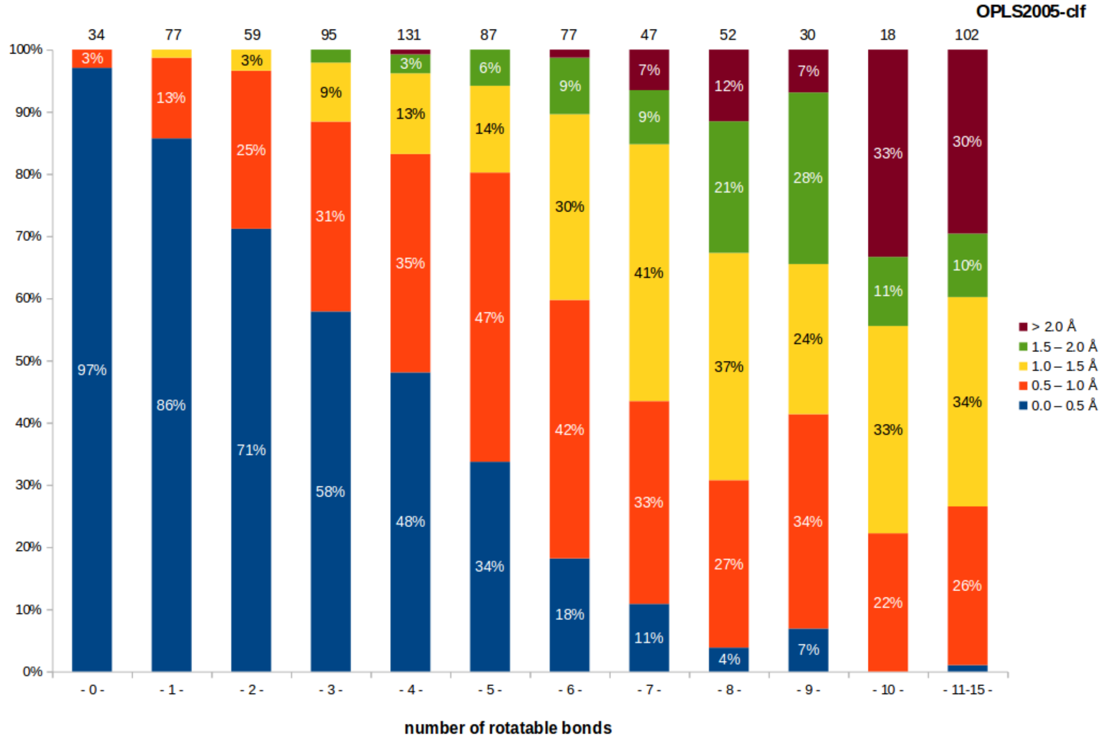


**Figure S22**

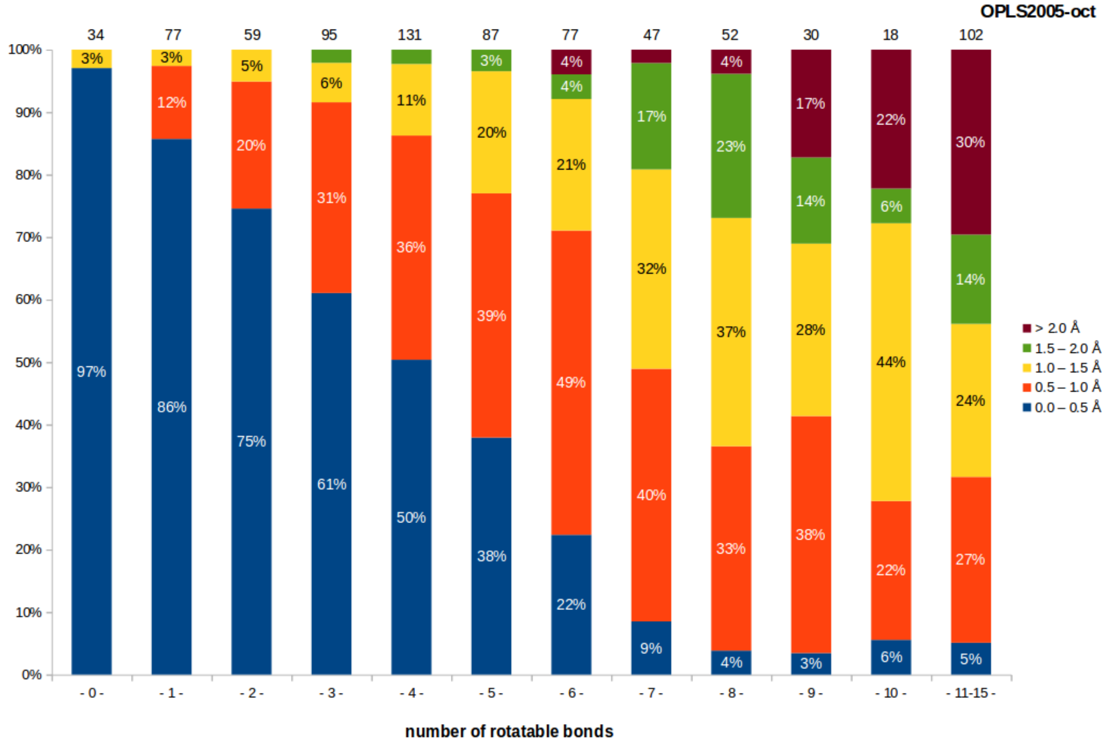


**Figure S23**

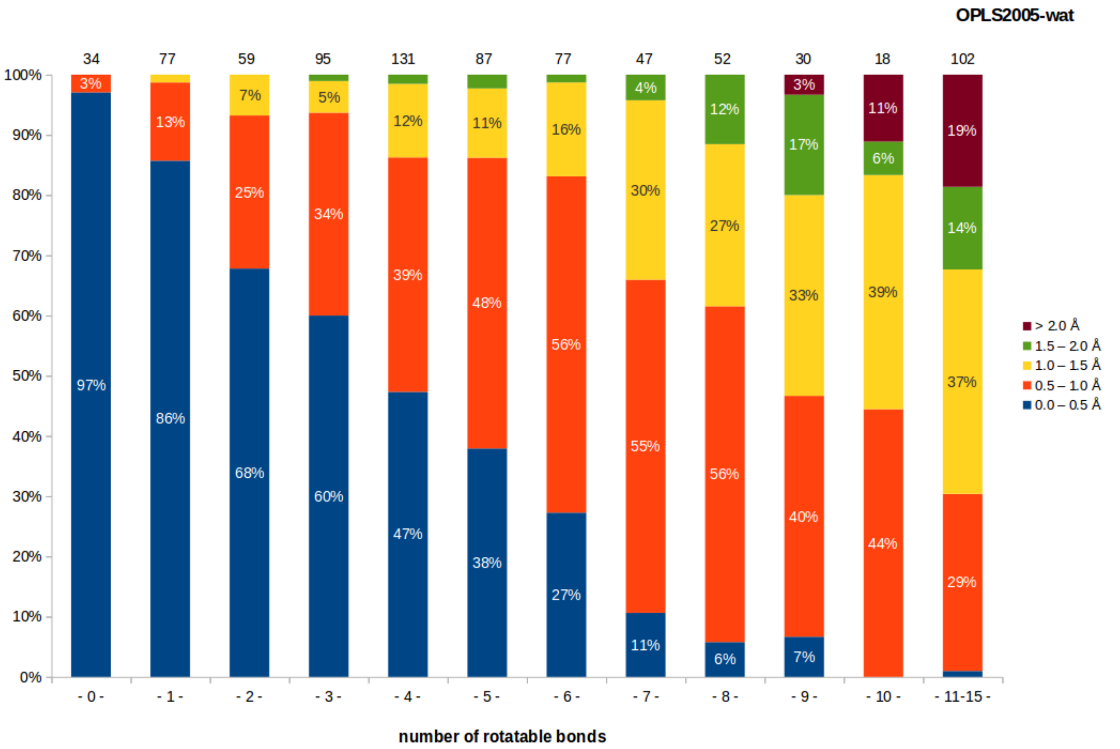


# Figure S24

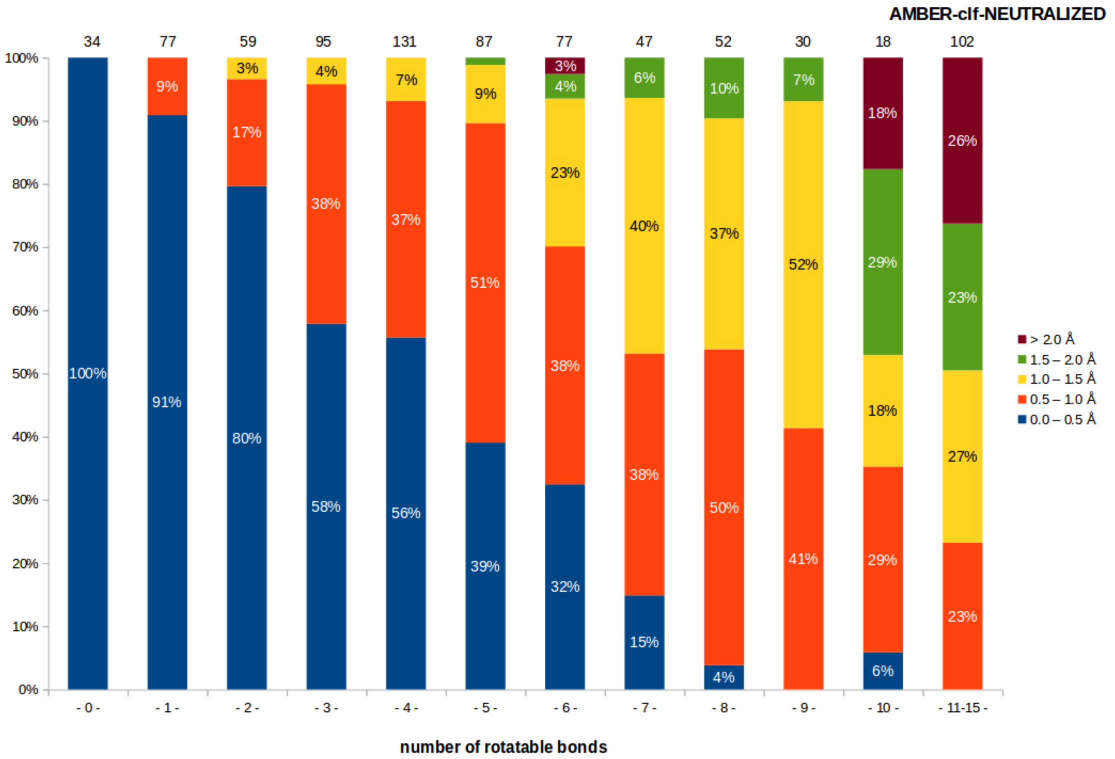


# Figure S25

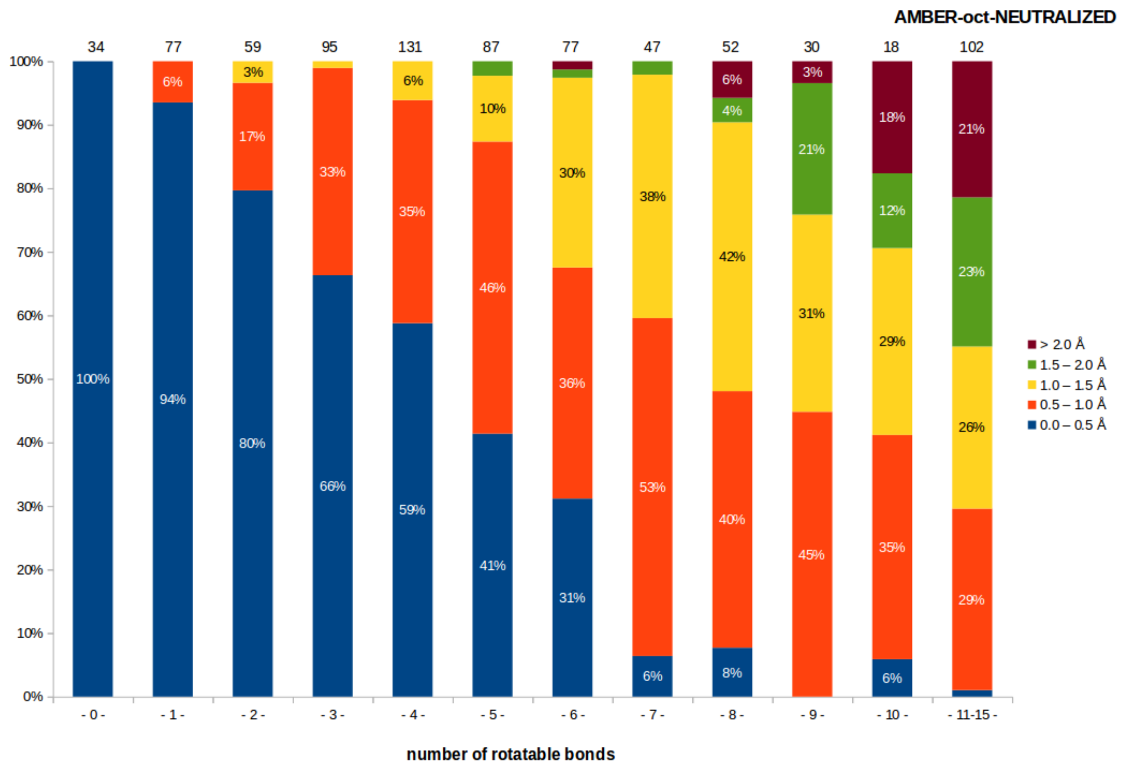


**Figure S26**

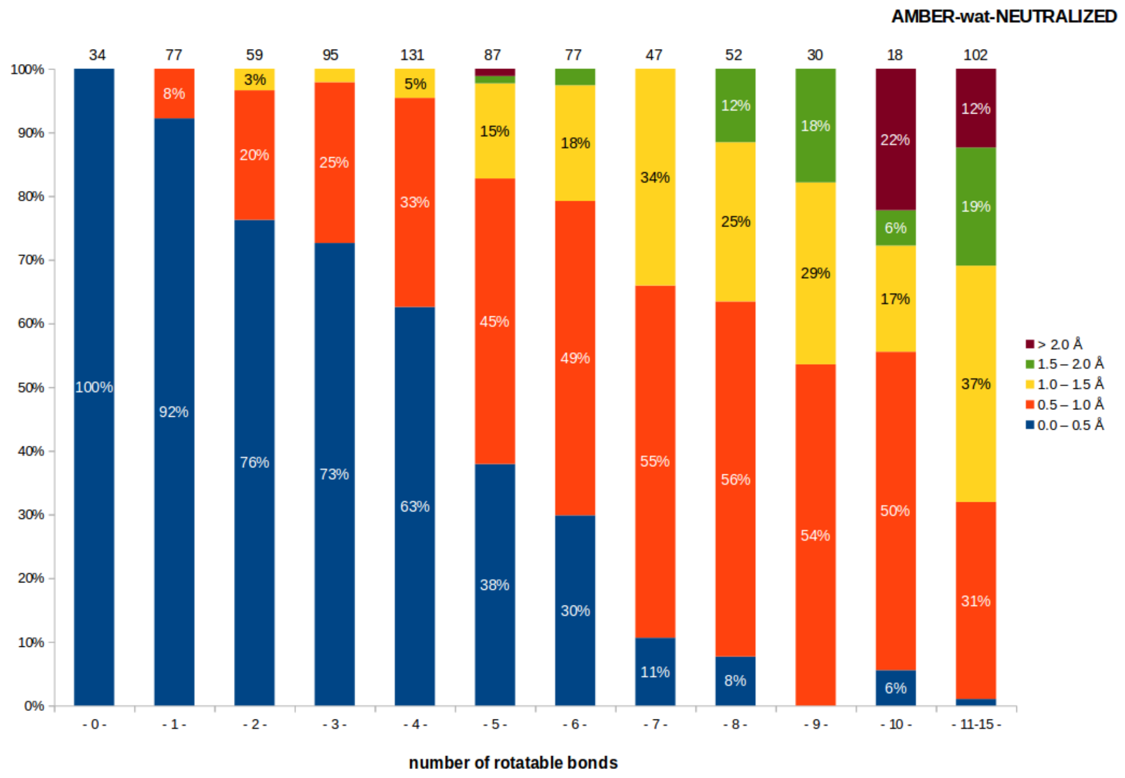


**Figure S27**

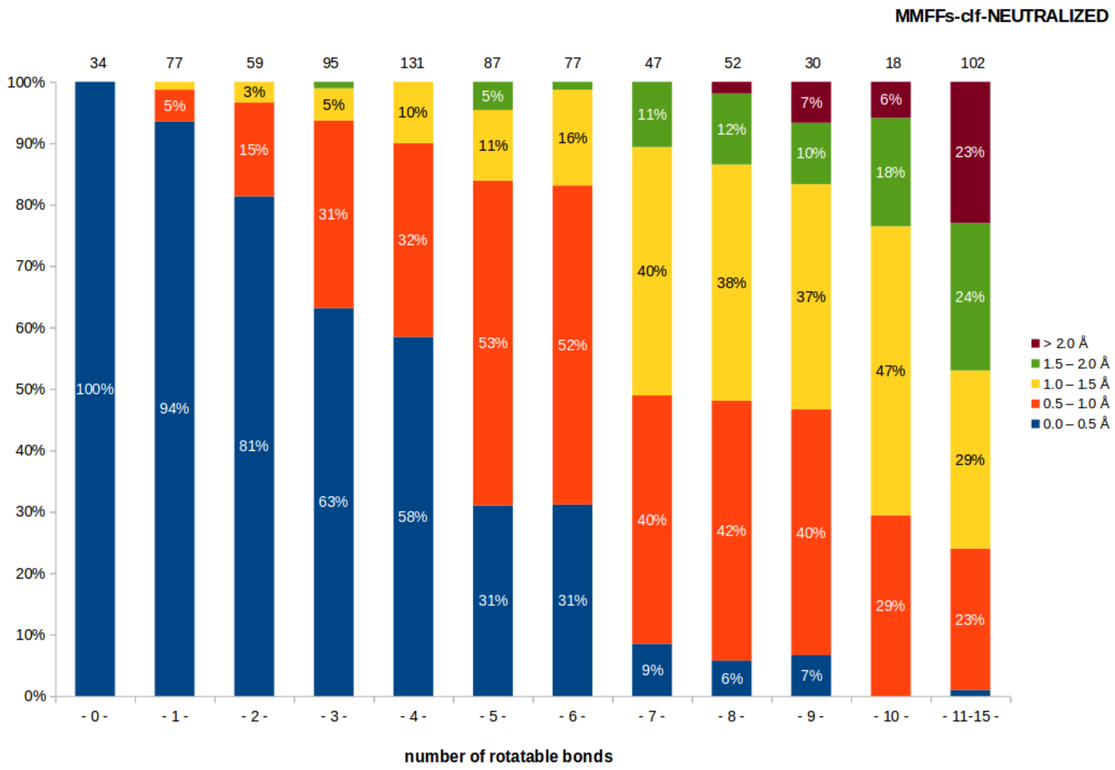


**Figure S28**

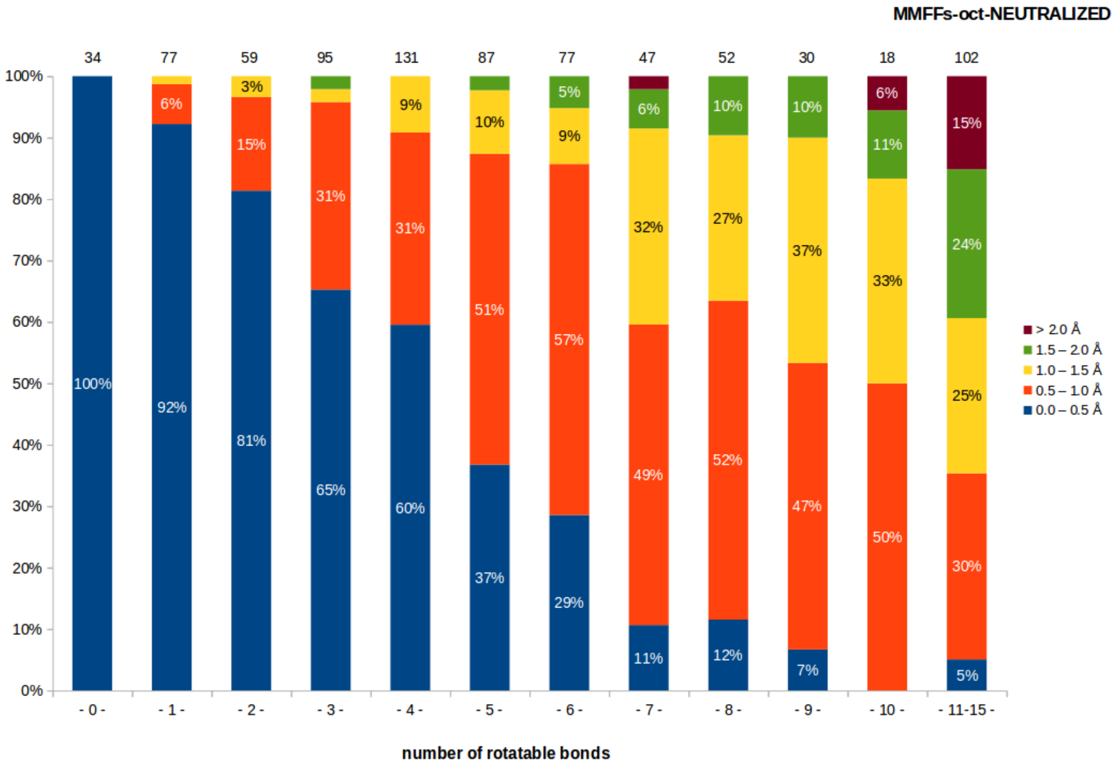


**Figure S29**

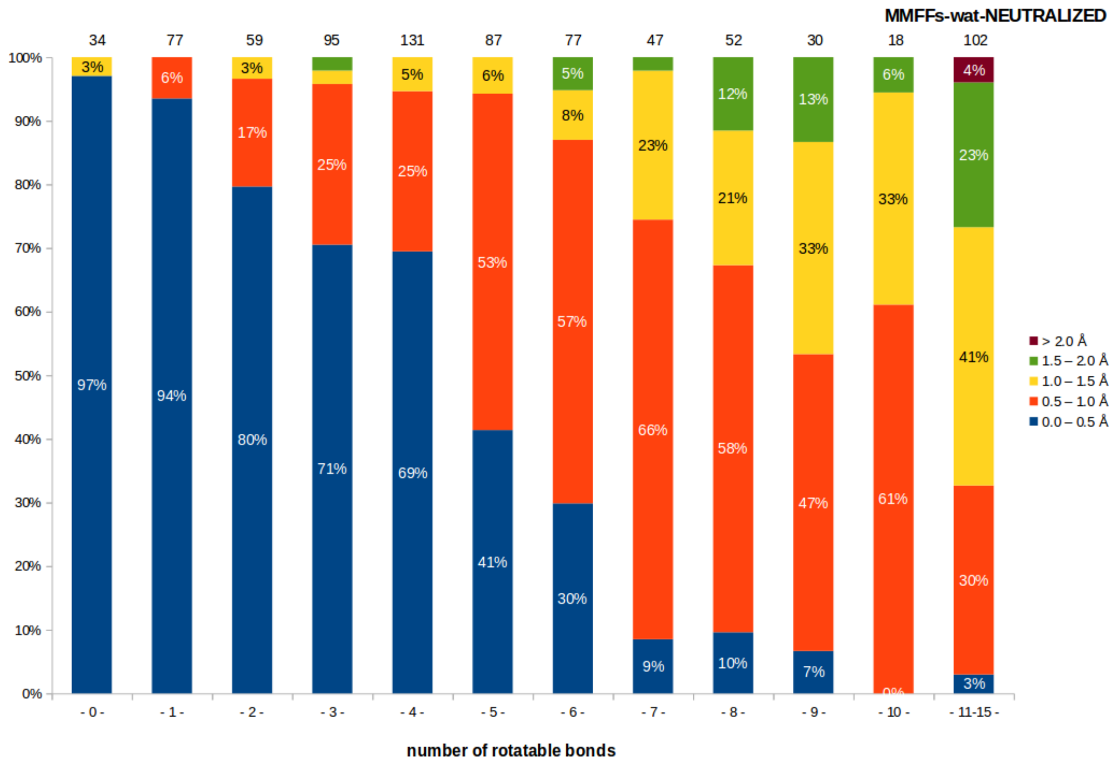


Figure S30

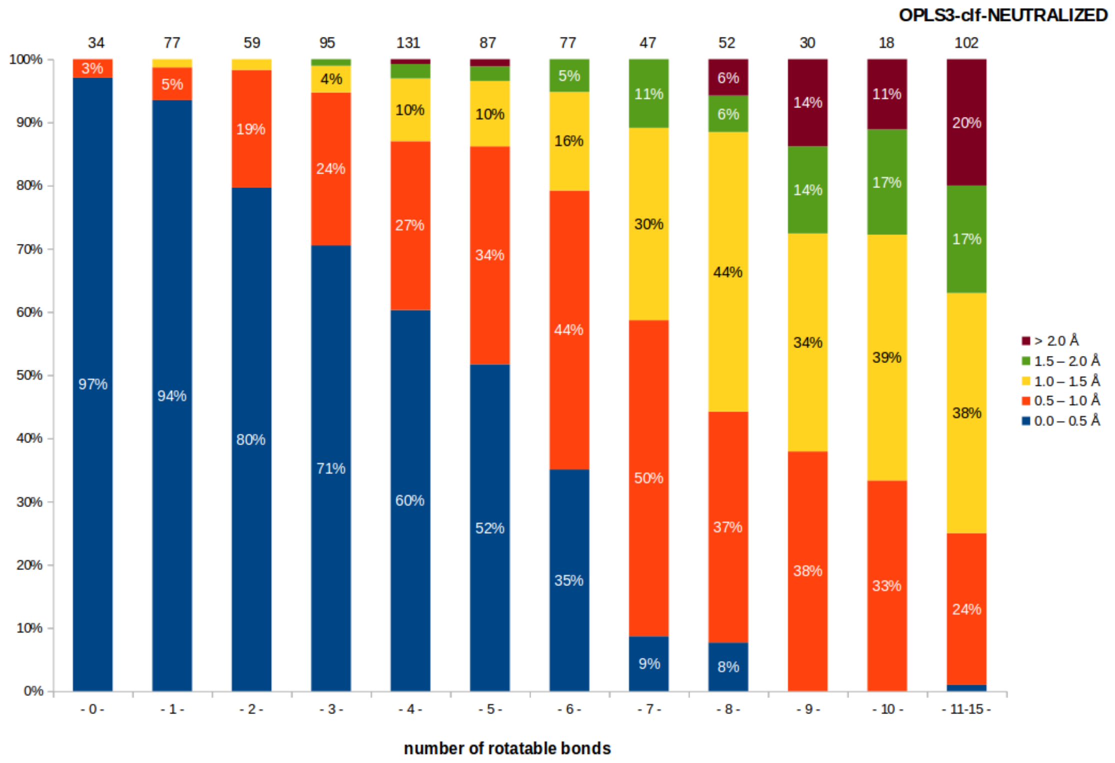


Figure S31

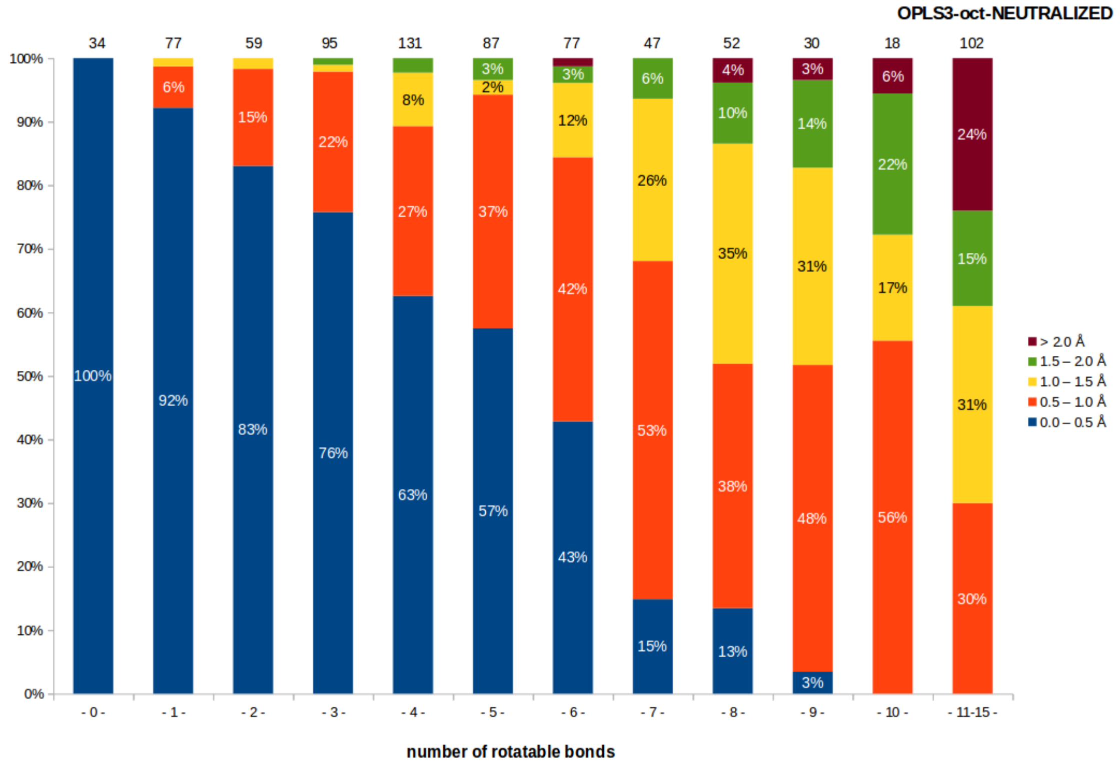


# Figure S32

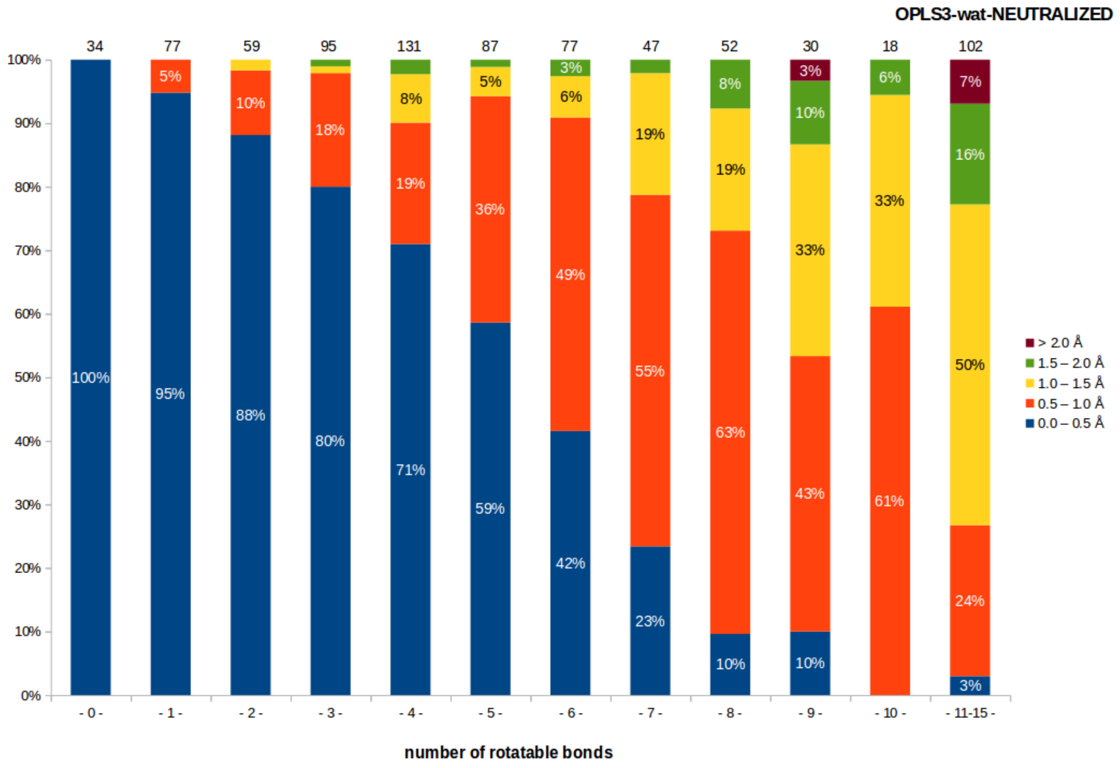


# Figure S33

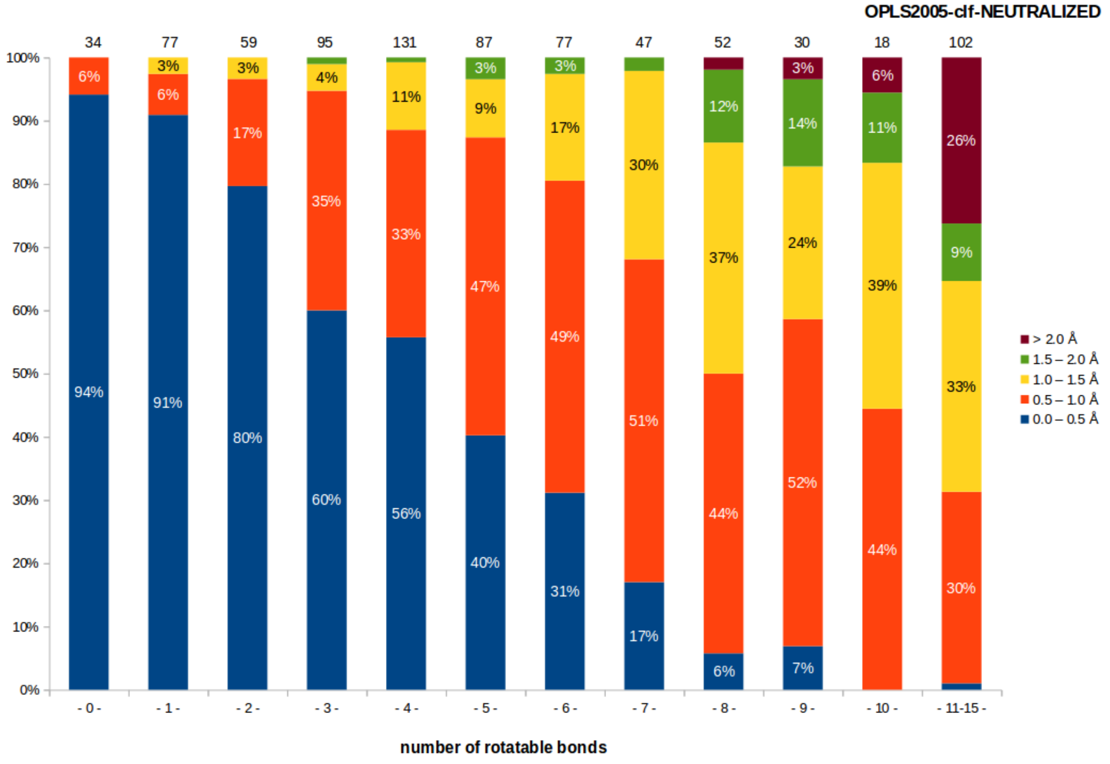


**Figure S34**

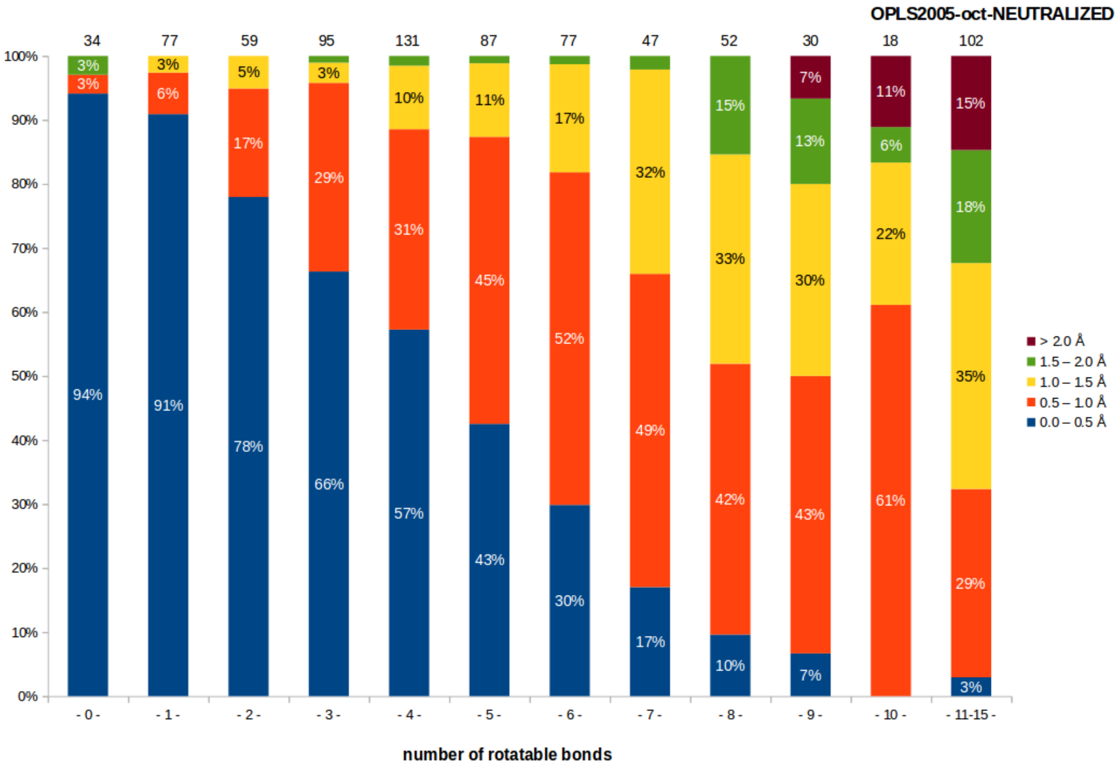


**Figure S35**

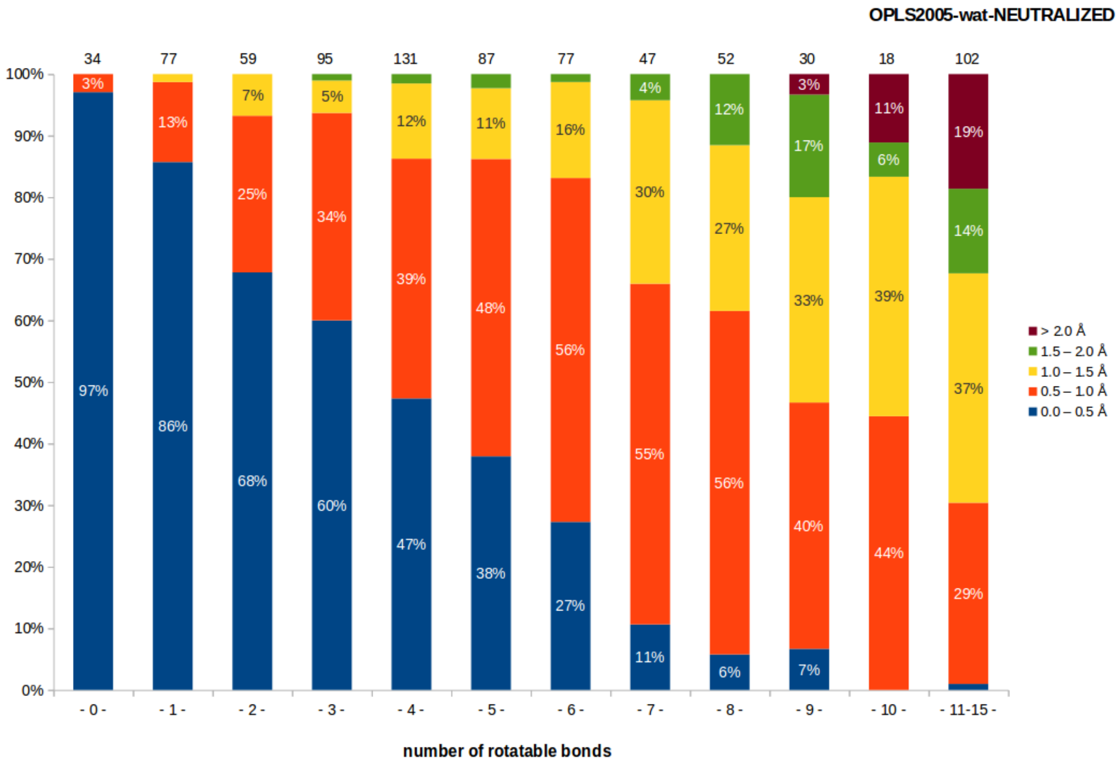


**Figure S36**

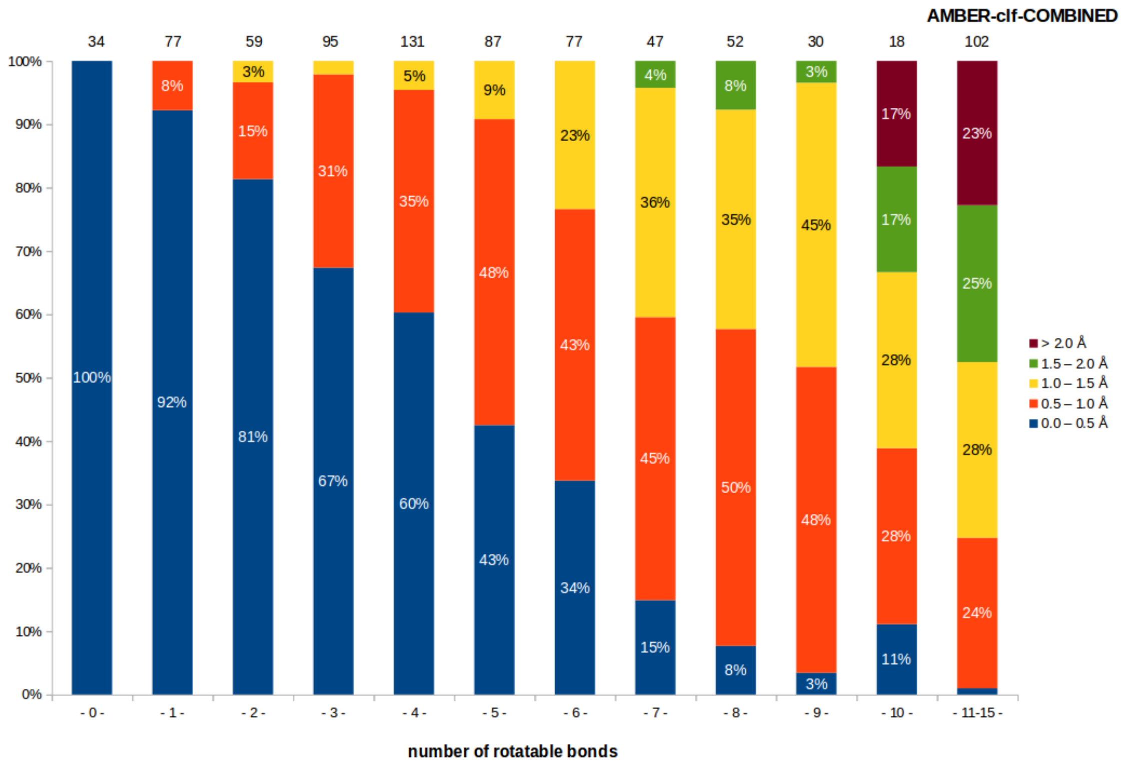


**Figure S37**

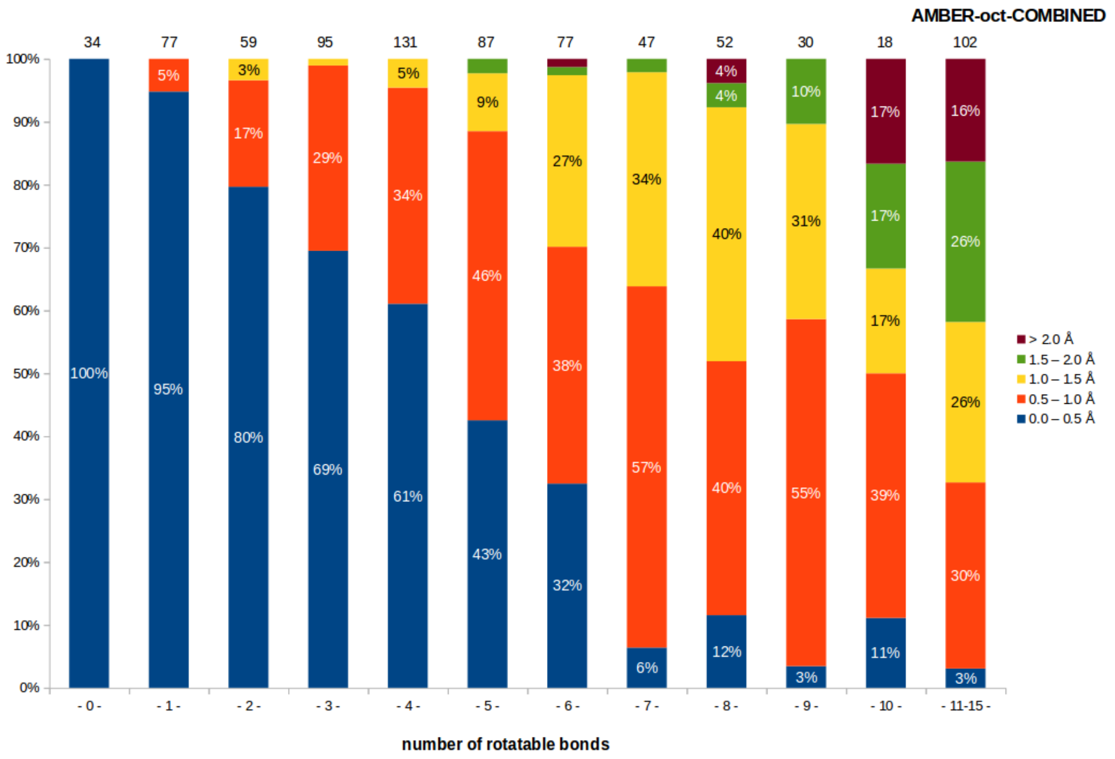


**Figure S38**

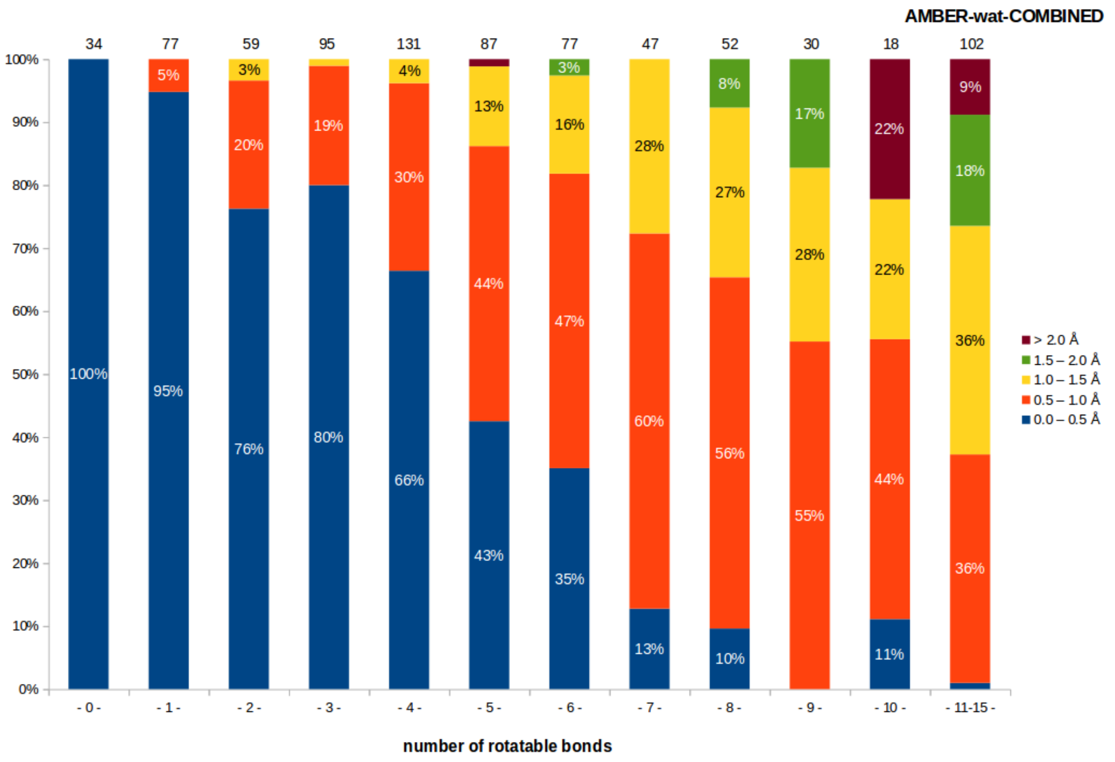


**Figure S39**

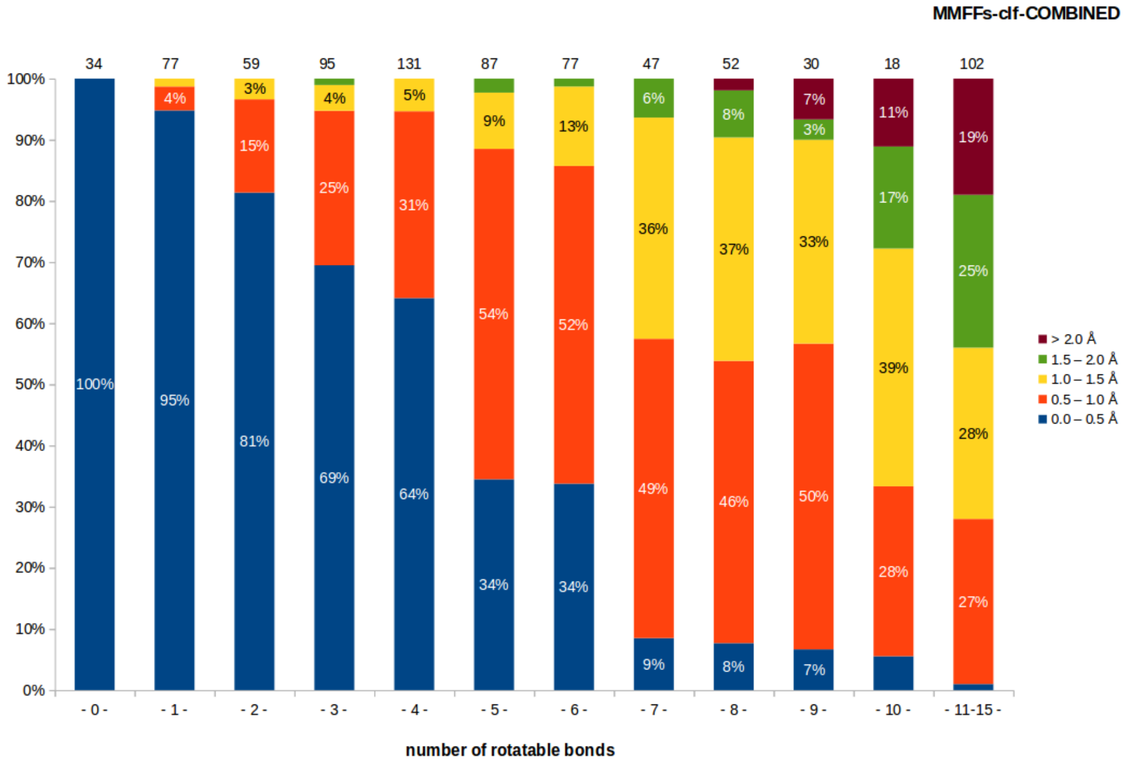


**Figure S40**

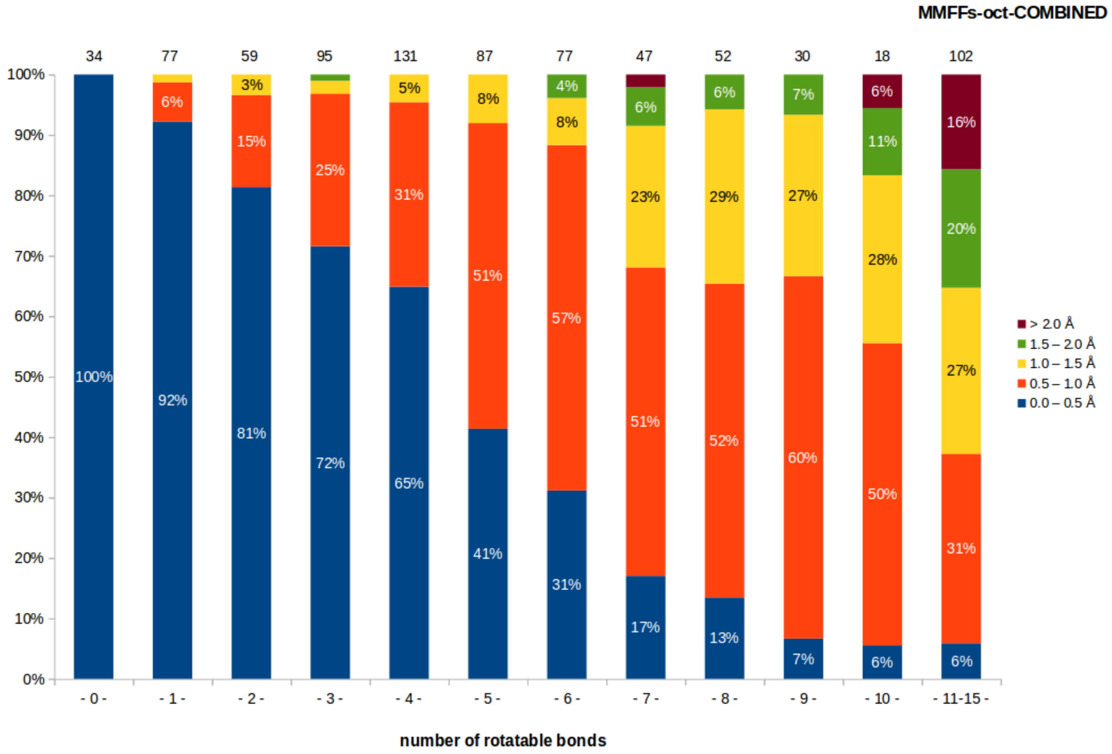


**Figure S41**

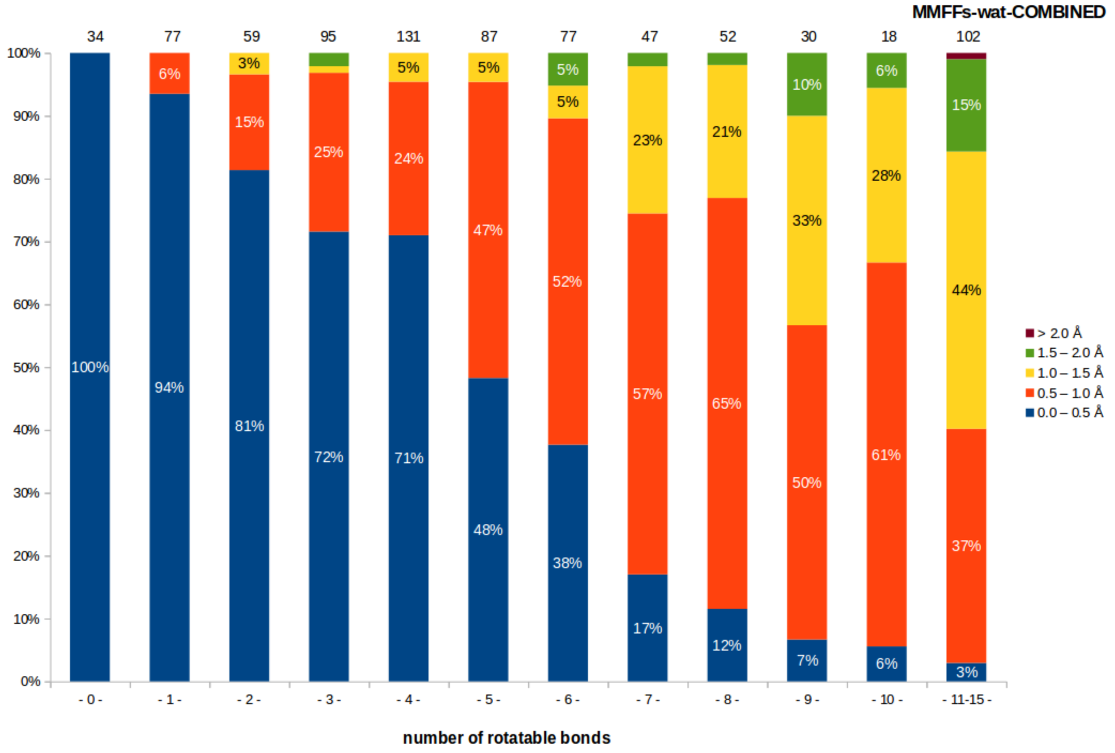


**Figure S42**

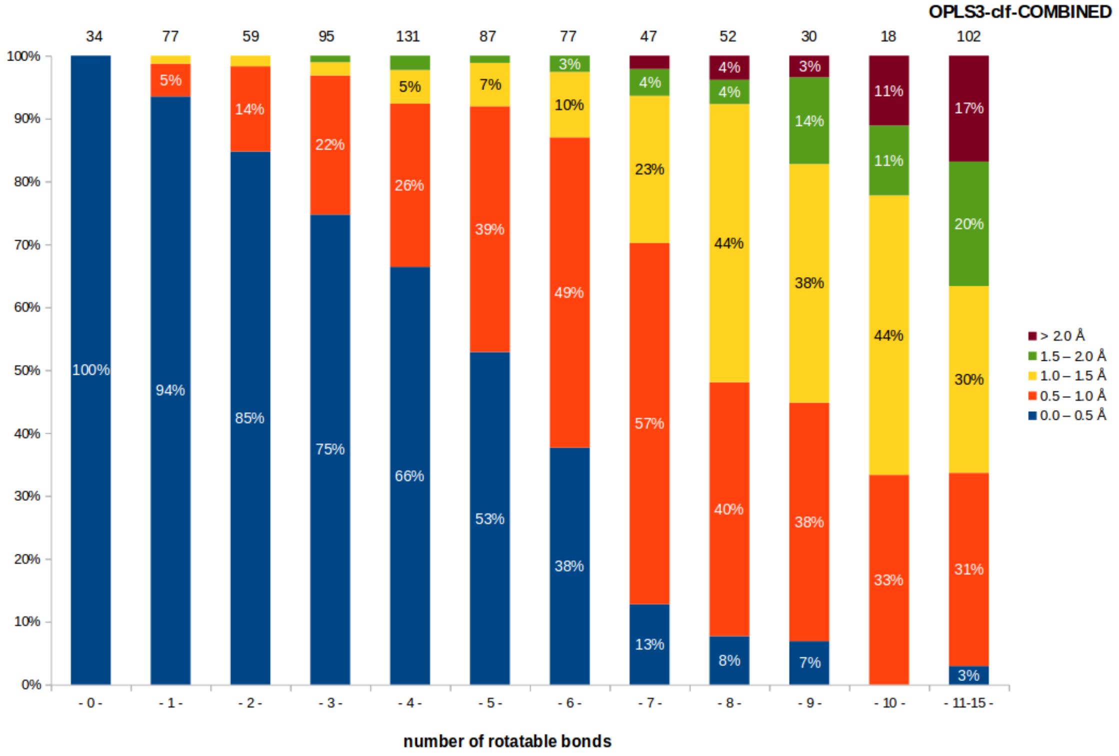


**Figure S43**

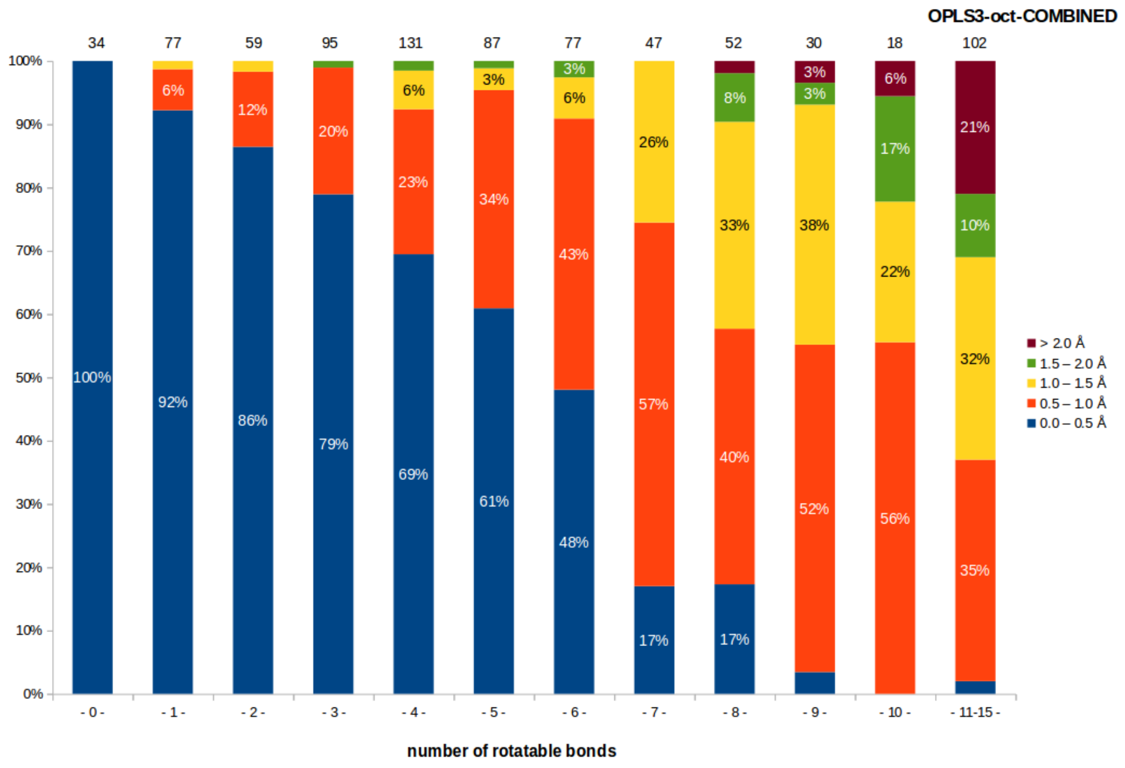


# Figure S44

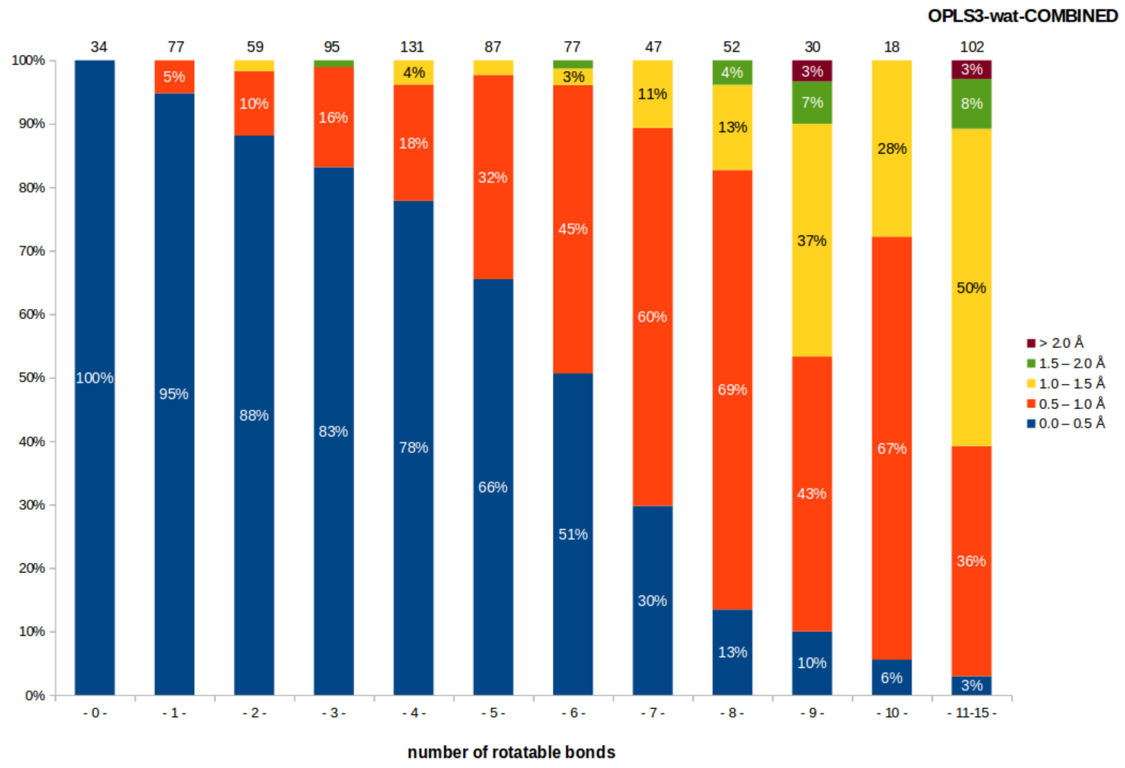


# Figure S45

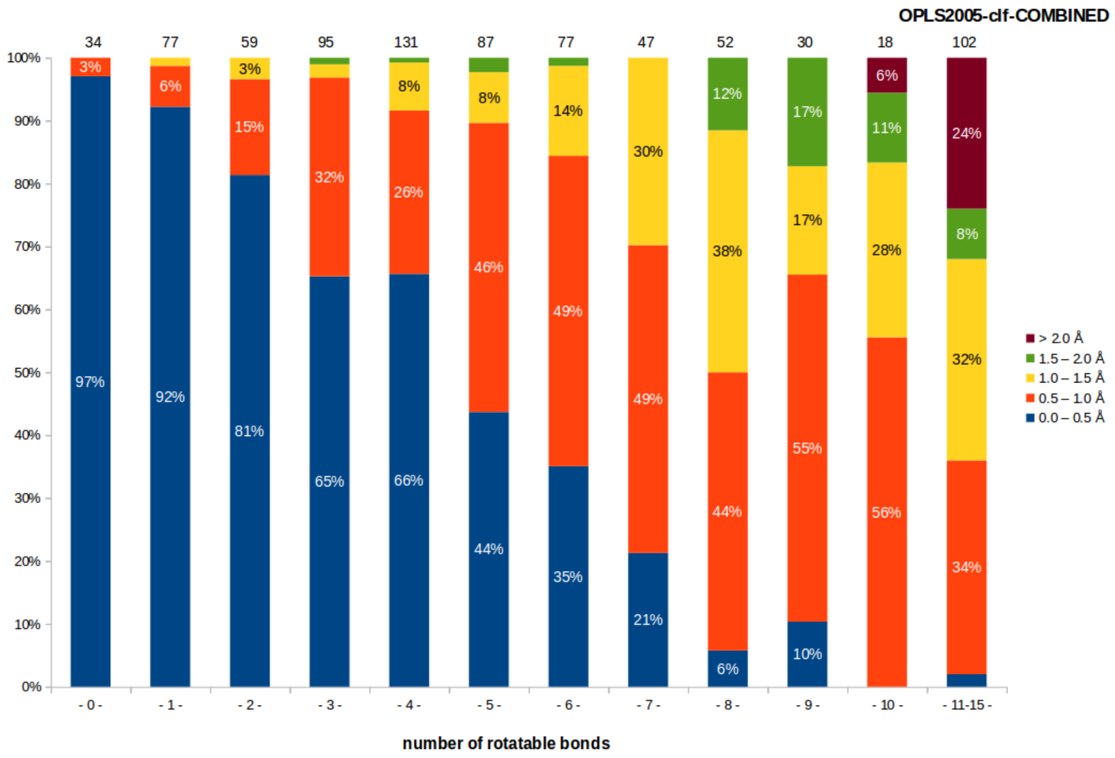


**Figure S46**

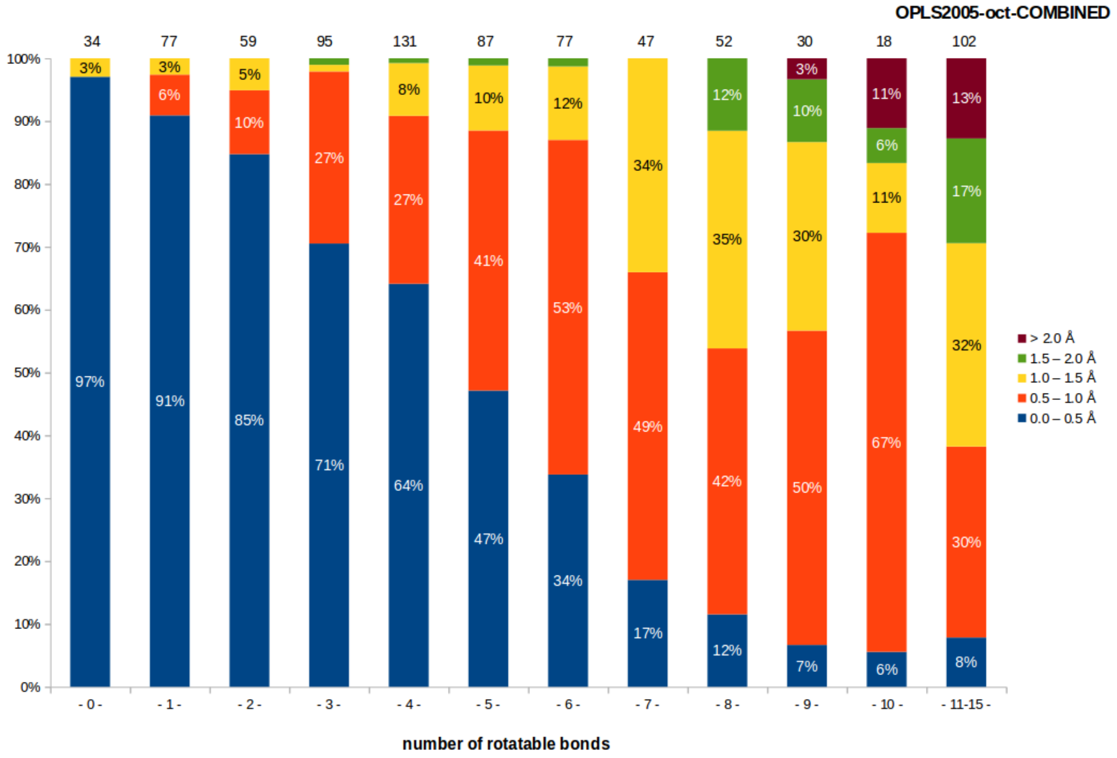


**Figure S47**

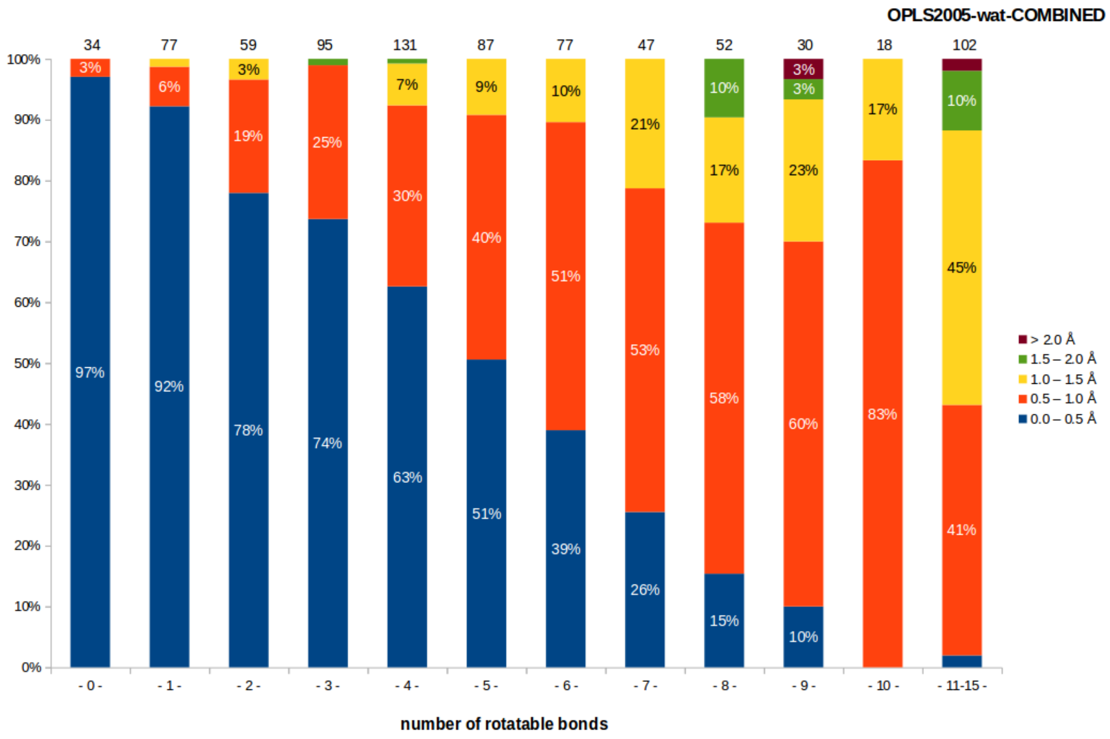
OPLS2005-wat-COMBINED
34
77
59
95
131
87
77
47
52
30
18
102
100%
90%
80%
70%
60%
50%
40%
30%
20%
10%
0%
97%
3%
92%
6%
78%
19%
3%
74%
25%
63%
30%
7%
51%
40%
9%
39%
51%
10%
26%
53%
21%
15%
58%
17%
10%
10%
60%
23%
3%
3%
83%
17%
41%
45%
10%
> 2.0 Å
1.5 – 2.0 Å
1.0 – 1.5 Å
0.5 – 1.0 Å
0.0 – 0.5 Å
- 0 -
- 1 -
- 2 -
- 3 -
- 4 -
- 5 -
- 6 -
- 7 -
- 8 -
- 9 -
- 10 -
- 11-15 -
number of rotatable bonds

**Figure S48**
